# NaI/PPh_3_-catalyzed visible-light-mediated decarboxylative radical cascade cyclization of *N*-arylacrylamides for the efficient synthesis of quaternary oxindoles

**DOI:** 10.3762/bjoc.19.5

**Published:** 2023-01-16

**Authors:** Dan Liu, Yue Zhao, Frederic W Patureau

**Affiliations:** 1 Institute of Organic Chemistry, RWTH Aachen University, Landoltweg 1, 52074 Aachen, Germany

**Keywords:** decarboxylative cascade cyclization, iodide catalysis, metal-free photocatalysis, oxindole, phosphine catalysis

## Abstract

A practical NaI/PPh_3_-catalyzed decarboxylative radical cascade cyclization of *N*-arylacrylamides with redox-active esters is described, which is mediated by visible light irradiation. A wide range of substrates bearing different substituents and derived from ubiquitous carboxylic acids, including α-amino acids, were synthesized and examined under this very mild, efficient, and cost effective transition-metal-free synthetic method. These afforded various functionalized oxindoles featuring a C3 quaternary stereogenic center. Mechanistic experiments suggest a radical mechanism.

## Introduction

**Figure 1:**
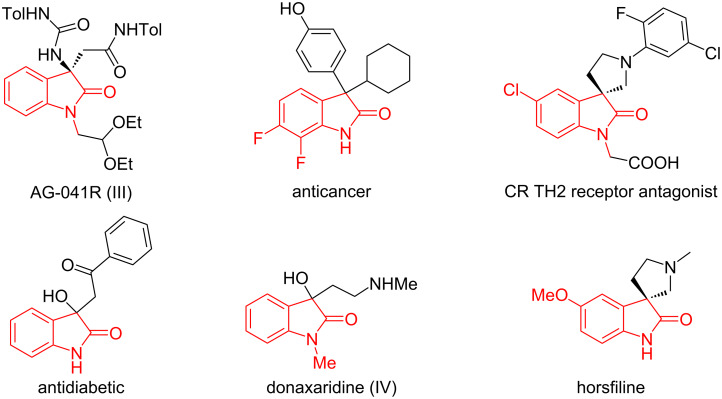
Representative natural products and biologically active molecules containing an oxindole moiety [7–13].

Radical-initiated cascade reactions constitute a powerful synthetic approach to construct multiple C–C or C–X bonds in one pot. As such, these tend to allow facile access to many complex natural molecules and drugs [1–6]. Recently, radical-initiated cascade cyclizations involving acrylamides have attracted considerable attention due to their propensity to build important oxindole scaffolds. These are broadly found in natural products, pharmaceuticals, and bioactive molecules (Figure 1) [7–13]. Although a number of synthetic approaches have already been explored [14–20], these existing methods generally require stoichiometric, often onerous reagents [21–28], and/or high temperatures [29–38].

**Scheme 1:**
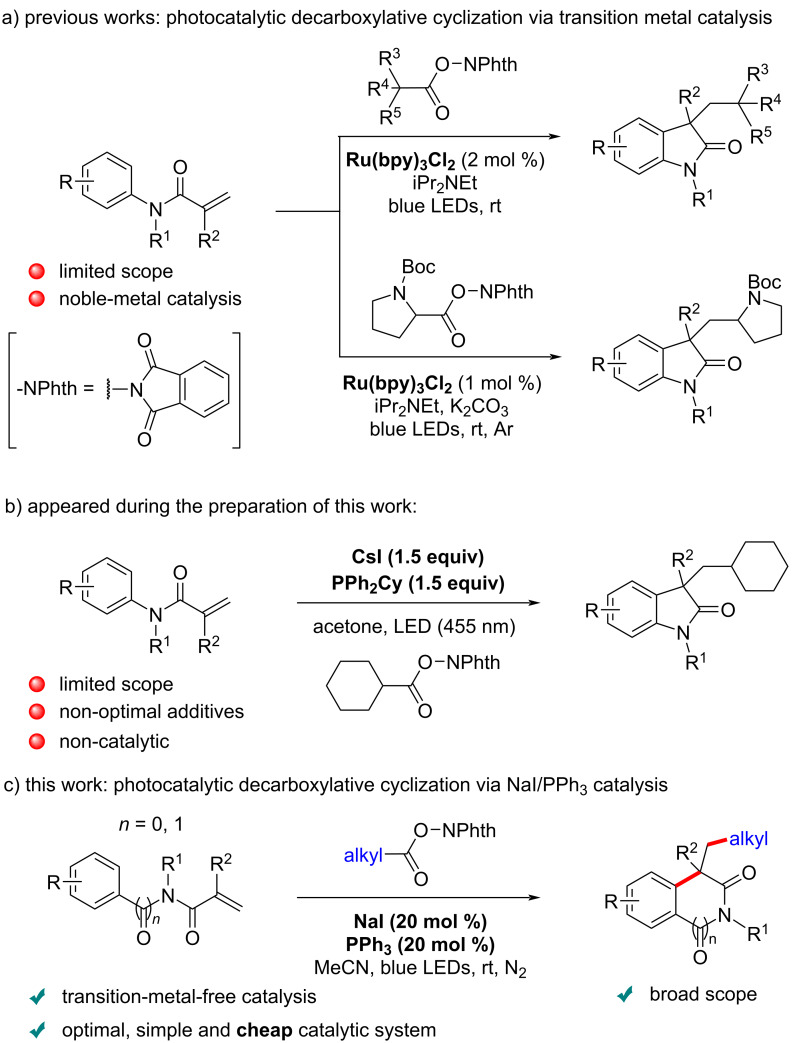
Selected photocatalytic decarboxylative radical cascade reactions of *N*-arylamides.

In the past few years, photocatalytic processes have become one of the most powerful tools in developing radical-initiated addition/cyclization cascades from acrylamides for the synthesis of oxindoles [39–41]. The radicals are typically generated from alkyl halides [42–44], carboxylic acids [45–47], simple alkanes [48], alkylboronic acids [49], isocyanides [50], or other [51–53]. In this context, the group of Fu reported a Ru(bpy)_3_Cl_2_-catalyzed synthesis of *N*-Boc proline oxindole derivatives under visible-light assistance [47]. Therein, *N*-hydroxyphthalimide (NPhth) esters were utilized as alkyl radical precursors, which can be readily prepared from highly available carboxylic acids. In 2015, Cheng and co-workers disclosed a visible light-mediated radical tandem cyclization of *N*-arylacrylamides with *N*-(acyloxy)phthalimides to access 3,3-dialkylated oxindoles in the presence of [Ru(bpy)_3_Cl_2_]·6H_2_O [46]. However, these seminal methods remain limited by the need of noble-metal-based photocatalysts, excess additives and limited substrate scopes (Scheme 1a).

With the rapid development of sustainable chemistry, developing low-cost and transition-metal-free photocatalytic methods has become a strategic priority. In 2019 [54], the groups of Fu and Shang pioneered the photocatalytic decarboxylative alkylation of silyl enol ethers and *N*-heteroarenes by using a novel catalytic system based on sodium iodide (NaI) and triphenylphosphine (PPh_3_), suggested to function as an electron donor–acceptor (EDA) complex [55–60]. Compared to previously reported radical reactions, this novel catalytic system has the key advantage of circumventing the need for external redox additives and/or noble metals, using readily available and cost-effective NaI and PPh_3_ under mild reaction conditions. In a broader context, phosphine organocatalysis is probably still underappreciated in organic synthesis, and could lead to important future synthetic developments [61–67]. The NaI/PPh_3_ system has been further broadly applied to the functionalization of alkenes [68–70], as well as to decarboxylative C(sp^3^)–X bond formation [71], cyclization of 1,7-enynes [72–73] and other reactions [74–77]. Inspired by these advances, we developed here a visible light-mediated decarboxylative radical cascade cyclization of *N*-arylacrylamides under NaI/PPh_3_ catalysis, for the most efficient and practical synthesis of quaternary oxindoles (Scheme 1b and 1c). It should be noted that during the finalization of this work, a similar, however stoichiometric CsI/PPh_2_Cy-mediated method appeared from the Yang and Li groups (Scheme 1b) [28]. In contrast, the method we present here is 1) catalytic, 2) it employs the far less onerous NaI/PPh_3_ system, and 3) it displays a considerably broader substrate scope.

## Results and Discussion

Key elements of reaction optimization are summarized in Table 1. With NaI (20 mol %) and PPh_3_ (20 mol %), acrylamide **1a** and redox-active ester **2a** were used as model substrates to react for 36 h in acetonitrile (MeCN) under blue LEDs irradiation and N_2_ atmosphere, delivering the desired oxindole derivative **3aa** with 72% isolated yield (Table 1, entry 1). Other iodide sources, such as LiI, KI, RbI, CsI, CaI_2_, and a quaternary ammonium iodide, while also effective, provided slightly lower yields (Table 1, entries 2–7). It should be noted that all tested iodide sources were found soluble under those conditions. Some diverse phosphines were then screened. Aromatic phosphines performed best (Table 1, entries 8 and 9), the cheapest PPh_3_ remaining however optimal. In contrast, tricyclohexylphosphine PCy_3_ performed poorly (Table 1, entry 10), and bulky tri-*o*-tolylphosphine almost shut down the reaction (Table 1, entry 11). These results indicate that the accessibility of the phosphorus center is important. Next, the solvent was investigated. Replacing acetonitrile with dimethyl sulfoxide (DMSO), or dimethylacetamide (DMA), or acetone, or ethyl acetate (EA), resulted in inferior yields (Table 1, entries 12–15), and no product was detected when using 1,4-dioxane or dichloromethane (DCM) as reaction solvent (Table 1, entries 16 and 17). Although the reaction also proceeded without NaI, only a low yield of **3aa** was then obtained (Table 1, entry 18). PPh_3_ and irradiation are however both essential for this decarboxylative cascade cyclization process (Table 1, entries 19 and 20).

**Table 1:** Optimization table^a^.

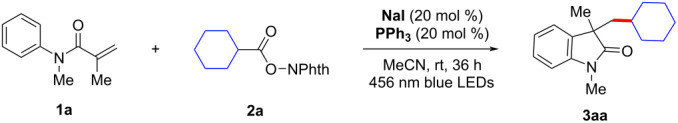		

Entry	Variation from standard conditions	**3aa**, Yield (%)^b^

1	none	76 (72)^c^
2	LiI instead of NaI	70
3	KI instead of NaI	62
4	RbI instead of NaI	64
5	CsI instead of NaI	39
6	CaI_2_ instead of NaI	56
7	*n-*Bu_4_NI instead of NaI	57
8	P(4-F-C_6_H_4_)_3_ instead of PPh_3_	73
9	P(4-OMe-C_6_H_4_)_3_ instead of PPh_3_	60
10	PCy_3_ instead of PPh_3_	23
11	P(2-Me-C_6_H_4_)_3_ instead of PPh_3_	trace
12	DMSO instead of MeCN	60
13	DMA instead of MeCN	44
14	acetone instead of MeCN	52
15	EA instead of MeCN	57
16	DCM instead of MeCN	nr
17	1,4-dioxane instead MeCN	nr
18	without NaI	14
19	without PPh_3_	0
20	without blue LED	0

^a^Unless otherwise noted, the standard reaction conditions were as follows: **1a** (0.3 mmol), **2a** (0.2 mmol), solvent (2 mL); ^b^the yield was determined by ^1^H NMR analysis of the crude reaction mixture using 1,3,5-trimethoxybenzene as an internal standard; ^c^isolated yield.

**Scheme 2:**
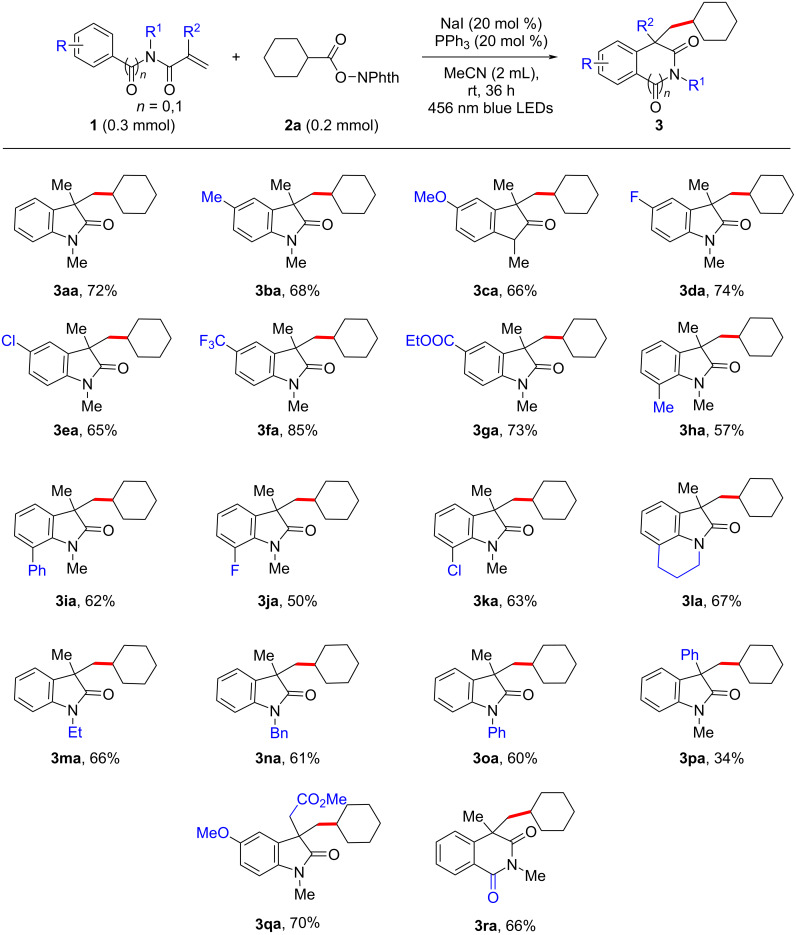
Arylamide substrate scope with isolated yields of products.

With the optimized conditions in hand, we then explored the scope of *N*-arylacrylamides with different substituents. A series of acrylamides showed good compatibility under standard conditions, offering the desired oxindoles in moderate to good yields (Scheme 2). Electron-donating groups at the *para*-position of the phenyl ring, such as methyl or methoxy groups, decreased slightly the yield, to 68% and 66%, respectively (**3ba** and **3ca**). When these substituents were replaced by common halogens or electron-withdrawing groups, good yields of the corresponding oxindoles (**3da**–**ga**) were achieved. A trifluoromethyl-substituted acrylamide afforded the product **3fa** in very high 85% yield. In addition, *ortho*-substitution at the *N*-aryl moiety was also well tolerated, albeit with slightly decreased yields (**3ha**–**ka**, 50–63%).

Interestingly, a cyclic *N*-arylamide derivative was also well tolerated, furnishing polycyclic structure **3la** in 67% yield. In addition, substrates with different *N-*substituents, such as ethyl, benzyl, and phenyl, could be converted into the expected products **3ma**–**oa** in good yields. It should be noted that replacing the methyl with a phenyl group at the *N*-arylacrylamide core significantly affected the reaction efficiency from 72% to 34% yield (**3pa**). Satisfyingly, substrate **1q** could successfully undergo decarboxylative cascade cyclization to afford **3qa** with 70% yield, which is used as a key intermediate in the synthesis of (±)-physovenine and (±)-physostigmine alkyl analogues exhibiting inhibitory activity against acetylcholinesterase and butyrylcholinesterase [30,78–84]. Subsequently, we expanded the scope of this protocol to include a benzamide derived acrylamide **1r**. The expected six-membered ring structure **3ra** could be successfully isolated with a good yield (66%).

**Scheme 3:**
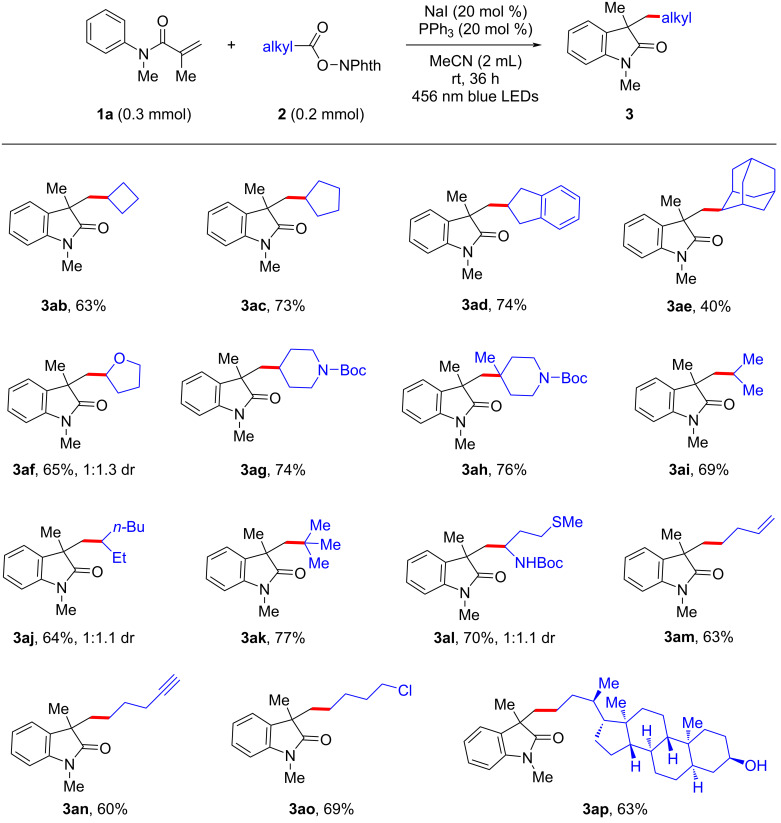
Alkyl radical precursor scope with isolated yields of products.

A number of alkyl radical precursors were then synthesized and evaluated in the reaction (Scheme 3). We found that redox-active esters derived from primary, secondary, and tertiary aliphatic carboxylic acids were all compatible with the method. Cyclic substrates bearing cyclobutyl, cyclopentyl, and indenyl groups could deliver the corresponding desired products with good yields (**3ab**–**ad**, 63–74%), while an adamantyl-derived substituent proved more challenging (**3ae**, 40%). The use of other cyclic substituents such as oxygen-containing and nitrogen-containing rings gave good yields of the target oxindoles (**3af**–**ah**, 65–76%). In addition, a symmetrically α-substituted redox-active esters furnished the corresponding quaternary oxindole **3ai** with 69% yield. Moreover, an asymmetrically α-branched starting material could react with similar efficiency, affording oxindole **3aj** as a 1:1.1 mixture of diastereomers. Interestingly, this method also enabled the synthesis of the highly sterically demanding oxindole **3ak** in good yield when using a *tert*-butyl *N*-hydroxyphthalimide ester as the *tert*-butyl radical precursor. Importantly, a redox-active ester derived from methionine could be converted effectively to α-aminoalkylation product **3al** in overall 70% yield, which thus provides a mild method for the functionalization and derivation of abundant natural or unnatural amino acids. Some functional groups such as a terminal alkene in **3am**, a terminal alkyne in **3an**, and an alkyl chloride in **3ao** proved compatible, associated with encouraging yields. In order to further demonstrate the utility of our protocol, a complex scaffold derived from lithocholic acid was tested, and was found to smoothly undergo the decarboxylative cyclization towards oxindole **3ap** in 63% yield.

**Scheme 4:**
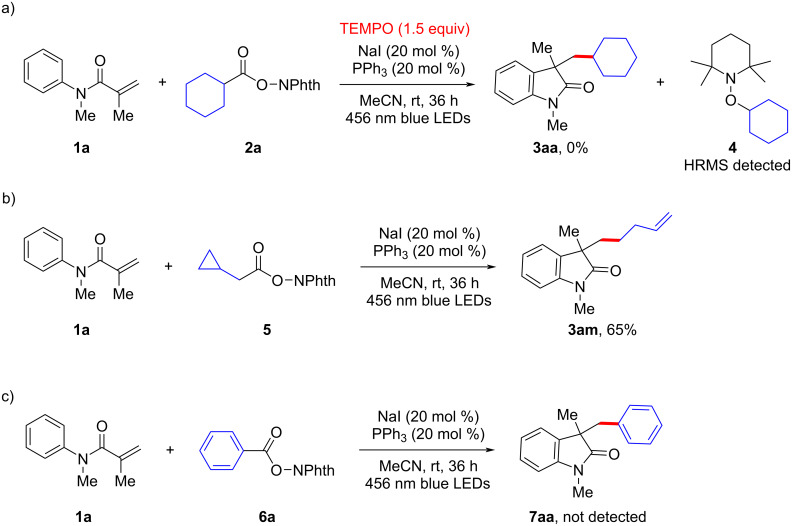
Selected mechanistic experiments.

In order to gain insight into the reaction mechanism, some control experiments were further performed. When a radical scavenger such as 2,2,6,6-tetramethyl-1-piperidinyloxyl (TEMPO) was added to the catalytic system under standard conditions, the reaction was fully inhibited, and a TEMPO-trapped adduct (**4**) was detected by HRMS (Scheme 4a). Moreover, the radical-mediated ring-opening product **3am** could be obtained with 66% yield in a radical clock experiment when redox-active ester **5** was engaged to react with acrylamide **1a** under standard conditions (Scheme 4b). Finally, it should be noted that benzoyl ester substrate **6a** did not deliver the corresponding cyclized product **7aa** (Scheme 4c). All of these outcomes indicate that a radical species should be involved in this decarboxylative cascade cyclization towards oxindoles under NaI/PPh_3_ catalysis. Thus, the mechanism should run in a similar fashion to related well-documented previous reports [54,68–77], through a light-induced, phosphine-assisted, intermolecular electron transfer from sodium iodide to the redox-active ester.

## Conclusion

In summary, we developed an effective photocatalytic decarboxylative radical cascade cyclization of *N*-arylacrylamides with various redox-active esters derived from common and/or important carboxylic acids under mild conditions. Complementary to traditional transition metal photocatalysis and organo-photocatalysis [85], the readily available and inexpensive NaI/PPh_3_ can operate as an efficient photoredox catalyst, providing an economical access to construct important oxindole scaffolds containing a quaternary carbon center. This synthetic method features a broad substrate scope, good functional group tolerance and operational simplicity. Mechanistic investigations revealed that this cyclization reaction proceeds via a cascade radical pathway. We expect these results to encourage the further development of NaI/PPh_3_-catalyzed and related synthetic methods.

## Supporting Information

File 1 Experimental section and characterization of synthesized compounds.
