# Supplementary material for: NaI/PPh3-catalyzed visible-light-mediated decarboxylative radical cascade cyclization of N-arylacrylamides for the efficient synthesis of quaternary oxindoles
Source: Beilstein J Org Chem. 2023 Jan 16;19:57–65. doi: 10.3762/bjoc.19.5 (PMC9874234; doi:10.3762/bjoc.19.5)

BEILSTEIN JOURNAL OF ORGANIC CHEMISTRY

Supporting Information

for

NaI/PPh$_3$-catalyzed visible-light-mediated decarboxylative radical cascade cyclization of *N*-arylacrylamides for the efficient synthesis of quaternary oxindoles

Dan Liu, Yue Zhao and Frederic W. Patureau

Beilstein J. Org. Chem. **2023,** *19,* 57–65. doi:10.3762/bjoc.19.5

Experimental section and characterization of synthesized compounds

License and Terms: This is a supporting information file under the terms of the Creative Commons Attribution License (https://creativecommons.org/licenses/by/4.0). Please note that the reuse, redistribution and reproduction in particular requires that the author(s) and source are credited and that individual graphics may be subject to special legal provisions.
The license is subject to the *Beilstein Journal of Organic Chemistry* terms and conditions: (https://www.beilstein-journals.org/bjoc/terms)

Table of contents

1. General..S2
2. General procedure for the synthesis of quaternary oxindoles.................................S3
3. Product characterization...S4
4. Mechanism studies..S18
5. References...S19
6. NMR Spectra of products S20

1. General

^{1}H NMR, ^{13}C NMR, and ^{19}F NMR spectra were recorded on an Agilent VNMRS 400 or a Bruker Av 600 using $CDCl_3$ as solvent. Chemical shifts are given in ppm and coupling constants (*J*) in Hz. ^{1}H NMR spectra were calibrated in relation to the reference measurement of TMS (0.00 ppm). ^{13}C NMR spectra were calibrated in relation to the deuterated solvent, namely $CDCl_3$ (77.16 ppm). The following abbreviations were used for ^{1}H NMR spectra to indicate the signal multiplicity: s (singlet), d (doublet), t (triplet), q (quartet) and m (multiplet) as well as combinations of them. Flash chromatography was performed on silica gel (60 M, 0.04–0.063 mm) by standard techniques. All the chemicals used for synthesis were purchased from Sigma Aldrich, abcr, Alfa Aesar, TCI, Fisher, or chemPUR. High resolution mass spectra (HRMS) were obtained on a Thermo Scientific LTQ Orbitrap XL spectrometer. IR spectra were measured on a PerkinElmer 100 FT-IR spectrometer with an UATR Diamond KRS-5 unit. Acrylamide substrates[1-2] and *N*-hydroxyphthalimide esters[3] were prepared according to literature procedures.

All reactions with blue LEDs irradiation were carried out using a photoreactor equipped with LED PhotoReaction Lighting (Model: PR160L, λ = 456 nm, max 40 W), which was bought from Kessil company. The vials (15 mL) were placed at approximatively 4 cm away from the light source. In order to avoid overheating of the reaction system, the vials were cooled with a fan on top of the vials.

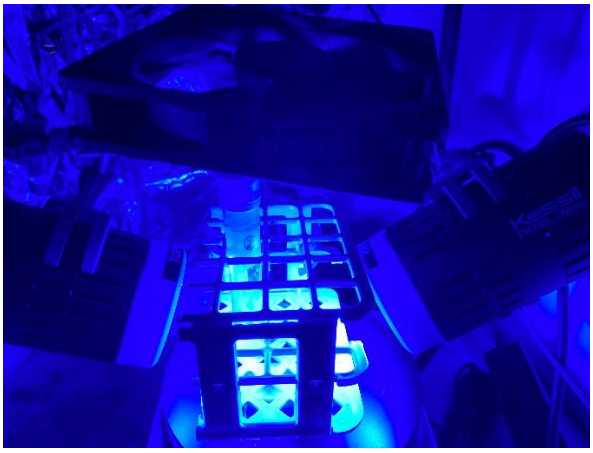

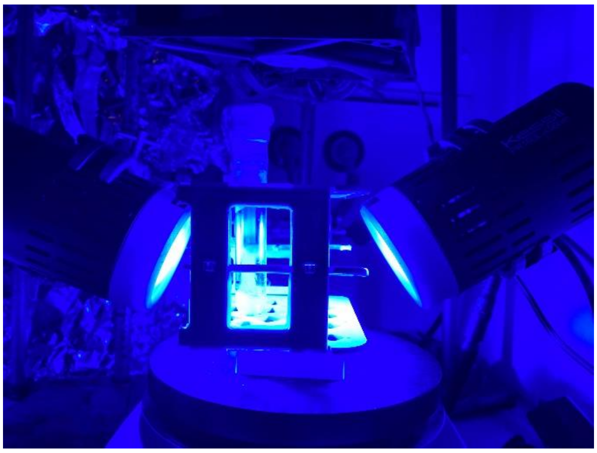

2. General procedure for the synthesis of quaternary oxindoles

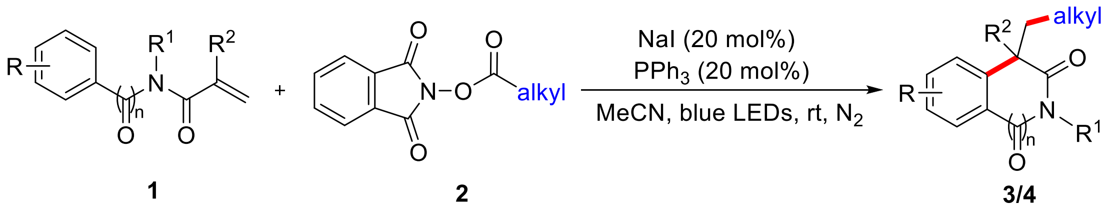

Procedure: To an oven vial equipped with a stirring bar, *N*-arylarylamide or *N*-alkylarylamide **1** (0.3 mmol), alkyl redox-active ester **2** (0.2 mmol), NaI (6 mg, 0.04 mmol) and PPh_3 (10.5 mg, 0.04 mmol) were added, then the tube was evacuated and filled with N_2 (three times) before anhydrous acetonitrile (MeCN, 2.0 mL) was added. The reaction was performed under blue LEDs irradiation (456 nm, 40 W) at room temperature. After 36 h, the solvent was removed in vacuo, and the residue was purified by column chromatography to give the corresponding quaternary oxindole products.

3. Product characterization

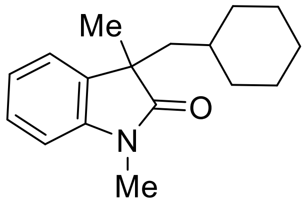

3-(Cyclohexylmethyl)-1,3-dimethylindolin-2-one (3aa)[4]

The crude mixture was purified by silica gel column chromatography with pentane/EA (10:1). 37 mg product was obtained by 72% isolated yield as colorless liquid.

^{1}H NMR (600 MHz, $CDCl_3$) δ 7.18 (t, *J* = 7.8 Hz, 1H), 7.08 (d, *J* = 7.2 Hz, 1H), 6.98 (t, *J* = 7.2 Hz, 1H), 6.76 (d, *J* = 7.8 Hz, 1H), 3.14 (s, 3H), 1.85 (dd, *J* = 14.4, 6.6 Hz, 1H), 1.65 (dd, *J* = 14.4, 5.4 Hz, 1H), 1.47 – 1.34 (m, 3H), 1.27 (d, *J* = 11.4 Hz, 1H), 1.24 (s, 3H), 1.14 (d, *J* = 12.0 Hz, 1H), 0.98 – 0.82 (m, 4H), 0.78 – 0.64 (m, 2H).

^{13}C NMR (151 MHz, $CDCl_3$) δ 181.1, 143.1, 134.4, 127.5, 122.7, 122.3, 107.9, 47.8, 45.4, 34.7, 34.5, 33.5, 26.2, 26.15, 26.10, 26.0.

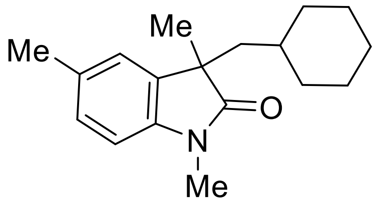

3-(Cyclohexylmethyl)-1,3,5-trimethylindolin-2-one (3ba)[4]

The crude mixture was purified by silica gel column chromatography with pentane/EA (10:1). 37 mg product was obtained by 68% isolated yield as colorless liquid.

^{1}H NMR (600 MHz, Chloroform-*d*) δ 6.98 (d, *J* = 7.8 Hz, 1H), 6.89 (s, 1H), 6.65 (d, *J* = 7.8 Hz, 1H), 3.12 (s, 3H), 2.28 (s, 3H), 1.84 (dd, *J* = 14.4, 7.2 Hz, 1H), 1.63 (dd, *J* = 14.4, 5.4 Hz, 1H), 1.48 – 1.36 (m, 3H), 1.30 – 1.12 (m, 5H), 0.94 – 0.86 (m, 4H), 0.78 – 0.64 (m, 2H).

^{13}C NMR (151 MHz, Chloroform-*d*) δ 181.1, 140.8, 134.5, 131.8, 127.7, 123.6, 107.6, 47.9, 45.4, 34.7, 34.5, 33.5, 26.3, 26.2, 26.1, 26.0, 21.2.

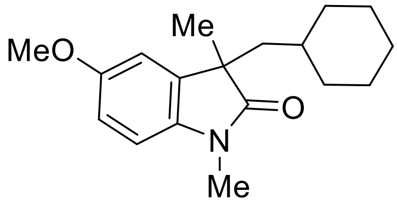

1-(Cyclohexylmethyl)-6-methoxy-1,3-dimethyl-1,3-dihydro-2*H*-inden-2-one (3ca)[4]

The crude mixture was purified by silica gel column chromatography with pentane/EA (8:1). 38 mg product was obtained by 66% isolated yield as colorless liquid.

^{1}H NMR (600 MHz, Chloroform-*d*) δ 6.73 – 6.69 (m, 2H), 6.68 – 6.65 (m, 1H), 3.74 (s, 3H), 3.12 (s, 3H), 1.85 (dd, *J* = 14.4, 7.2 Hz, 1H), 1.62 (dd, *J* = 14.4, 5.4 Hz, 1H), 1.47 – 1.36 (m, 3H), 1.38 – 1.12 (m, 5H), 0.94 – 0.82 (m, 4H), 0.82 – 0.63 (m, 2H).
^{13}C NMR (151 MHz, Chloroform-*d*) δ 180.8, 155.9, 136.7, 135.9, 111.4, 110.5, 108.1, 55.8, 48.3, 45.4, 34.7, 34.4, 33.5, 26.6, 26.3, 26.1, 26.0.

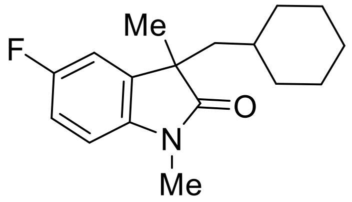

3-(Cyclohexylmethyl)-5-fluoro-1,3-dimethylindolin-2-one (3da)[5]
The crude mixture was purified by silica gel column chromatography with pentane/EA (20:1). 41 mg product was obtained by 74% isolated yield as colorless liquid.
^{1}H NMR (400 MHz, Chloroform-*d*) δ 7.01 – 6.85 (m, 2H), 6.74 (dd, *J* = 8.4, 4.0 Hz, 1H), 3.19 (s, 3H), 1.92 (dd, *J* = 14.2, 6.8 Hz, 1H), 1.68 (dd, *J* = 14.2, 5.2 Hz, 1H), 1.55 – 1.42 (m, 3H), 1.34 – 1.18 (m, 5H), 1.02 – 0.88 (m, 4H), 0.85 – 0.71 (m, 2H).
^{13}C NMR (101 MHz, Chloroform-d) δ 180.7, 159.3 (d, *J* = 242.4 Hz), 139.0 (d, *J* = 1.6 Hz), 136.2 (d, *J* = 8.1 Hz), 113.6 (d, *J* = 23.2 Hz), 111.8 (d, *J* = 25.2 Hz), 108.3 (d, *J* = 8.1 Hz), 48.3 (d, *J* = 1.6 Hz), 45.3, 34.7, 34.4, 33.4, 26.3, 26.1, 26.04, 26.01, 25.98.
^{19}F NMR (376 MHz, Chloroform-*d*) δ -121.02 (td, *J* = 8.7, 3.9 Hz).

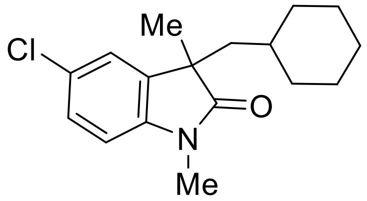

5-Chloro-3-(cyclohexylmethyl)-1,3-dimethylindolin-2-one (3ea)[5]
The crude mixture was purified by silica gel column chromatography with pentane/EA (10:1). 38 mg product was obtained by 65% isolated yield as colorless liquid.
^{1}H NMR (600 MHz, Chloroform-*d*) δ 7.16 (dd, *J* = 8.4, 1.2 Hz, 1H), 7.06 (d, *J* = 2.4 Hz, 1H), 6.69 (d, *J* = 8.4 Hz, 1H), 3.13 (s, 3H), 1.86 (dd, *J* = 14.4, 7.2 Hz, 1H), 1.63 (dd, *J* = 14.4, 5.4 Hz, 1H), 1.49 – 1.34 (m, 3H), 1.26 – 1.12 (m, 5H), 0.98 – 0.83 (m, 4H), 0.79 – 0.63 (m, 2H).
^{13}C NMR (151 MHz, Chloroform-d) δ 180.6, 141.7, 136.2, 127.8, 127.5, 123.2, 108.9, 48.1, 45.3, 34.7, 34.4, 33.4, 26.3, 26.2, 26.1, 26.03, 25.98.

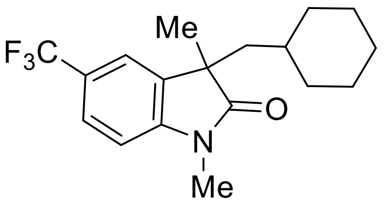

3-(Cyclohexylmethyl)-1,3-dimethyl-5-(trifluoromethyl)indolin-2-one (3fa)[4]
The crude mixture was purified by silica gel column chromatography with pentane/EA (10:1). 55 mg product was obtained by 85% isolated yield as colorless liquid.
^{1}H NMR (600 MHz, Chloroform-*d*) δ 7.48 (d, *J* = 7.8 Hz, 1H), 7.31 (s, 1H), 6.84 (d, *J* = 7.8 Hz, 1H), 3.18 (s, 3H), 1.88 (dd, *J* = 14.4, 7.2 Hz, 1H), 1.68 (dd, *J* = 14.4, 5.4 Hz, 1H), 1.48 – 1.36 (m, 3H), 1.28 – 1.09 (m, 5H), 0.96 – 0.82 (m, 4H), 0.80 – 0.64 (m, 2H).
^{13}C NMR (151 MHz, Chloroform-*d*) δ 181.0, 146.1, 135.0, 125.4 (q, *J* = 4.7 Hz), 124.6 (q, *J* = 33.5 Hz), 119.7 (q, *J* = 4.4 Hz, 1H), 107.7, 47.9, 45.3, 34.7, 34.4, 33.5, 26.4, 26.0, 25.98, 25.94.
^{19}F NMR (282 MHz, Chloroform-*d*) δ -61.32.

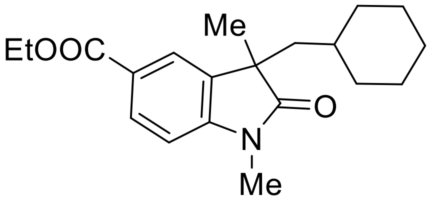

Ethyl 3-(cyclohexylmethyl)-1,3-dimethyl-2-oxoindoline-5-carboxylate (3ga)[4]
The crude mixture was purified by silica gel column chromatography with pentane/EA (10:1). 48 mg product was obtained by 73% isolated yield as colorless liquid.
^{1}H NMR (600 MHz, Chloroform-d) δ 7.96 (d, *J* = 8.4 Hz, 1H), 7.76 (s, 1H), 6.80 (d, *J* = 8.4 Hz, 1H), 4.31 (q, *J* = 7.2 Hz, 2H), 3.18 (s, 3H), 1.87 (dd, *J* = 14.4, 7.2 Hz, 1H), 1.75 – 1.68 (dd, *J* = 14.4, 7.2 Hz, 1H), 1.48 – 1.37 (m, 3H), 1.34 (t, *J* = 7.2 Hz, 3H), 1.29 – 1.11 (m, 5H), 0.97 – 0.79 (m, 4H), 0.79 – 0.63 (m, 2H).
^{13}C NMR (151 MHz, Chloroform-*d*) δ 181.4, 166.6, 147.2, 134.3, 130.3, 124.7, 123.9, 107.4, 60.9, 47.7, 45.3, 34.7, 34.4, 33.4, 26.4, 26.1, 26.0, 25.98, 14.4.

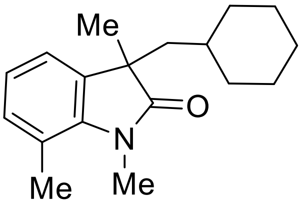

3-(Cyclohexylmethyl)-1,3,7-trimethylindolin-2-one (3ha)[4]
The crude mixture was purified by silica gel column chromatography with pentane/EA (10:1). 31 mg product was obtained by 57% isolated yield as colorless liquid.
^{1}H NMR (600 MHz, Chloroform-*d*) δ 6.99 – 6.64 (m, 3H), 3.42 (s, 3H), 2.52 (s, 3H), 1.84 (dd, *J* = 14.4, 7.2 Hz, 1H), 1.61 (dd, *J* = 14.4, 5.4 Hz, 1H), 1.48 – 1.37 (m, 3H), 1.29 (d, *J* = 13.2 Hz, 1H), 1.21 (s, 3H), 1.15 (d, *J* = 13.2 Hz, 1H), 0.96 – 0.83 (m, 4H), 0.79 – 0.62 (m, 2H).

^{13}C NMR (151 MHz, Chloroform-*d*) δ 181.9, 140.9, 135.1, 131.2, 122.2, 120.7, 119.5, 47.2, 45.7, 34.6, 34.5, 33.5, 29.5, 26.6, 26.1, 26.1, 26.0, 19.1.

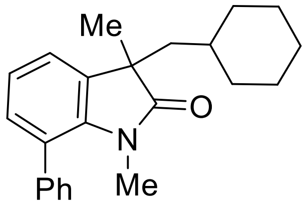

3-(Cyclohexylmethyl)-1,3-dimethyl-7-phenylindolin-2-one (3ia)[4]

The crude mixture was purified by silica gel column chromatography with pentane/EA (10:1). 41 mg product was obtained by 62% isolated yield as colorless liquid.

^{1}H NMR (600 MHz, Chloroform-*d*) δ 7.41 – 7.20 (m, 5H), 7.07 (d, *J* = 7.2 Hz, 1H), 7.04 – 6.91 (m, 2H), 2.65 (s, 3H), 1.88 (dd, *J* = 13.8, 7.2 Hz, 1H), 1.67 (dd, *J* = 13.8, 5.4 Hz, 1H), 1.56 – 1.37 (m, 3H), 1.33 (d, *J* = 12.0 Hz, 1H), 1.29 (s, 3H), 1.17 (d, *J* = 14.2 Hz, 1H), 0.98 – 0.89 (m, 4H), 0.80 – 0.67 (m, 2H).

^{13}C NMR (151 MHz, Chloroform-*d*) δ 182.2, 140.1, 139.2, 135.5, 130.5, 130.0, 129.8, 127.9, 127.8, 127.6, 125.4, 121.7, 121.6, 47.2, 46.0, 34.8, 34.5, 33.6, 30.2, 26.3, 26.2, 26.14, 26.1.

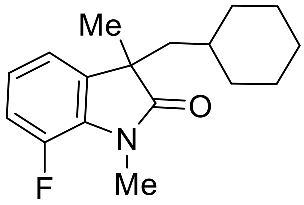

3-(Cyclohexylmethyl)-7-fluoro-1,3-dimethylindolin-2-one (3ja)[4]

The crude mixture was purified by silica gel column chromatography with pentane/EA (10:1). 28 mg product was obtained by 50% isolated yield as colorless liquid.

^{1}H NMR (600 MHz, Chloroform-*d*) δ 6.94 – 6.82 (m, 3H), 3.36 (s, 3H), 1.86 (dd, *J* = 14.4, 7.2 Hz, 1H), 1.64 (dd, *J* = 14.4, 5.4 Hz, 1H), 1.49 – 1.36 (m, 3H), 1.28 (d, *J* = 12.0 Hz, 1H), 1.24 (s, 3H), 1.14 (d, *J* = 12.0 Hz, 1H), 0.95 – 0.83 (m, 4H), 0.81 – 0.65 (m, 2H).

^{13}C NMR (151 MHz, Chloroform-*d*) δ 180.7, 147.8 (d, *J* = 243.4 Hz), 137.5 (d, *J* = 3.0 Hz), 129.7 (d, *J* = 8.0 Hz), 122.9 (d, *J* = 6.3 Hz), 118.6 (d, *J* = 3.2 Hz) 115.6 (d, *J* = 19.3 Hz), 48.2 (d, *J* = 2.0 Hz), 45.6, 34.7, 34.4, 33.5, 28.7, 28.6, 26.4, 26.08, 26.06, 26.0.

^{19}F NMR (565 MHz, Chloroform-d) δ -136.73.

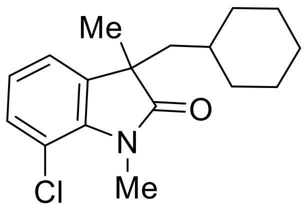

7-Chloro-3-(cyclohexylmethyl)-1,3-dimethylindolin-2-one (3ka)[5]

The crude mixture was purified by silica gel column chromatography with pentane/EA (10:1). 37 mg product was obtained by 63% isolated yield as colorless liquid.

^{1}H NMR (600 MHz, Chloroform-*d*) δ 7.10 (d, *J* = 8.4 Hz, 1H), 6.96 (d, *J* = 7.2 Hz, 1H), 6.89 (t, *J* = 7.8 Hz, 1H), 3.51 (s, 3H), 1.86 (dd, *J* = 14.4, 7.2 Hz, 1H), 1.62 (dd, *J* = 14.4, 5.4 Hz, 1H), 1.49 – 1.37 (m, 3H), 1.27 (d, *J* = 13.8 Hz, 1H), 1.23 (s, 3H), 1.14 (d, *J* = 12.6 Hz, 1H), 0.95 – 0.82 (m, 4H), 0.78 – 0.65 (m, 2H).

^{13}C NMR (151 MHz, Chloroform-*d*) δ 181.3, 139.0, 137.3, 129.9, 123.1, 121.2, 115.4, 47.7, 45.6, 34.6, 34.5, 33.5, 29.5, 26.6, 26.1, 26.0.

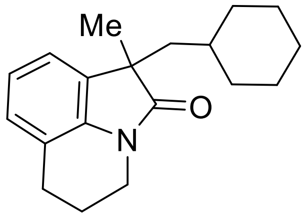

1-(Cyclohexylmethyl)-1-methyl-5,6-dihydro-4*H*-pyrrolo[3,2,1-ij]quinolin-2(1*H*)-one (3la)

The crude mixture was purified by silica gel column chromatography with pentane/EA (10:1). 38 mg product was obtained by 67% isolated yield as colorless liquid.

^{1}H NMR (600 MHz, Chloroform-*d*) δ 6.98 – 6.83 (m, 3H), 3.64 (t, *J* = 4.8 Hz, 2H), 2.76 – 2.68 (m, 2H), 1.96 – 1.86 (m, 2H), 1.83 (dd, *J* = 14.4, 7.2 Hz, 1H), 1.64 (dd, *J* = 14.4, 4.2 Hz, 1H), 1.47 – 1.37 (m, 3H), 1.32 (d, *J* = 13.2 Hz, 1H), 1.18 (d, *J* = 14.4 Hz, 1H), 1.25 (s, 3H), 0.96 – 0.88 (m, 4H), 0.80 – 0.67 (m, 2H).

^{13}C NMR (151 MHz, Chloroform-*d*) δ 180.0, 138.9, 133.0, 126.3, 121.8, 120.6, 120.0, 49.2, 45.3, 38.8, 34.8, 34.5, 33.6, 26.15, 26.13, 26.0, 25.8, 24.7, 21.4.

HRMS (ESI-MS) Calcd. For $C_{19}H_{25}NONa$ $[M+Na]^+$ 306.18284, found: 306.18234.

IR (neat, cm^{-1}): ṽ: 3413, 2921, 1706, 1626, 1482, 1347, 1238, 1164, 1065, 956, 871, 749.

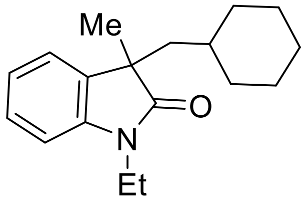

3-(Cyclohexylmethyl)-1-ethyl-3-methylinolin-2-one (3ma)[4]

The crude mixture was purified by silica gel column chromatography with pentane/EA (10:1). 36 mg product was obtained by 66% isolated yield as colorless liquid.

^{1}H NMR (600 MHz, Chloroform-*d*) δ 7.17 (td, *J* = 7.2, 1.2 Hz, 1H), 7.09 (dd, *J* = 7.8, 1.2 Hz, 1H), 6.97 (td, *J* = 7.2, 1.2 Hz, 1H), 6.78 (d, *J* = 7.8 Hz, 1H), 3.81 (dq, *J* = 14.4,

7.3 Hz, 1H), 3.83-3.78 (dq, J = 14.4, 7.2 Hz, 1H), 1.85 (dd, J = 14.4, 6.6 Hz, 1H), 1.64 (dd, J = 14.4, 6.6 Hz, 1H), 1.47 – 1.31 (m, 4H), 1.23 (s, 3H), 1.17 (t, J = 7.2 Hz, 3H), 1.12 – 1.07 (m, 1H), 0.95 – 0.82 (m, 4H), 0.79 – 0.61 (m, 2H).
^{13}C NMR (151 MHz, Chloroform-*d*) δ 180.6, 142.2, 134.8, 127.4, 122.9, 122.1, 108.1, 47.7, 45.5, 34.8, 34.4, 34.38, 33.7, 26.1, 26.08, 26.0, 12.5.

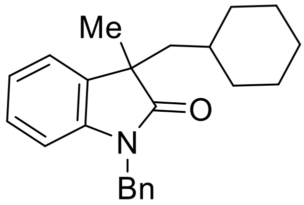

1-Benzyl-3-(cyclohexylmethyl)-3-methylindolin-2-one (3na)[4]
The crude mixture was purified by silica gel column chromatography with pentane/EA (10:1). 41 mg product was obtained by 61% isolated yield as colorless liquid.
^{1}H NMR (600 MHz, Chloroform-*d*) δ 7.23 – 7.13 (m, 5H), 7.10 – 7.04 (m, 2H), 6.93 (t, J = 7.2 Hz, 1H), 6.66 (d, J = 7.8 Hz, 1H), 4.97 (d, J = 15.6 Hz, 1H), 4.72 (d, J = 15.6 Hz, 1H), 1.91 (dd, J = 13.8, 6.6 Hz, 1H), 1.68 (dd, J = 13.8, 6.0 Hz, 1H), 1.45 – 1.34 (m, 4H), 1.29 (s, 3H), 1.07 (d, J = 12.6 Hz, 1H), 0.96 – 0.74 (m, 5H), 0.67 – 0.59 (m, 1H).
^{13}C NMR (151 MHz, Chloroform-d) δ 181.0, 142.3, 136.2, 134.6, 128.7, 127.5, 127.4, 122.8, 122.3, 109.0, 47.9, 45.5, 43.7, 34.8, 34.4, 34.0, 26.6, 26.12, 26.10, 26.06.

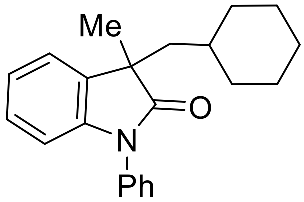

3-(Cyclohexylmethyl)-3-methyl-1-phenylindolin-2-one (3oa)[4]
The crude mixture was purified by silica gel column chromatography with pentane/EA (10:1). 38 mg product was obtained by 60% isolated yield as colorless liquid.
^{1}H NMR (600 MHz, Chloroform-*d*) δ 7.49 – 7.41 (m, 2H), 7.35 – 7.28 (m, 3H), 7.16 – 7.09 (m, 2H), 7.02 (t, J = 7.2 Hz, 1H), 6.76 (d, J = 7.8 Hz, 1H), 1.96 (dd, J = 13.8, 7.2 Hz, 1H), 1.73 (dd, J = 13.8, 5.4 Hz, 1H), 1.50 – 1.39 (m, 4H), 1.37 (s, 3H), 1.23 – 1.19 (m, 1H), 1.05 – 0.89 (m, 4H), 0.84 – 0.71 (m, 2H).
^{13}C NMR (151 MHz, Chloroform-*d*) δ 180.5, 143.0, 134.8, 134.2, 129.6, 127.8, 127.4, 126.5, 123.0, 122.8, 109.3, 47.9, 45.9, 35.0, 34.4, 33.6, 26.5, 26.2, 26.1, 26.08.

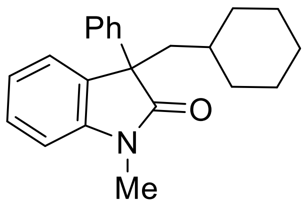

3-(Cyclohexylmethyl)-1-methyl-3-phenylindolin-2-one (3pa)[4]
The crude mixture was purified by silica gel column chromatography with pentane/EA (10:1). 22 mg product was obtained by 34% isolated yield as colorless liquid.
^{1}H NMR (600 MHz, Chloroform-*d*) δ 7.32 – 7.24 (m, 3H), 7.21 – 7.15 (m, 4H), 7.15 – 7.11 (m, 1H), 7.05 (t, *J* = 7.8 Hz, 1H), 6.83 (d, *J* = 7.8 Hz, 1H), 3.13 (s, 3H), 2.33 (dd, *J* = 13.8, 6.6 Hz, 1H), 2.07 (dd, *J* = 13.8, 5.4 Hz, 1H), 1.49 – 1.31 (m, 4H), 0.98 – 0.73 (m, 7H).
^{13}C NMR (151 MHz, Chloroform-*d*) δ 179.0, 143.9, 141.6, 132.1, 128.4, 128.1, 127.1, 126.7, 125.3, 122.4, 108.3, 56.1, 45.3, 34.9, 34.6, 33.6, 29.7, 26.5, 26.2, 26.1.

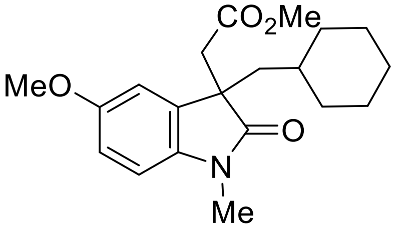

Methyl 2-(3-(cyclohexylmethyl)-5-methoxy-1-methyl-2-oxoindolin-3-yl)acetate (3qa)[5]
The crude mixture was purified by silica gel column chromatography with pentane/EA (10:1). 48 mg product was obtained by 70% isolated yield as colorless liquid.
^{1}H NMR (600 MHz, Chloroform-*d*) δ 6.75 – 6.63 (m, 3H), 3.72 (s, 3H), 3.36 (s, 3H), 3.15 (s, 3H), 2.87 (d, *J* = 16.2 Hz, 1H), 2.70 (d, *J* = 16.2 Hz, 1H), 1.76 (dd, *J* = 13.8, 6.0 Hz, 1H), 1.63 (dd, *J* = 13.8, 5.4 Hz, 1H), 1.48 – 1.33 (m, 4H), 1.09 (d, *J* = 11.4 Hz, 1H), 0.96 – 0.85 (m, 4H), 0.82 – 0.74 (m, 1H), 0.69 – 0.63 (m, 1H).
^{13}C NMR (151 MHz, Chloroform-*d*) δ 179.2, 170.1, 155.7, 138.0, 132.8, 112.0, 110.7, 108.1, 55.8, 51.5, 49.7, 45.2, 42.6, 34.6, 34.0, 33.9, 26.4, 26.1, 26.04, 26.02.

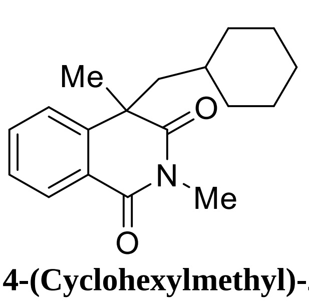

4-(Cyclohexylmethyl)-2,4-dimethylisoquinoline-1,3(2*H*,4*H*)-dione (3ra)[5]
The crude mixture was purified by silica gel column chromatography with pentane/EA (10:1). 38 mg product was obtained by 66% isolated yield as colorless liquid.
^{1}H NMR (600 MHz, Chloroform-*d*) δ 8.19 (d, *J* = 7.8 Hz, 1H), 7.56 (t, *J* = 7.8 Hz, 1H), 7.40 – 7.29 (m, 2H), 3.32 (s, 3H), 2.26 (dd, *J* = 14.4, 7.8 Hz, 1H), 1.83 (dd, *J* = 14.4, 4.8 Hz, 1H), 1.49 (s, 3H), 1.46 – 1.35 (m, 3H), 1.18 (d, *J* = 8.4 Hz, 1H), 1.08 (d, *J* = 12.6 Hz, 1H), 0.94 – 0.63 (m, 6H).

^{13}C NMR (151 MHz, Chloroform-*d*) δ 176.9, 164.5, 143.9, 133.7, 128.9, 127.2, 125.7, 124.6, 49.6, 46.7, 34.9, 34.3, 33.0, 31.7, 27.2, 26.03, 26.0, 26.96.

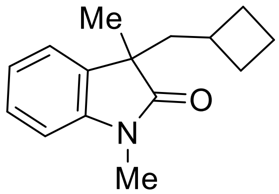

3-(Cyclobutylmethyl)-1,3-dimethylindolin-2-one (3ab)[6]

The crude mixture was purified by silica gel column chromatography with pentane/EA (10:1). 29 mg product was obtained by 63% isolated yield as colorless liquid.

^{1}H NMR (600 MHz, Chloroform-*d*) δ 7.18 (t, *J* = 7.8 Hz, 1H), 7.09 (d, *J* = 7.8 Hz, 1H), 6.97 (t, *J* = 7.8 Hz, 1H), 6.75 (d, *J* = 7.8 Hz, 1H), 3.12 (s, 3H), 1.97 (dd, *J* = 12.6, 6.6 Hz, 1H), 1.87 – 1.76 (m, 2H), 1.66 – 1.59 (m, 1H), 1.57 – 1.47 (m, 3H), 1.43 – 1.37 (m, 1H), 1.36 – 1.28 (m, 1H), 1.25 (s, 3H).

^{13}C NMR (151 MHz, Chloroform-*d*) δ 180.8, 143.3, 134.2, 127.6, 122.8, 122.2, 107.7, 47.9, 45.6, 32.9, 29.5, 28.9, 26.1, 23.9, 18.9.

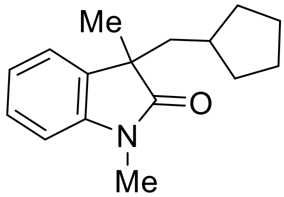

3-(Cyclopentylmethyl)-1,3-dimethylindolin-2-one (3ac)[6]

The crude mixture was purified by silica gel column chromatography with pentane/EA (10:1). 36 mg product was obtained by 73% isolated yield as colorless liquid.

^{1}H NMR (600 MHz, Chloroform-*d*) δ 7.19 (t, *J* = 7.8 Hz, 1H), 7.09 (d, *J* = 6.0 Hz, 1H), 6.98 (t, *J* = 7.8 Hz, 1H), 6.76 (d, *J* = 7.8 Hz, 1H), 3.15 (s, 3H), 1.99 (dd, *J* = 13.8, 7.2 Hz, 1H), 1.82 (dd, *J* = 13.8, 6.0 Hz, 1H), 1.43-1.31 (m, 3H), 1.27 (s, 3H), 1.25 – 1.11 (m, 4H), 0.97 – 0.90 (m, 1H), 0.80 – 0.70 (m, 1H).

^{13}C NMR (151 MHz, Chloroform-*d*) δ 181.2, 143.3, 134.4, 127.6, 122.9, 122.3, 107.9, 48.5, 44.5, 37.2, 33.8, 32.8, 26.2, 25.3, 24.9, 24.86.

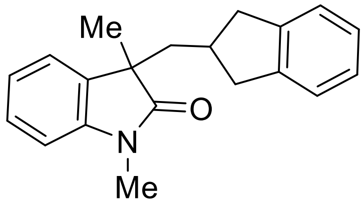

3-((2,3-Dihydro-1*H*-inden-2-yl)methyl)-1,3-dimethylindolin-2-one (3ad)[6]

The crude mixture was purified by silica gel column chromatography with pentane/EA (10:1). 43 mg product was obtained by 74% isolated yield as colorless liquid.

^{1}H NMR (600 MHz, Chloroform-*d*) δ 7.20 (t, *J* = 7.8 Hz, 1H), 7.13 (d, *J* = 7.8 Hz, 1H), 7.03 – 6.88 (m, 5H), 6.78 (d, *J* = 7.8 Hz, 1H), 3.17 (s, 3H), 2.59 (dd, *J* = 15.6, 7.6 Hz,

1H), 2.47 (dd, *J* = 15.6, 9.6 Hz, 1H), 2.34 (dd, *J* = 15.6, 7.8 Hz, 1H), 2.29 – 2.19 (m, 2H), 2.03 (dd, *J* = 13.8, 6.0 Hz, 1H), 1.98 – 1.92 (m, 1H), 1.32 (s, 3H).
^{13}C NMR (151 MHz, Chloroform-*d*) δ 180.8, 143.3, 143.2, 143.1, 134.0, 127.9, 126.1, 126.0, 124.1, 124.0, 122.9, 122.5, 108.0, 48.5, 43.9, 40.2, 39.5, 37.8, 26.3, 25.1.

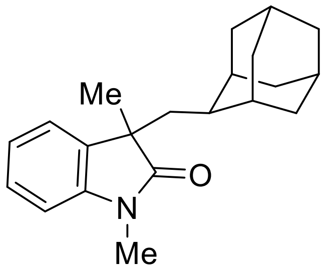

3-(((1*r*,3*s*,5*r*,7*r*)-Adamantan-2-yl)methyl)-1,3-dimethylindolin-2-one (3ae)[6]
The crude mixture was purified by silica gel column chromatography with pentane/EA (10:1). 25 mg product was obtained by 40% isolated yield as colorless liquid.
^{1}H NMR (600 MHz, Chloroform-*d*) δ 7.17 (t, *J* = 7.8 Hz, 1H), 7.09 (d, *J* = 7.2 Hz, 1H), 6.96 (t, *J* = 7.8 Hz, 1H), 6.75 (d, *J* = 7.8 Hz, 1H), 3.13 (s, 3H), 2.05 (dd, *J* = 13.8, 6.0 Hz, 1H), 1.82 – 1.70 (m, 3H), 1.66 – 1.49 (m, 6H), 1.42 – 1.29 (m, 5H), 1.28 (s, 3H), 1.21 – 1.17 (m, 1H), 1.08 – 1.05 (m, 1H).
^{13}C NMR (151 MHz, Chloroform-*d*) δ 180.9, 143.3, 134.3, 127.6, 122.6, 122.3, 107.8, 48.4, 41.7, 41.0, 39.0, 39.0, 38.1, 33.2, 32.6, 31.8, 31.7, 27.7, 27.6, 26.1, 25.1.

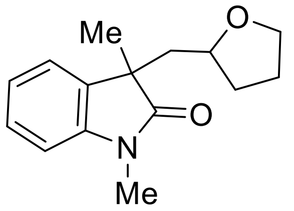

1,3-Dimethyl-3-((tetrahydrofuran-2-yl)methyl)indolin-2-one (3af)
The crude mixture was purified by silica gel column chromatography with pentane/EA (10:1). 32 mg (dr = 1:1.3) product was obtained by 65% isolated yield as colorless liquid.
^{1}H NMR (600 MHz, Chloroform-*d*) mixture of diastereomers δ 7.24 – 7.09 (m, 4H), 7.00 – 6.95 (m, 2H), 6.78 – 6.76 (m, 2H), 3.64 – 3.59 (m, 2H), 3.46 – 3.41 (m, 3H), 3.37 – 3.32 (m, 1H), 3.14 (s, 6H), 2.18 – 2.13 (m, 2H), 1.97 (dd, *J* = 13.8, 7.8 Hz, 1H), 1.78 – 1.49 (m, 7H), 1.36 – 1.21 (m, 8H).
^{13}C NMR (151 MHz, Chloroform-*d*) mixture of diastereomers δ 180.9, 180.6, 143.7, 142.9, 133.7, 133.6, 127.8, 127.7, 123.1, 122.8, 122.5, 121.9, 108.0, 107.9, 76.1, 75.6, 67.1, 67.09, 47.0, 46.8, 43.7, 43.2, 31.7, 31.4, 26.3, 26.2, 25.8, 25.3, 25.0, 24.8.
HRMS (ESI-MS) Calcd. For $C_{15}H_{19}NO_2Na$ [M+Na]$^+$ 268.13080, found: 268.13044.
IR (neat, cm^{-1}): ṽ: 3493, 2931, 1708, 1611, 1468, 1347, 1249, 1122, 1055, 930, 750, 699.

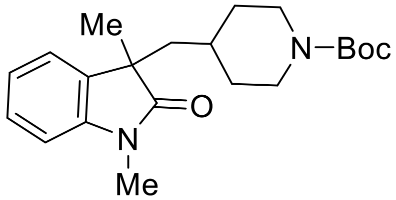

***tert*-Butyl 4-((1,3-dimethyl-2-oxoindolin-3-yl)methyl)piperidine-1-carboxylate (3ag)[6]**

The crude mixture was purified by silica gel column chromatography with pentane/EA (10:1). 53 mg product was obtained by 74% isolated yield as colorless liquid.

^{1}H NMR (600 MHz, Chloroform-*d*) δ 7.21 (t, *J* = 6.8 Hz, 1H), 7.09 (d, *J* = 7.2 Hz, 1H), 7.00 (t, *J* = 7.2 Hz, 1H), 6.79 (d, *J* = 7.8 Hz, 1H), 3.79 (s, 2H), 3.16 (s, 3H), 2.46 – 2.27 (m, 2H), 1.91 (dd, *J* = 14.4, 6.0 Hz, 1H), 1.69 (dd, *J* = 14.4, 5.4 Hz, 1H), 1.33 (s, 9H), 1.26 (s, 3H), 1.08 – 0.91 (m, 3H), 0.90 – 0.70 (m, 2H).

^{13}C NMR (151 MHz, Chloroform-*d*) δ 180.7, 154.7, 143.0, 134.0, 127.8, 122.7, 122.6, 108.1, 79.1, 47.7, 44.4, 33.2, 33.0, 32.5, 28.4, 26.3, 26.2.

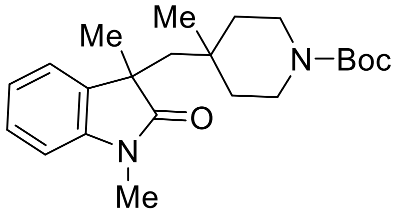

***tert*-Butyl 4-((1,3-dimethyl-2-oxoindolin-3-yl)methyl)-4-methylpiperidine-1-carboxylate (3ah)**

The crude mixture was purified by silica gel column chromatography with pentane/EA (10:1). 57 mg product was obtained by 76% isolated yield as colorless liquid.

^{1}H NMR (600 MHz, Chloroform-*d*) δ 7.22 – 7.18 (m, 1H), 7.13 (d, *J* = 7.2 Hz, 1H), 6.97 (t, *J* = 7.8 Hz, 1H), 6.79 (d, *J* = 7.8 Hz, 1H), 3.51 – 3.43 (m, 2H), 3.16 (s, 3H), 2.88 – 2.84 (m, 1H), 2.78 – 2.73 (m, 1H), 2.08 (d, *J* = 14.4 Hz, 1H), 1.83 (d, *J* = 14.4 Hz, 1H), 1.34 (s, 9H), 1.26 – 1.21 (m, 4H), 1.06 – 0.91 (m, 2H), 0.77 – 0.75 (m, 1H), 0.50 (s, 3H).

^{13}C NMR (151 MHz, Chloroform-*d*) δ 180.9, 154.8, 142.6, 134.0, 127.8, 123.7, 122.2, 108.2, 79.1, 50.1, 46.9, 38.2, 37.9, 32.8, 30.9, 28.5, 28.4, 26.3, 22.5.

HRMS (ESI-MS) Calcd. For $C_{22}H_{32}N_2O_3Na$ $[M+Na]^+$ 395.23051, found: 395.23091.

IR (neat, cm^{-1}): ṽ: 3395, 2923, 1683, 1609, 1418, 1367, 1248, 1159, 1090, 1023, 961, 862, 760, 699.

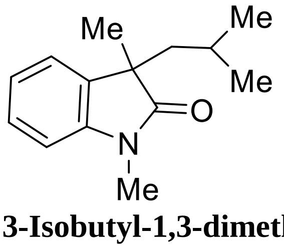

3-Isobutyl-1,3-dimethylindolin-2-one (3ai)[6]

The crude mixture was purified by silica gel column chromatography with pentane/EA (10:1). 30 mg product was obtained by 69% isolated yield as colorless liquid.

^{1}**H NMR** (600 MHz, Chloroform-*d*) δ 7.18 (t, *J* = 7.8 Hz, 1H), 7.08 (d, *J* = 7.2 Hz, 1H), 6.98 (t, *J* = 7.2 Hz, 1H), 6.77 (d, *J* = 7.8 Hz, 1H), 3.14 (s, 3H), 1.86 (dd, *J* = 13.8, 7.8 Hz, 1H), 1.68 (dd, *J* = 13.8, 5.4 Hz, 1H), 1.25 (s, 3H), 1.22 – 1.12 (m, 1H), 0.58 (d, *J* = 6.6 Hz, 3H), 0.53 (d, *J* = 6.6 Hz, 3H).

^{13}**C NMR** (151 MHz, Chloroform-*d*) δ 181.1, 143.2, 134.2, 127.6, 122.8, 122.3, 107.9, 48.1, 46.8, 26.2, 26.1, 25.5, 24.1, 22.8.

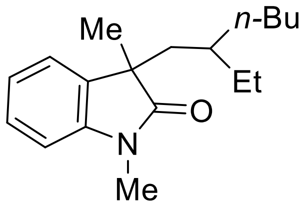

3-(2-Ethylhexyl)-1,3-dimethylindolin-2-one (3aj)

The crude mixture was purified by silica gel column chromatography with pentane/EA (10:1). 35 mg (dr = 1:1.1) product was obtained by 64% isolated yield as colorless liquid.

^{1}**H NMR** (600 MHz, Chloroform-*d*) mixture of diastereomers δ 7.24 (t, *J* = 7.8 Hz, 2H), 7.15 (dd, *J* = 7.2, 3.0 Hz, 2H), 7.04 (t, *J* = 7.8 Hz, 2H), 6.82 (d, *J* = 7.8 Hz, 2H), 3.20 (d, *J* = 3.0 Hz, 6H), 1.95 – 1.89 (m, 2H), 1.78 – 1.72 (m, 2H), 1.33 (s, 6H), 1.22 – 0.85 (m, 18H), 0.78 (t, *J* = 7.2 Hz, 3H), 0.75 (t, *J* = 7.2 Hz, 3H), 0.69 (t, *J* = 7.2 Hz, 3H), 0.64 (t, *J* = 7.2 Hz, 3H).

^{13}**C NMR** (151 MHz, Chloroform-*d*) mixture of diastereomers δ 181.0, 180.98, 143.3, 143.27, 134.3, 134.25, 127.5, 122.9, 122.8, 122.22, 122.2, 107.82, 107.8, 48.09, 48.0, 42.02, 42.0, 35.72, 35.7, 33.2, 32.8, 28.3, 28.2, 26.5, 26.2, 26.1, 25.6, 25.5, 22.8, 22.7, 14.0, 14.99, 10.3, 10.29.

HRMS (ESI-MS) Calcd. For $C_{18}H_{27}NONa$ $[M+Na]^+$ 296.19849, found: 296.19804.

IR (neat, cm^{-1}): ṽ: 3418, 2925, 1712, 1611, 1465, 1375, 1247, 1123, 1025, 929, 749, 699.

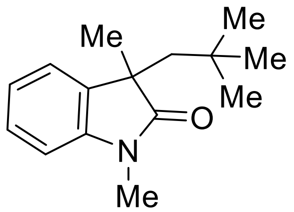

1,3-Dimethyl-3-neopentylindolin-2-one (3ak)[6]

The crude mixture was purified by silica gel column chromatography with pentane/EA (10:1). 36 mg product was obtained by 77% isolated yield as colorless liquid.

^{1}**H NMR** (600 MHz, Chloroform-*d*) δ 7.18 (d, *J* = 7.8 Hz, 1H), 7.13 (d, *J* = 7.2 Hz, 1H), 6.96 (t, *J* = 7.2 Hz, 1H), 6.78 (d, *J* = 7.8 Hz, 1H), 3.15 (s, 3H), 2.09 (d, *J* = 14.4 Hz, 1H), 1.79 (d, *J* = 14.4 Hz, 1H), 1.22 (s, 3H), 0.54 (s, 9H).

^{13}**C NMR** (151 MHz, Chloroform-*d*) δ 181.0, 142.9, 134.2, 127.5, 123.9, 122.0, 108.0, 50.8, 47.4, 31.8, 30.8, 28.3, 26.2.

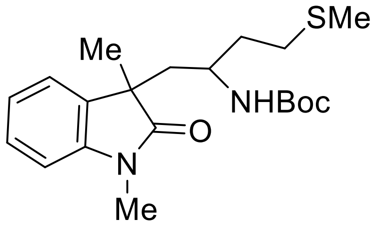

***tert*-Butyl (1-(1,3-dimethyl-2-oxoindolin-3-yl)-4-(methylthio)butan-2-yl)carbamate (3al)**

The crude mixture was purified by silica gel column chromatography with pentane/EA (10:1). 53 mg (dr = 1:1.1) product was obtained by 70% isolated yield as colorless liquid.

^{1}H NMR (600 MHz, Chloroform-*d*) mixture of diastereomers δ 7.12 – 7.17 (m, 3H), 7.09 (d, *J* = 7.2 Hz, 1H), 7.03 – 6.99 (m, 2H), 6.79 – 6.74 (m, 2H), 4.13 (d, *J* = 9.6 Hz, 1H), 3.92 (d, *J* = 9.0 Hz, 1H), 3.45 (dt, *J* = 10.2, 4.8 Hz, 1H), 3.20 (dt, *J* = 10.2, 4.8 Hz, 1H), 3.20 (s, 3H), 3.14 (s, 3H), 2.36 – 2.20 (m, 4H), 2.10 – 1.89 (m, 10H), 1.56 – 1.38 (m, 4H), 1.28 – 1.18 (m, 24H).

^{13}C NMR (151 MHz, Chloroform-*d*) mixture of diastereomers δ 181.4, 180.1, 154.7, 154.5, 143.1, 142.9, 133.9, 132.5, 128.1, 127.7, 122.75, 122.70, 122.57, 122.53, 108.4, 108.3, 78.80, 78.76, 47.9, 47.5, 47.2, 46.9, 42.5, 42.0, 36.8, 36.2, 30.3, 30.2, 28.4, 28.3, 26.35, 26.27, 25.6, 25.1, 15.47, 15.46.

HRMS (ESI-MS) Calcd. For $C_{20}H_{30}N_2O_3SNa$ $[M+Na]^+$ 401.18693 found: 401.18666.

IR (neat, cm^{-1}): ṽ: 3339, 2970, 1697, 1612, 1496, 1364, 1244, 1167, 1124, 1046, 856, 750, 699.

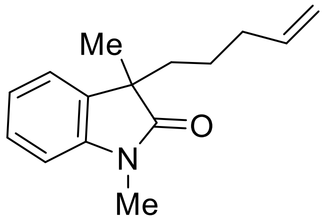

1,3-Dimethyl-3-(pent-4-en-1-yl)indolin-2-one (3am)

The crude mixture was purified by silica gel column chromatography with pentane/EA (10:1). 29 mg product was obtained by 63% isolated yield as colorless liquid.

^{1}H NMR (600 MHz, Chloroform-*d*) δ 7.22 – 7.16 (m, 1H), 7.09 (d, *J* = 7.2 Hz, 1H), 6.99 (t, *J* = 7.8 Hz, 1H), 6.76 (d, *J* = 7.8 Hz, 1H), 5.58 (m, 1H), 4.87 – 4.75 (m, 2H), 3.13 (s, 3H), 1.92 – 1.77 (m, 3H), 1.65 (t, *J* = 13.2 Hz, 1H), 1.28 (s, 3H), 1.06 – 0.98 (m, 1H), 0.89 – 0.81 (m, 1H).

^{13}C NMR (151 MHz, Chloroform-*d*) δ 180.7, 143.3, 138.2, 134.2, 127.7, 122.5, 122.46, 114.7, 107.9, 48.4, 38.0, 33.7, 26.1, 23.8.

HRMS (ESI-MS) Calcd. For $C_{15}H_{20}NO$ $[M+H]^+$ 230.15394, found: 230.15397.

IR (neat, cm^{-1}): ṽ: 3416, 2927, 2324, 2087, 1912, 1710, 1611, 1467, 1346, 1248, 1124, 1019, 911, 749, 699.

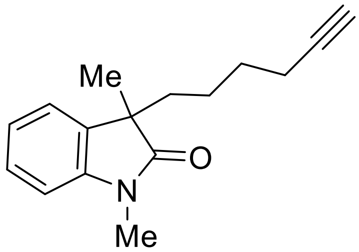

3-(Hex-5-yn-1-yl)-1,3-dimethylindolin-2-one (3an)

The crude mixture was purified by silica gel column chromatography with pentane/EA (10:1). 29 mg product was obtained by 60% isolated yield as colorless liquid.

^{1}H NMR (600 MHz, Chloroform-*d*) δ 7.25 – 7.16 (m, 1H), 7.10 (d, *J* = 7.2 Hz, 1H), 6.99 (t, *J* = 7.8 Hz, 1H), 6.77 (d, *J* = 7.8 Hz, 1H), 3.14 (s, 3H), 1.99 (t, *J* = 7.2 Hz, 2H), 1.87 – 1.76 (m, 2H), 1.67 (td, *J* = 12.6, 4.2 Hz, 1H), 1.40 – 1.25 (m, 5H), 1.06 – 0.98 (m, 1H), 0.96 – 0.85 (m, 1H).

^{13}C NMR (151 MHz, Chloroform-*d*) δ 180.7, 143.3, 134.1, 127.7, 122.5, 122.5, 107.9, 84.2, 68.3, 48.3, 37.9, 28.6, 26.1, 23.8, 23.7, 18.1.

HRMS (ESI-MS) Calcd. For $C_{16}H_{19}NONa$ $[M+Na]^+$ 264.13589, found: 264.13565.

IR (neat, cm^{-1}): ṽ: 4294, 2930, 1706, 1611, 1466, 1346, 1250, 1123, 750, 697.

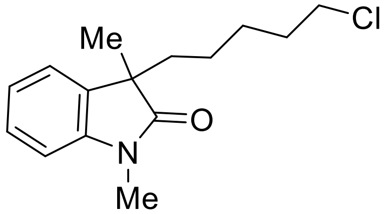

3-(5-Chloropentyl)-1,3-dimethylindolin-2-one (3ao)

The crude mixture was purified by silica gel column chromatography with pentane/EA (10:1). 37 mg product was obtained by 69% isolated yield as colorless liquid.

^{1}H NMR (600 MHz, Chloroform-*d*) δ 7.20 (t, *J* = 7.8 Hz, 1H), 7.09 (d, *J* = 7.2 Hz, 1H), 7.00 (t, *J* = 7.8 Hz, 1H), 6.77 (d, *J* = 7.8 Hz, 1H), 3.34 (t, *J* = 6.6 Hz, 2H), 3.14 (s, 3H), 1.89 – 1.79 (m, 1H), 1. 68 – 1.63 (m, 1H), 1.59 – 1.54 (m, 2H), 1.30 – 1.17 (m, 5H), 0.97 – 0.89 (m, 1H), 0.85 – 0.71 (m, 1H).

^{13}C NMR (151 MHz, Chloroform-*d*) δ 180.7, 143.3, 134.1, 127.7, 122.5, 122.4, 107.9, 48.4, 44.9, 38.2, 32.2, 26.9, 26.1, 23.9, 23.8.

HRMS (ESI-MS) Calcd. For $C_{15}H_{20}ClNONa$ $[M+Na]^+$ 288.11256, found: 288.11238.

IR (neat, cm^{-1}): ṽ: 2932, 1707, 1611, 1466, 1346, 1250, 1124, 1018, 931, 749, 700.

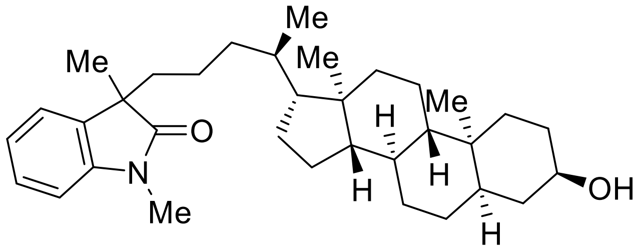

3-((*R*)-4-((3*R*,5*R*,8*R*,9*S*,10*S*,13*R*,14*S*,17*R*)-3-Hydroxy-10,13-dimethylhexadecahydro-1*H*-cyclopenta[*a*]phenanthren-17-yl)pentyl)-1,3-dimethylindolin-2-one (3ap)

The crude mixture was purified by silica gel column chromatography with pentane/EA (10:1). 64 mg product was obtained by 63% isolated yield as colorless liquid.

^{1}H NMR (600 MHz, Chloroform-*d*) δ 7.22 – 7.17 (m, 1H), 7.10 (d, *J* = 7.2 Hz, 1H), 7.00 (t, *J* = 7.2 Hz, 1H), 6.77 (d, *J* = 7.8 Hz, 1H), 3.57 – 3.52 (m, 1H), 3.15 (s, 3H), 1.84 – 0.71 (m, 37H), 0.66 (d, *J* = 6.6 Hz, 3H), 0.51 (s, 3H).

^{13}C NMR (151 MHz, Chloroform-*d*) δ 180.9, 143.3, 134.4, 127.6, 122.5, 122.4, 107.86, 71.9, 56.5, 56.4, 48.5, 42.7, 42.1, 40.4, 40.2, 38.8, 36.5, 36.0, 35.8, 35.5, 35.4, 34.6, 30.6, 28.3, 27.2, 26.4, 26.1, 24.2, 23.8, 23.4, 21.2, 20.8, 18.5, 12.0.

HRMS (ESI-MS) Calcd. For $C_{34}H_{51}NO_2Na$ $[M+Na]^+$528.38120, found: 528.37952.

IR (neat, cm^{-1}): ṽ: 3414, 2928, 2245, 1701, 1612, 1464, 1375, 1251, 1123, 1037, 912, 852.

4. Mechanism studies

4.1. The radical trapping experiment

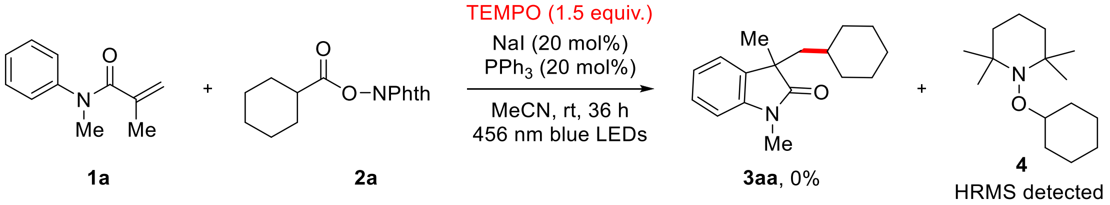

Procedure: A clean oven-dried tube equipped with a PTFE-coated stirring bar was charged with arylamide **1a** (53 mg, 0.3 mmol), cyclohexyl redox-active ester **2a** (55 mg, 0.2 mmol), PPh_3 (10.5 mg, 0.04 mmol), NaI (6 mg, 0.04 mmol) and TEMPO (46.8 mg, 0.3 mmol), then, the system was degassed and filled with nigrogen gas three times before the solvent anhydrous MeCN (2 mL) was added. The reaction was carried out irradiated using 40 W 456 nm blue LEDs at room temperature under fan cooling. After 36 h, the solvent was removed in vacuo, the desired product **3aa** was not detected by NMR of the crude reaction mixture. HRMS of the crude reaction mixute shows the existence of trapped product **4**. HRMS (ESI-MS) Calcd. For $C_{15}H_{30}NO$ $[M+H]^+$ 240.23219, found: 240.23171.

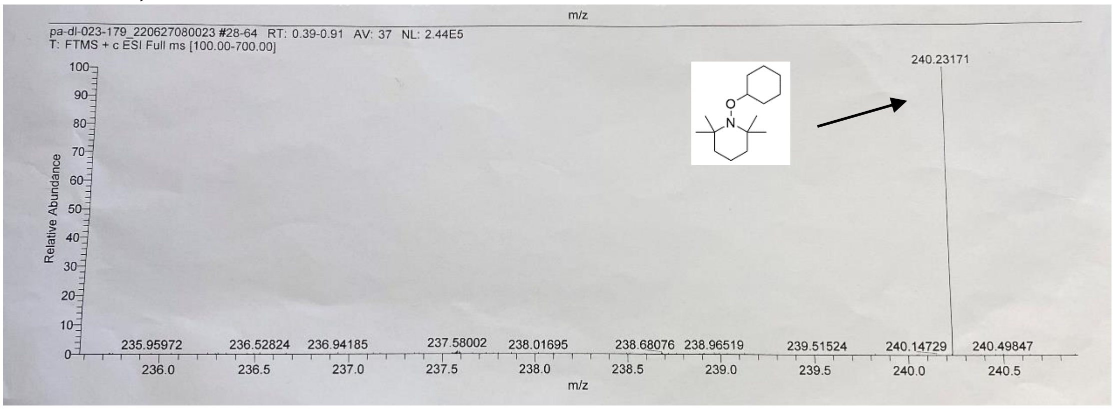

4.2. The radical clock experiment

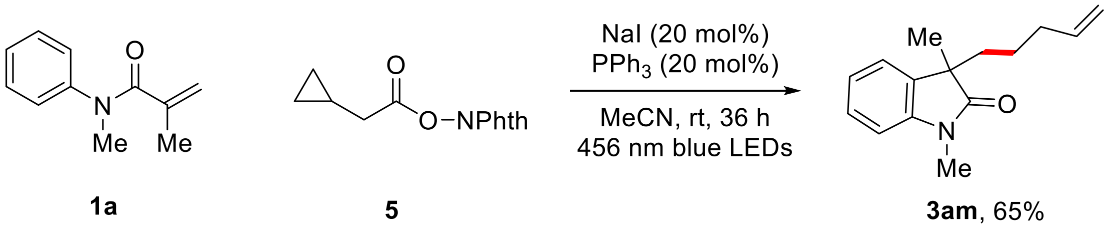

Procedure: To a reaction tube with a stir bar, arylamide **1a** (53 mg, 0.3 mmol), redox-active ester **5** (49 mg, 0.2 mmol), PPh_3 (10.5 mg, 0.04 mmol), NaI (6 mg, 0.04 mmol) was added. After degassing and filling with nitrogen gas three times, the anhydrous solvent MeCN was added. The reaction was performed under blue LEDs irradiation (40 W, 456 nm) at room temperature under fan cooling. After 36 h, the crude reaction mixture was concentrated in vacuo and purified by silica gel column chromatography (Pentane/EA = 10:1) to obtain the product. Colorless liquid: 30 mg, 65% yield, The compound data was in agreement with product **3am**.

5. References

[1] M. Zhang, X. Ding, A. Lu, J. Kang, Yongyue Gao, Z. Wang, H. Li, Q. Wang, *Org. Chem. Front.* **2021**, *8*, 961-967.
[2] J. Xu, Z. Yang, J. Hua, Y. Lin, M. Bian, Y. Li, C. Liu, W. He, Z. Fang, K. Guo, *Org. Chem. Front.* **2020**, *7,* 3223-3228.
[3] J. Cornella, J. T. Edwards, T. Qin, S. Kawamura, J. Wang, C.-M. Pan, R. Gianatassio, M. Schmidt, M. D. Eastgate, P. S. Baran, *J. Am. Chem. Soc.* **2016**, *138*, 2174–2177.
[4] H. Wang, L.−N. Guo, X.-H. Duan, *J. Org. Chem.* **2016**, *81*, 860−867.
[5] J. Du, X. Wang, H. Wang, J. Wei, X. Huang, J. Song, Junmin Zhang, *Org. Lett.* **2021**, *23*, 5631-5635.
[6] K. Muralirajan, R. Kancherla, A. Gimnkhan, M. Rueping, *Org. Lett*. **2021**, *23*, 6905-6910.

6. NMR Spectra of products

3aa ^{1}H NMR (600 MHz, Chloroform-*d*)

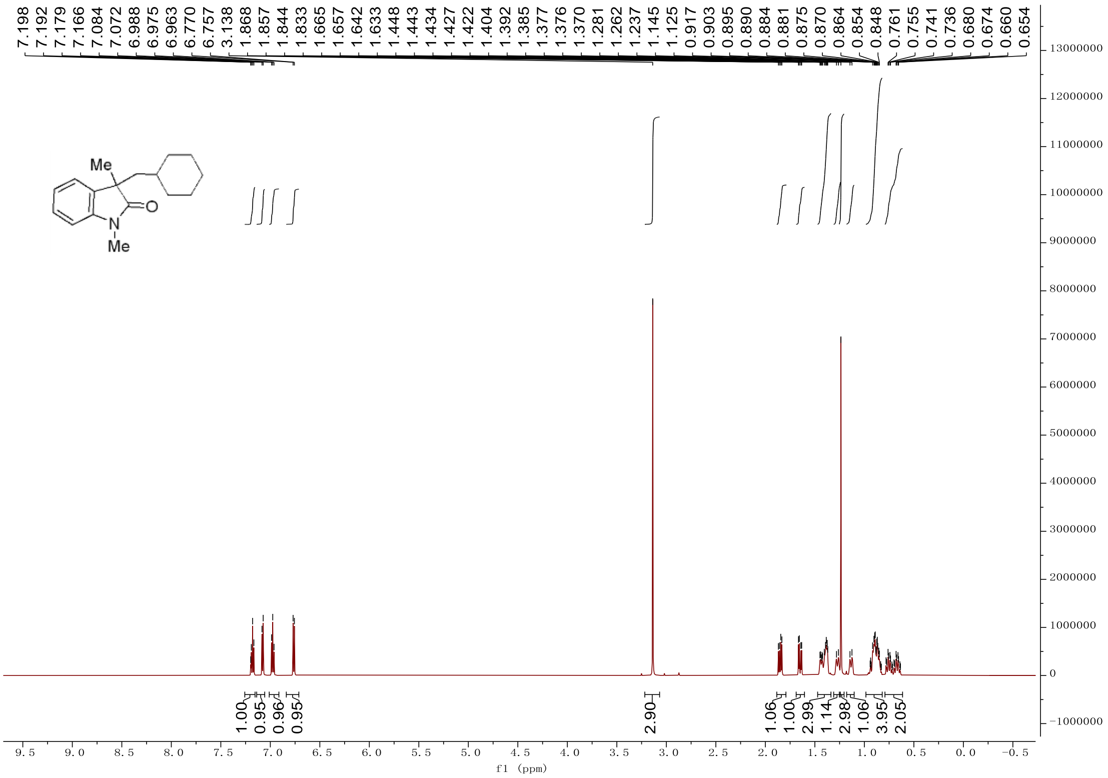

3aa ^{13}C NMR (151 MHz, Chloroform-*d*)

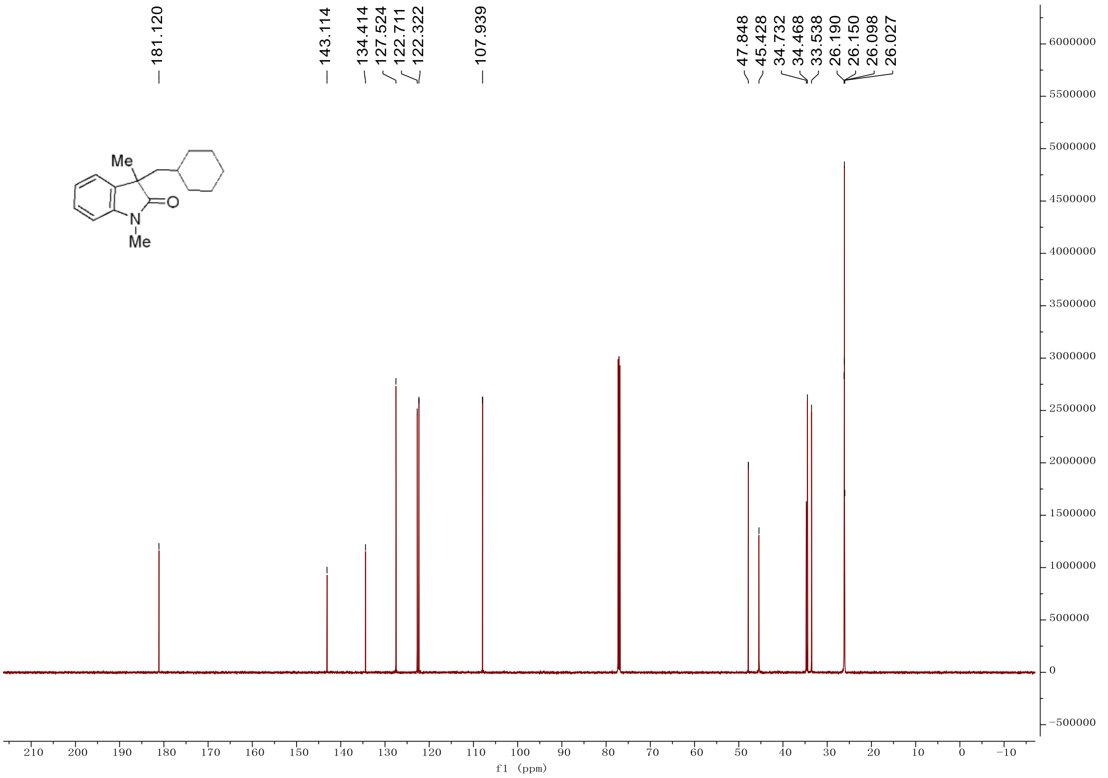

3ba ^{1}H NMR (600 MHz, Chloroform-*d*)

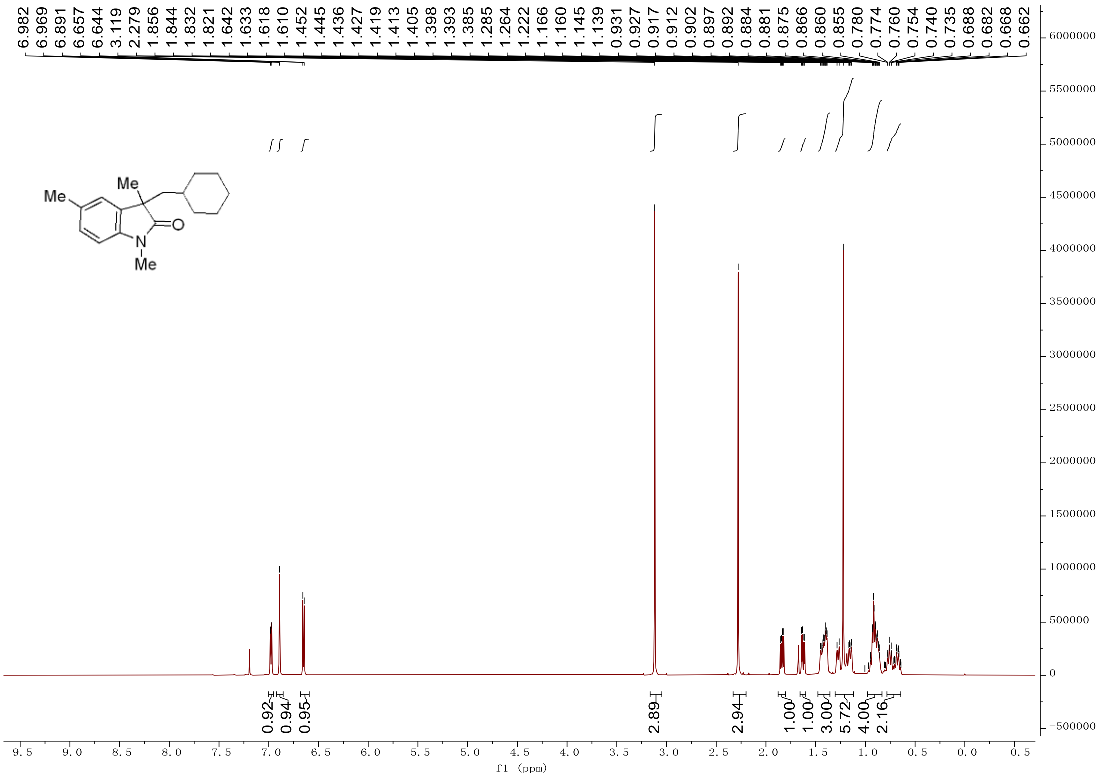

3ba ^{13}C NMR (151 MHz, Chloroform-*d*)

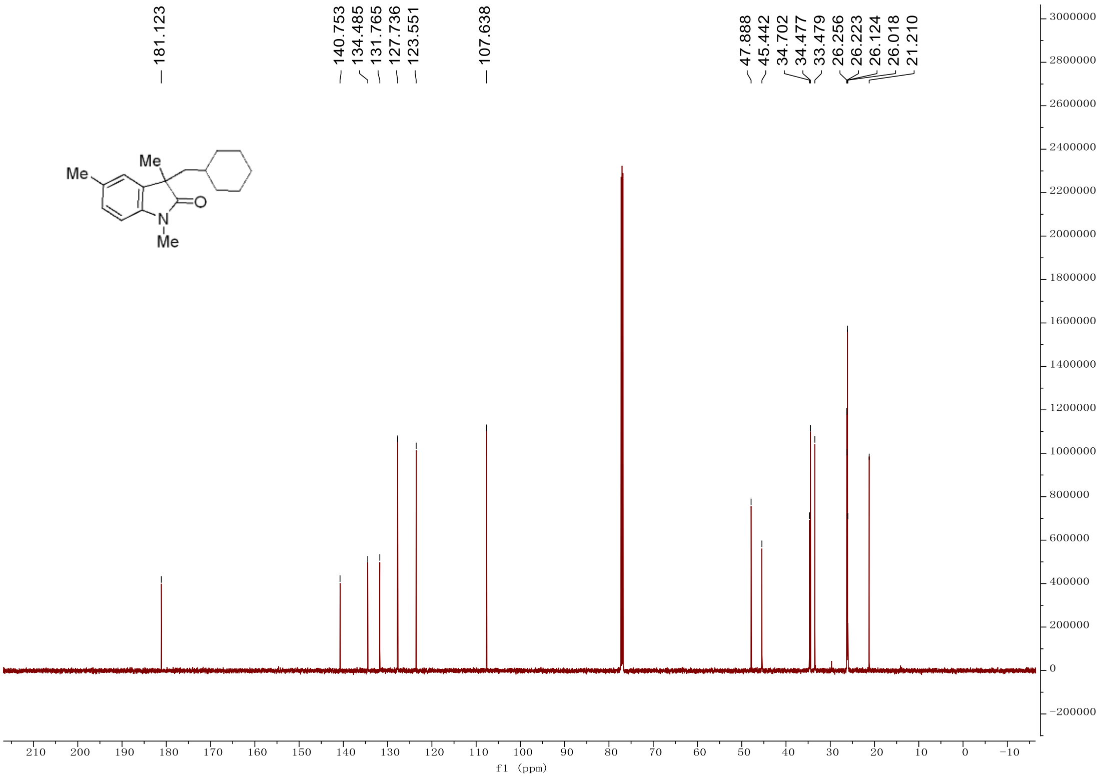

3ca ^{1}H NMR (600 MHz, Chloroform-*d*)

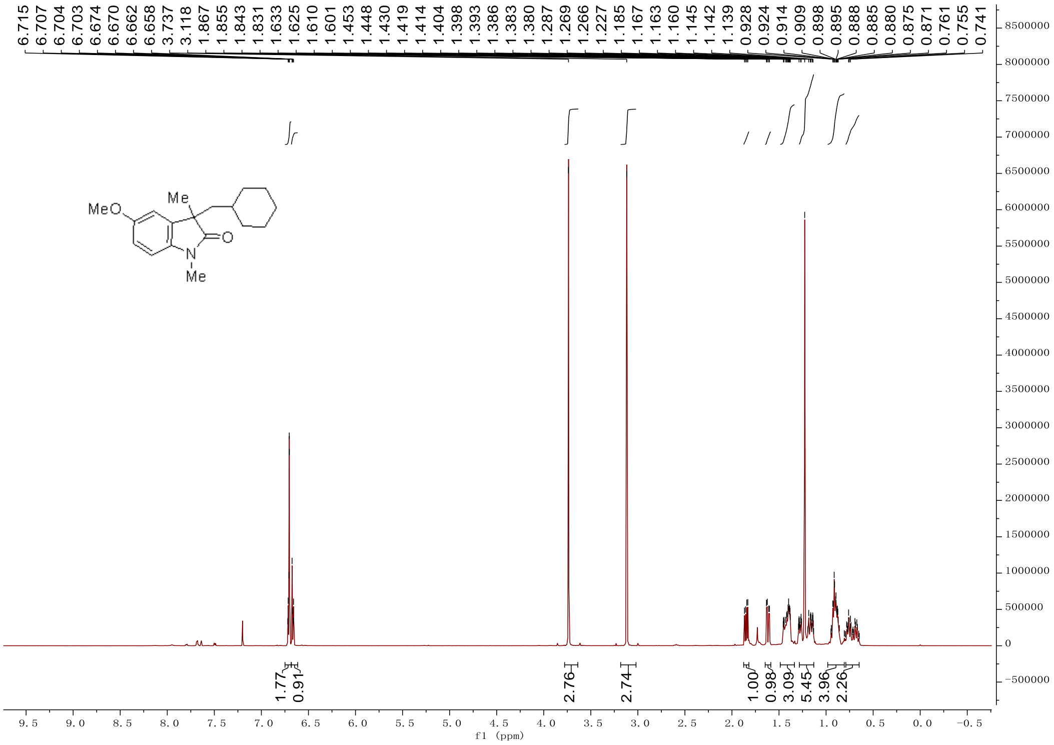

3ca ^{13}C NMR (151 MHz, Chloroform-*d*)

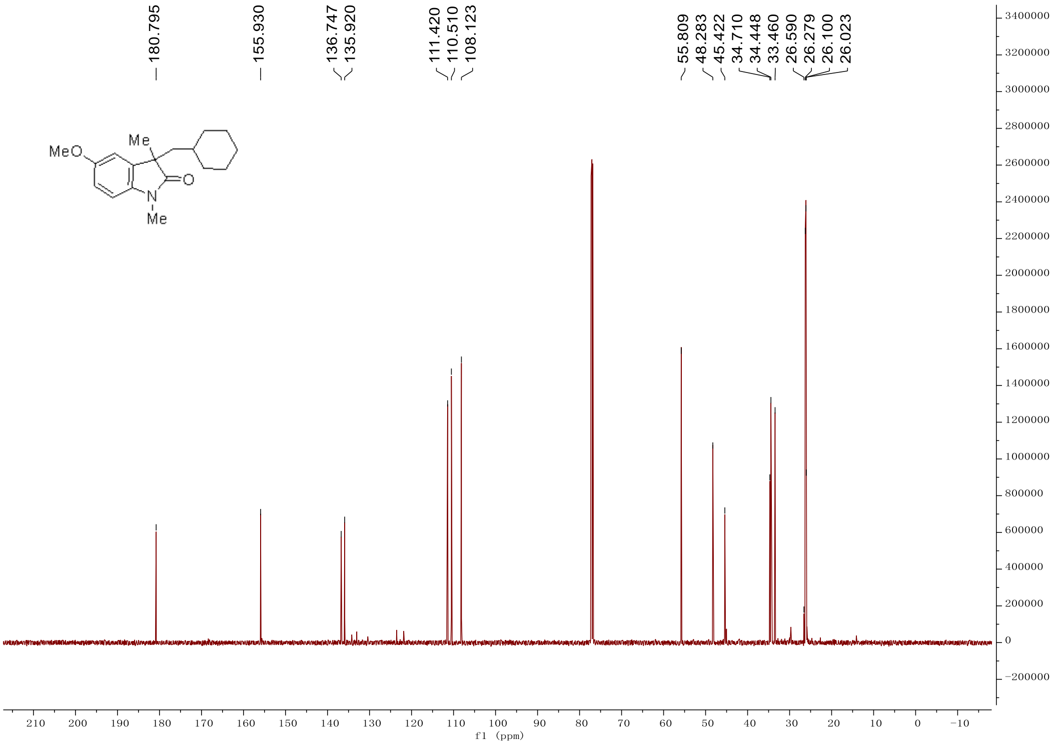

3ca (second purification) ^{1}H NMR (400 MHz, Chloroform-*d*)

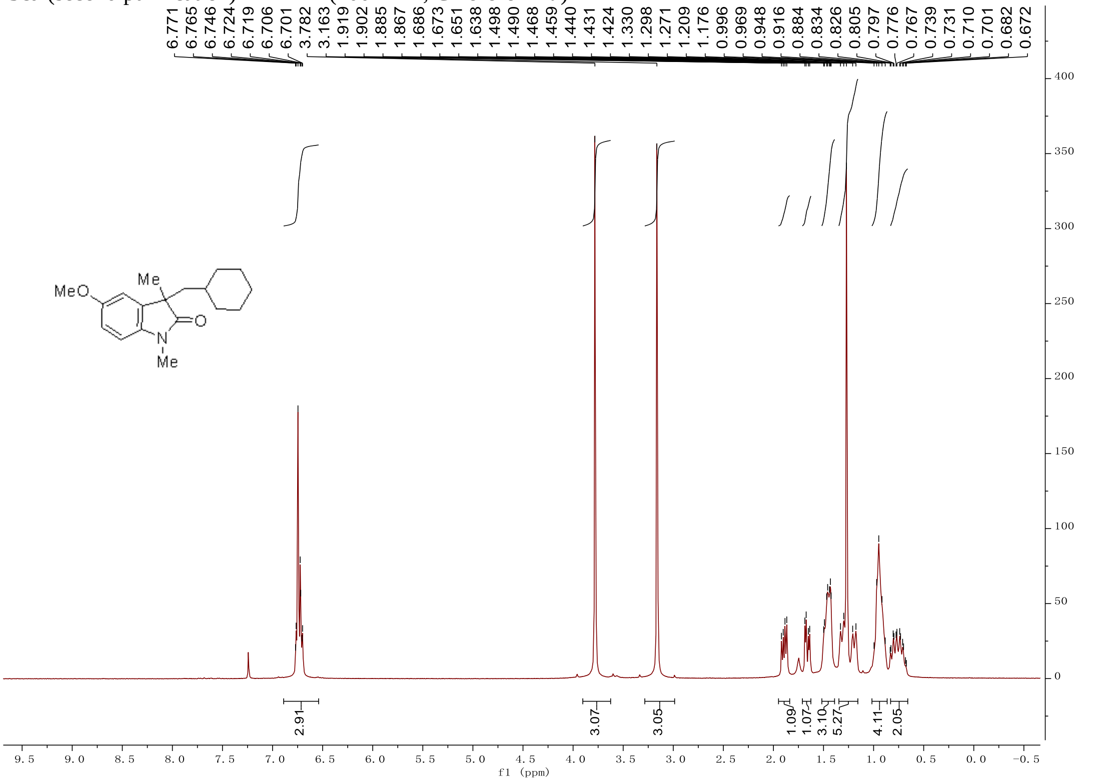

3ca (second purification) ^{13}C NMR (151 MHz, Chloroform-*d*)

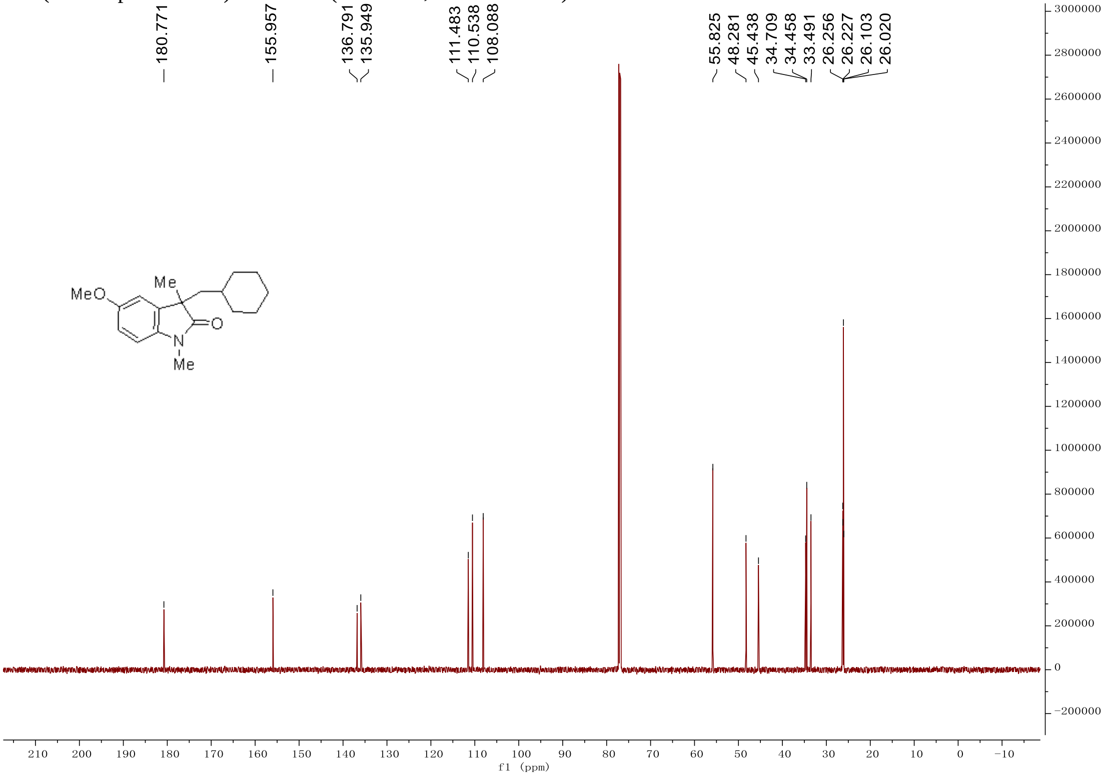

3da ^{1}H NMR (400 MHz, Chloroform-*d*)

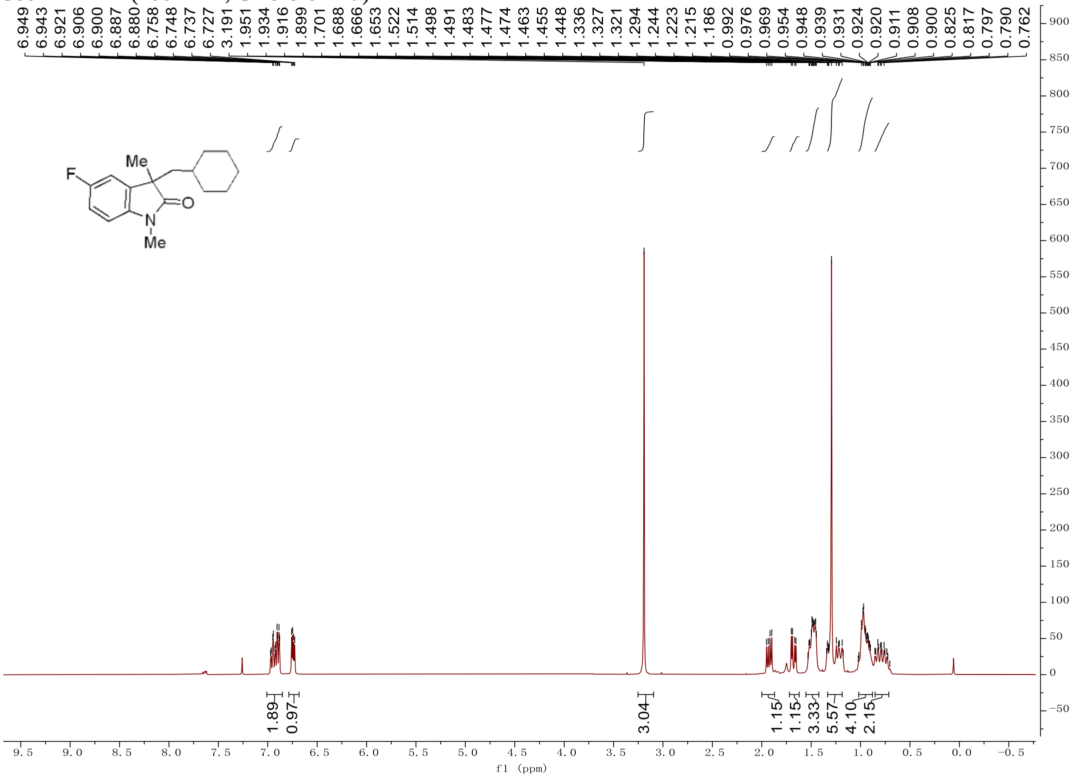

3da ^{13}C NMR (101 MHz, Chloroform-*d*)

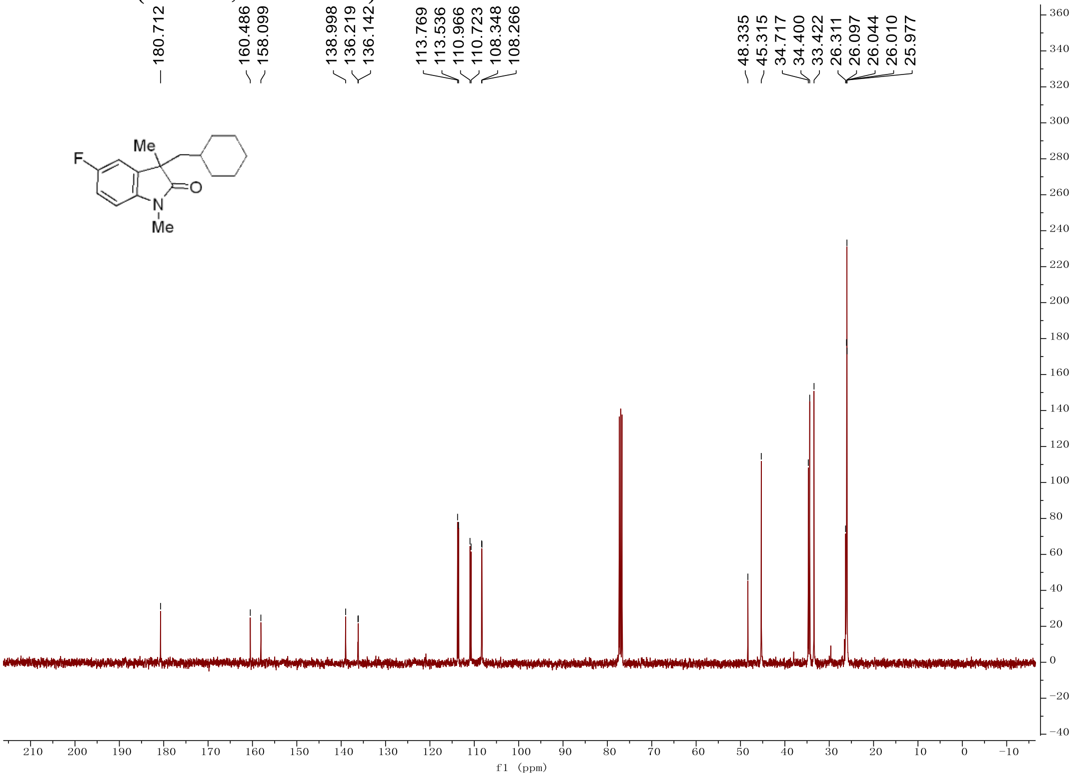

3da ^{19}F NMR (376 MHz, Chloroform-d)

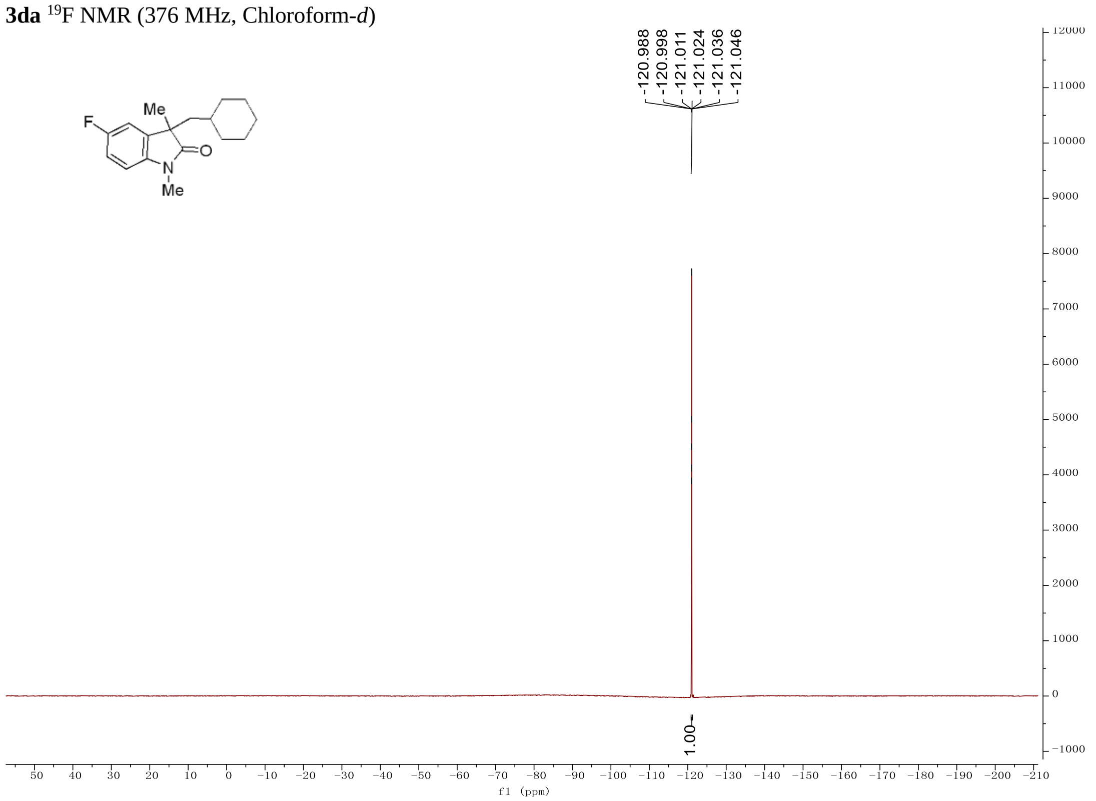

3ea ^{1}H NMR (600 MHz, Chloroform-*d*)

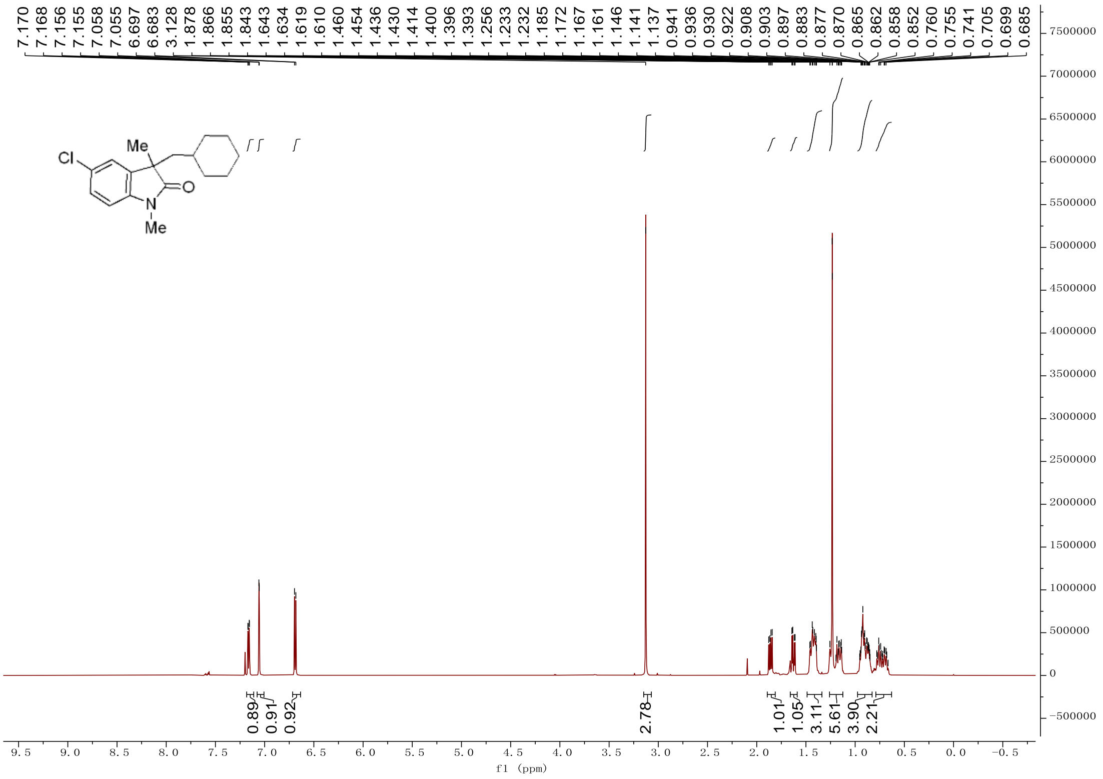

3ea ^{13}C NMR (151 MHz, Chloroform-*d*)

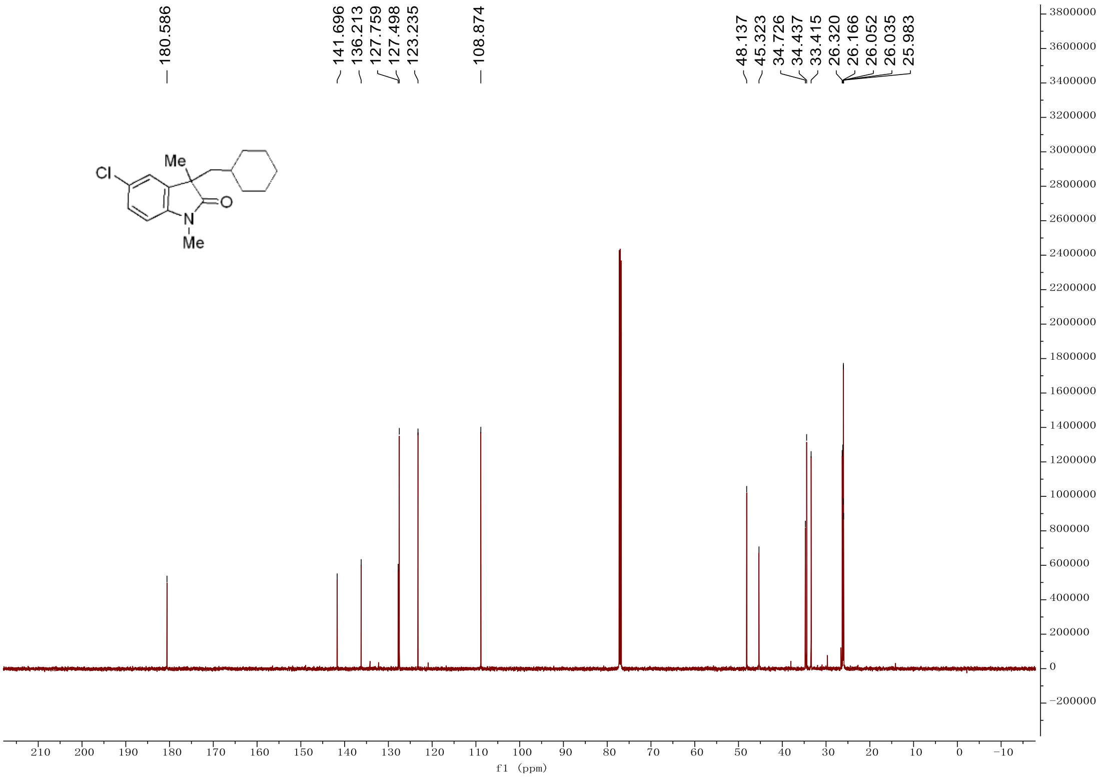

3fa ^{1}H NMR (600 MHz, Chloroform-*d*)

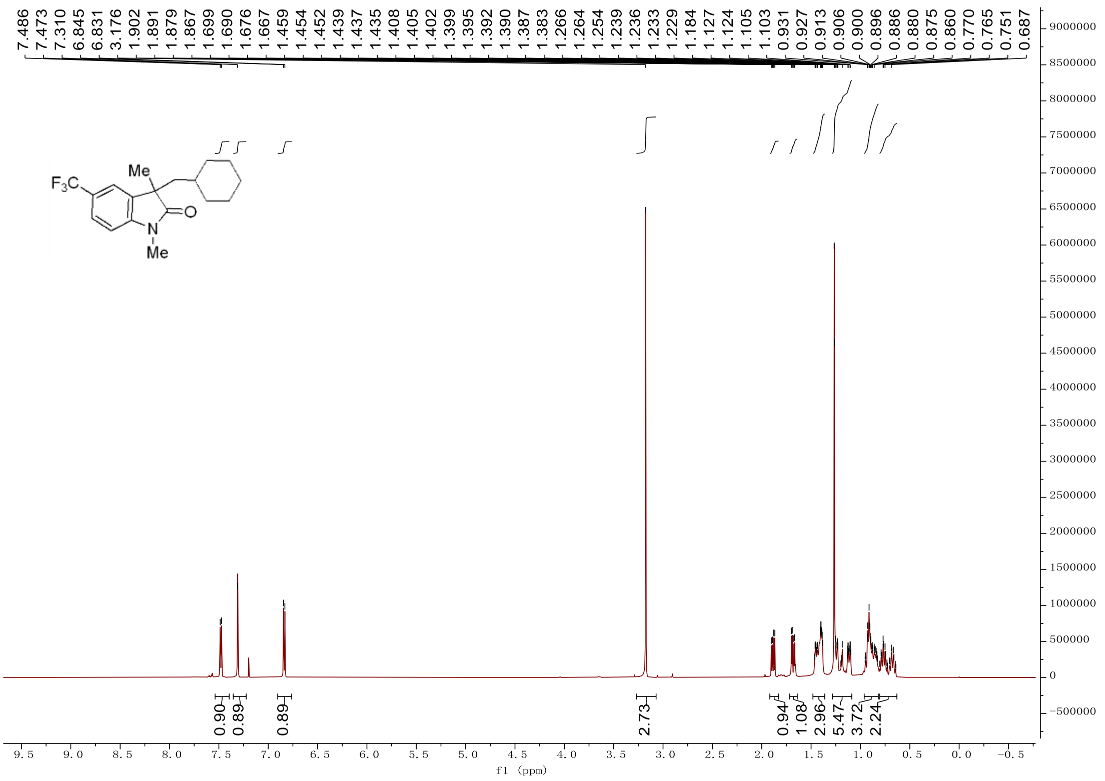

3fa ^{13}C NMR (151 MHz, Chloroform-*d*)

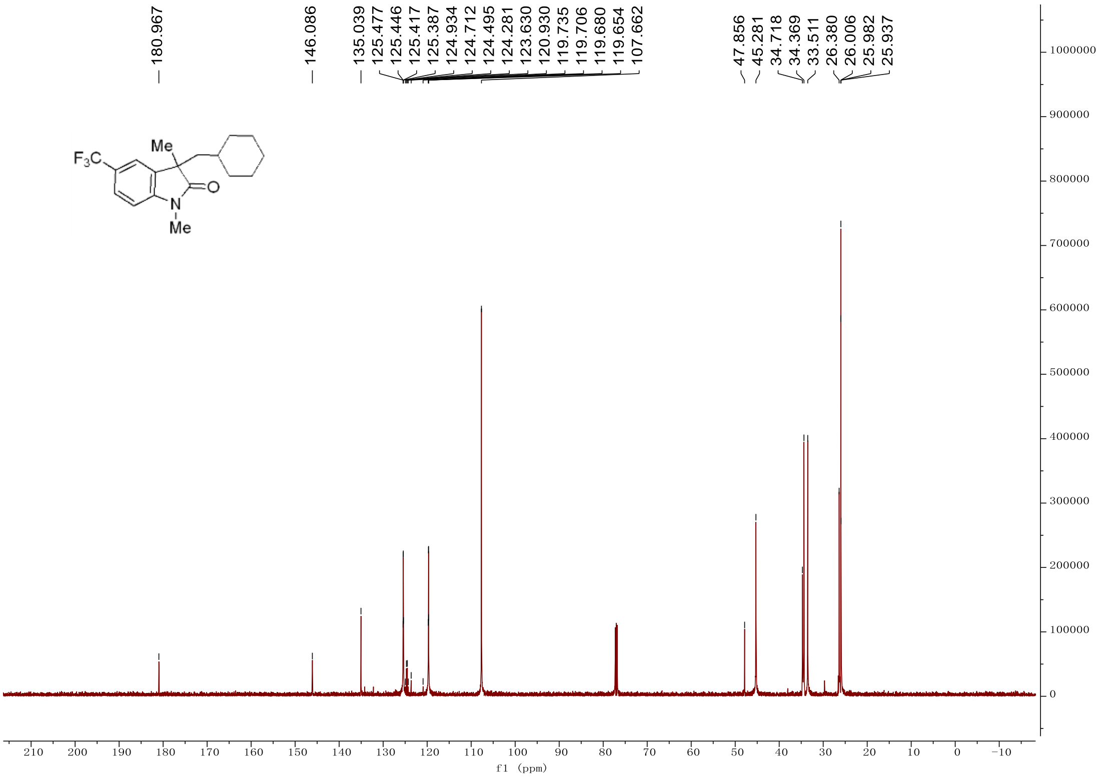

3fa ^{19}F NMR (282 MHz, Chloroform-*d*)

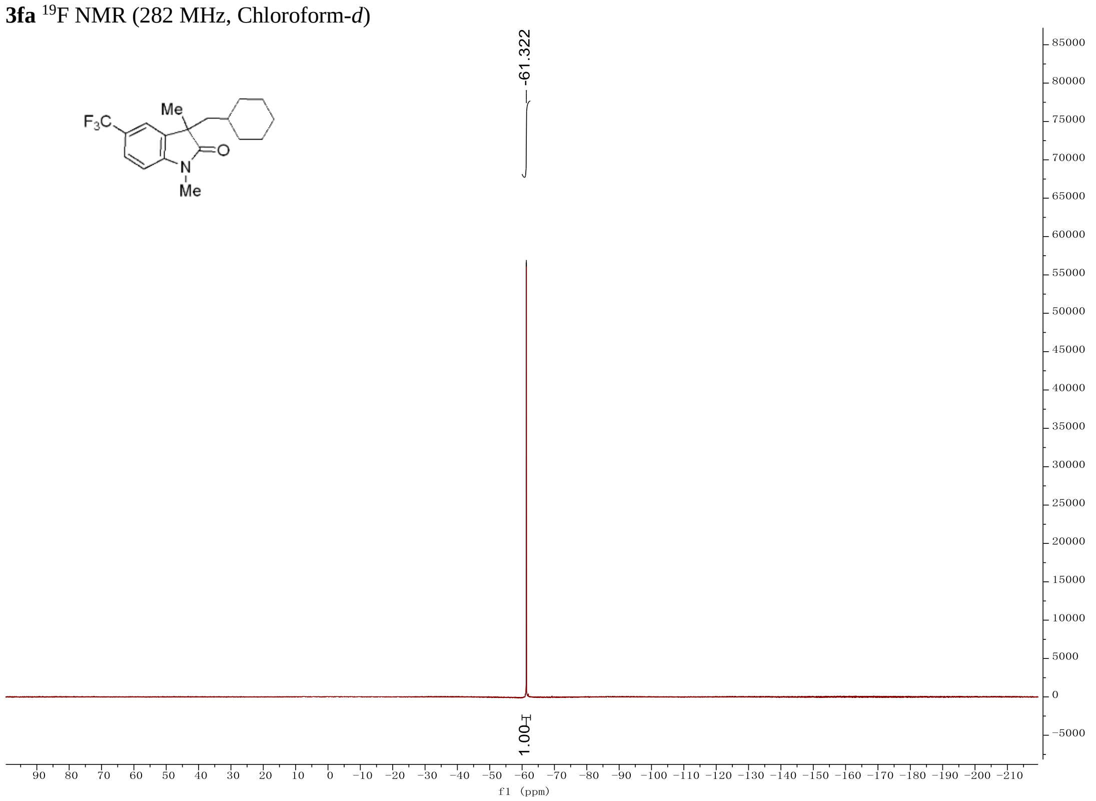

3ga ^{1}H NMR (600 MHz, Chloroform-*d*)

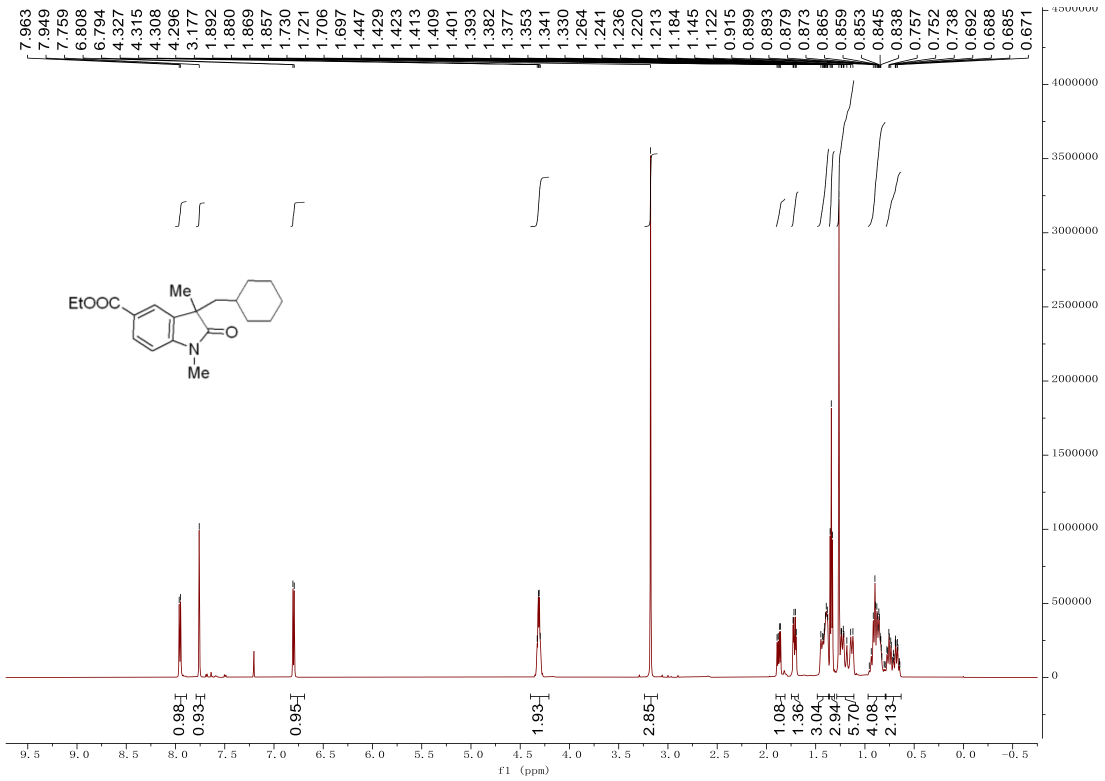

3ga ^{13}C NMR (151 MHz, Chloroform-*d*)

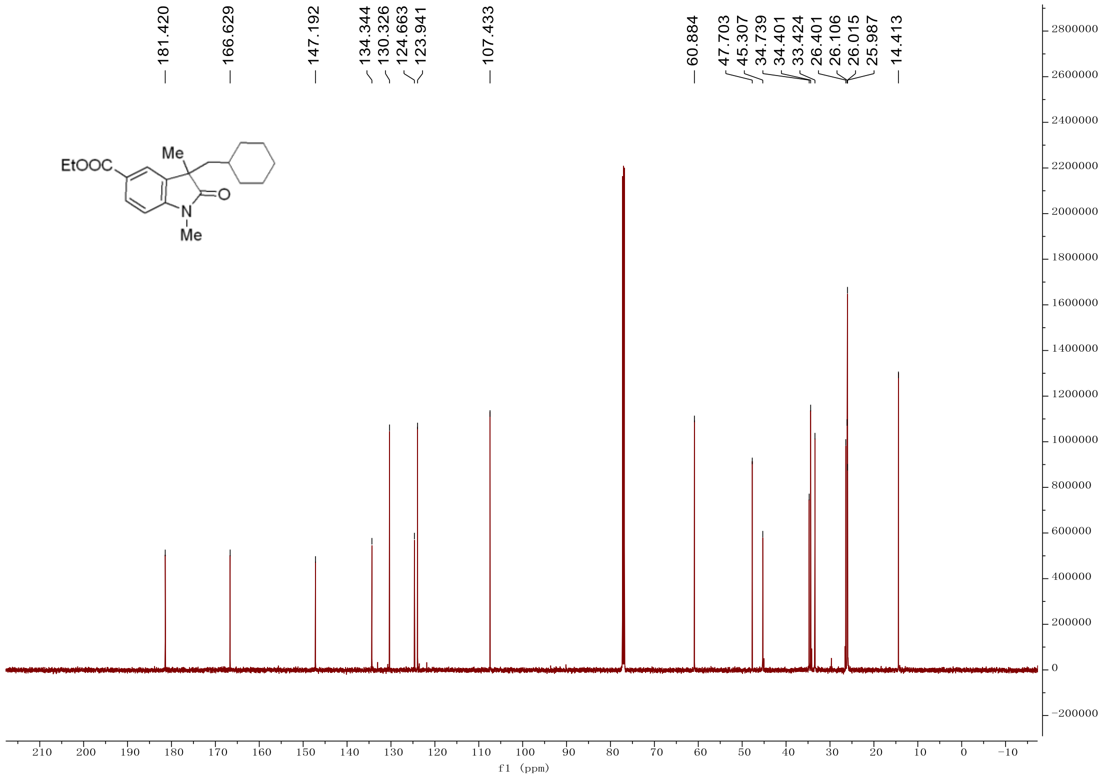

3ha ^{1}H NMR (600 MHz, Chloroform-*d*)

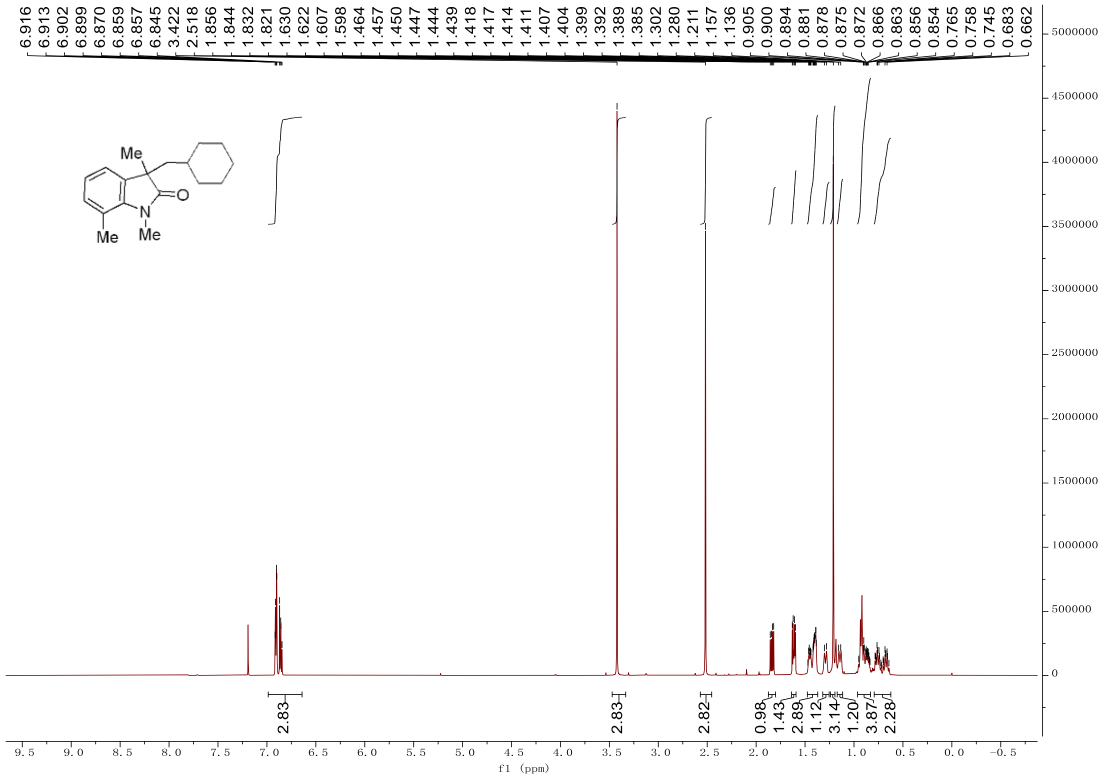

3ha ^{13}C NMR (151 MHz, Chloroform-*d*)

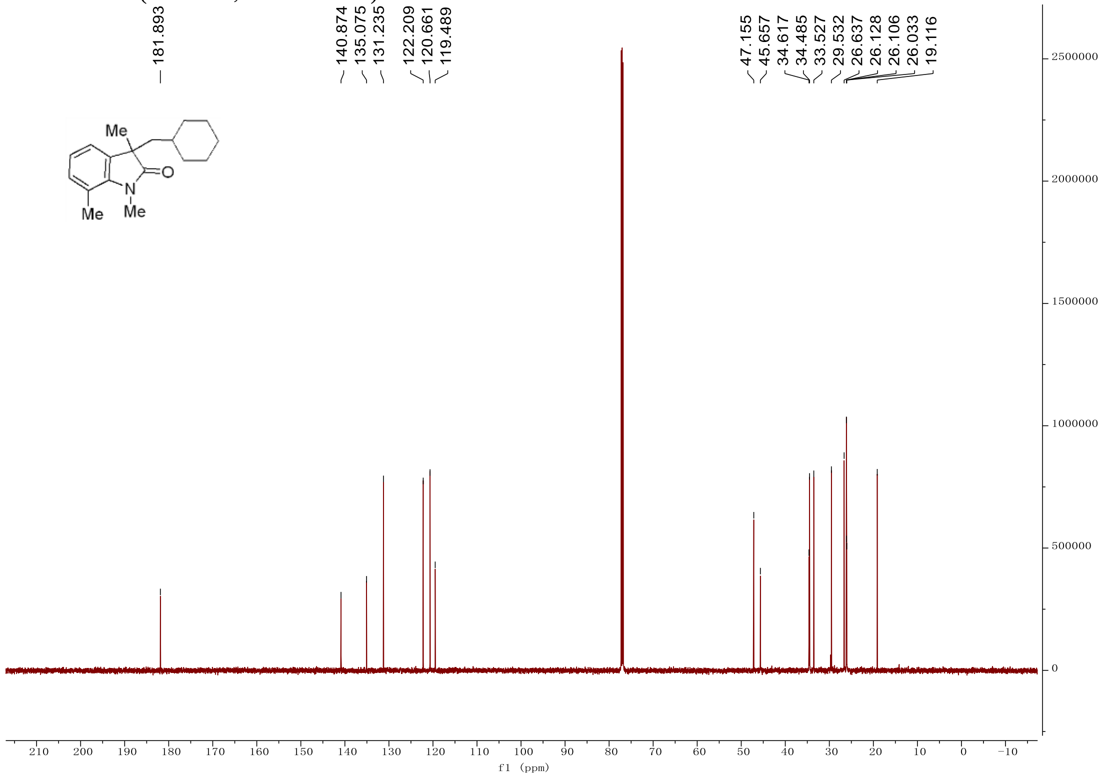

3ia ^{1}H NMR (600 MHz, Chloroform-*d*)

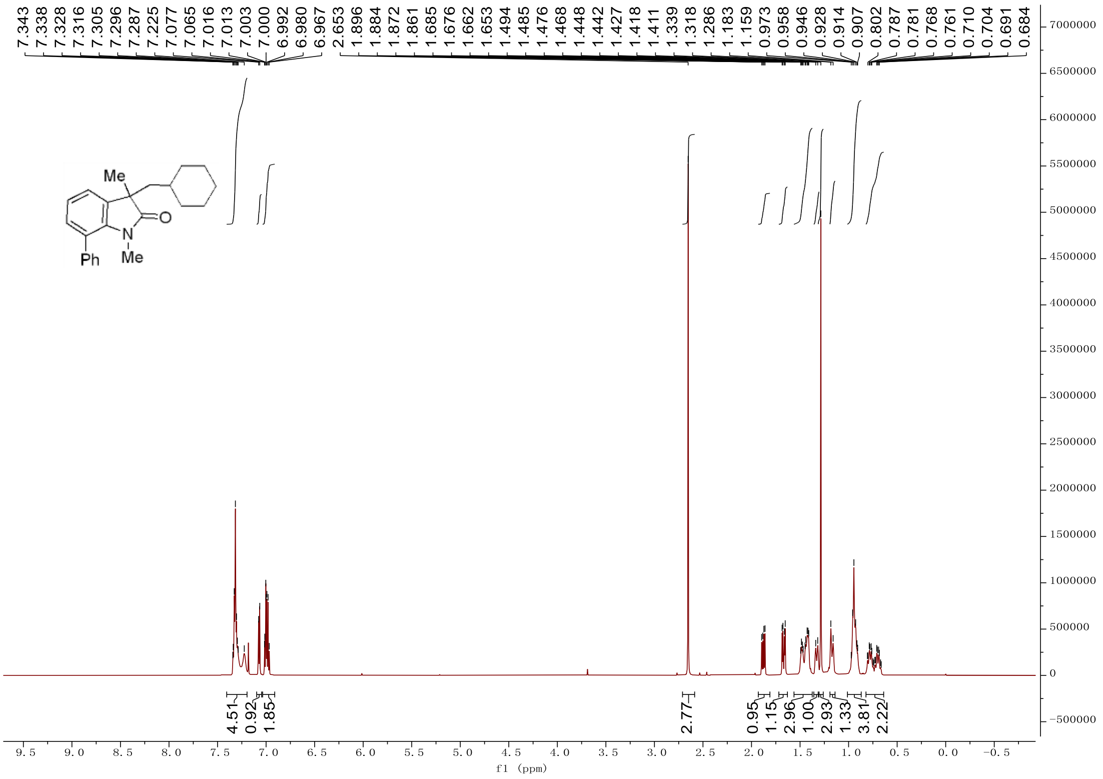

3ia ^{13}C NMR (151 MHz, Chloroform-*d*)

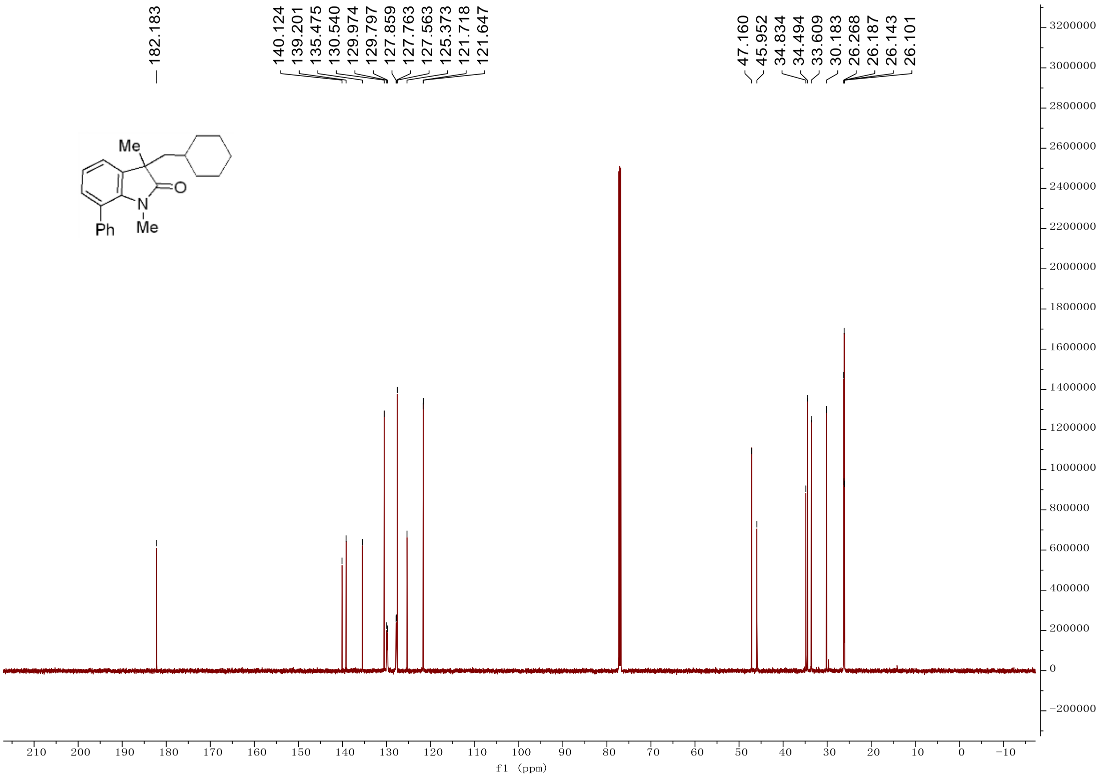

3ja ^{1}H NMR (600 MHz, Chloroform-*d*)

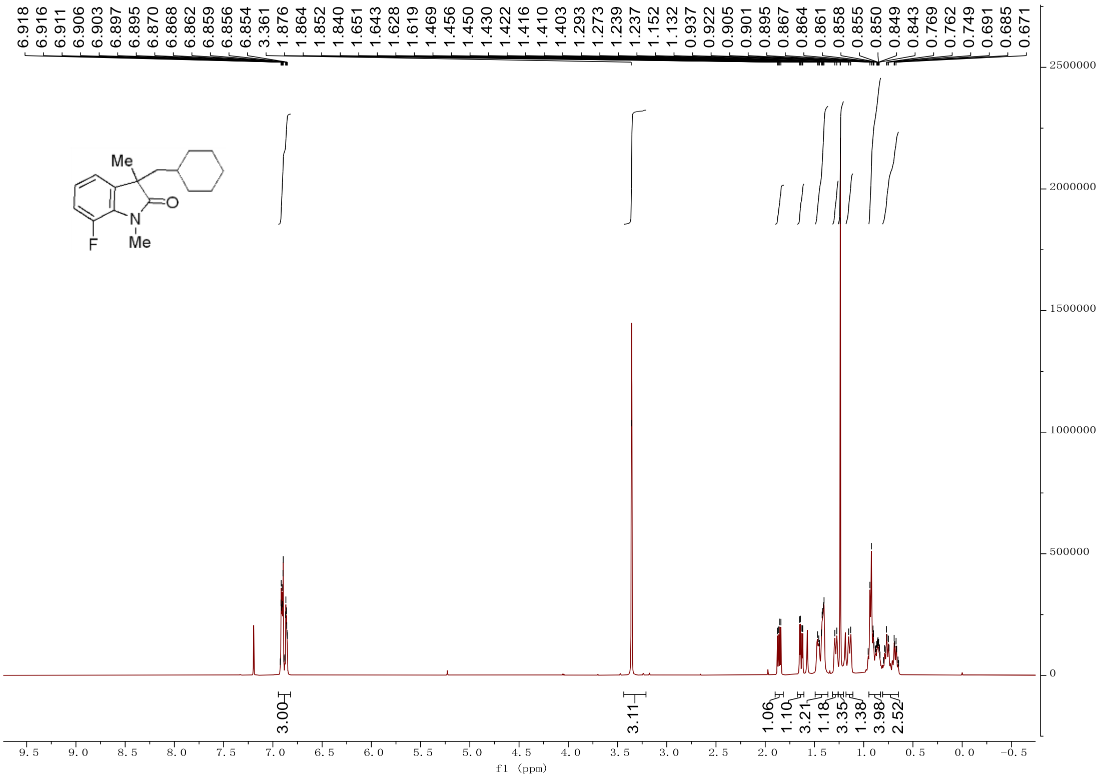

3ja ^{13}C NMR (151 MHz, Chloroform-*d*)

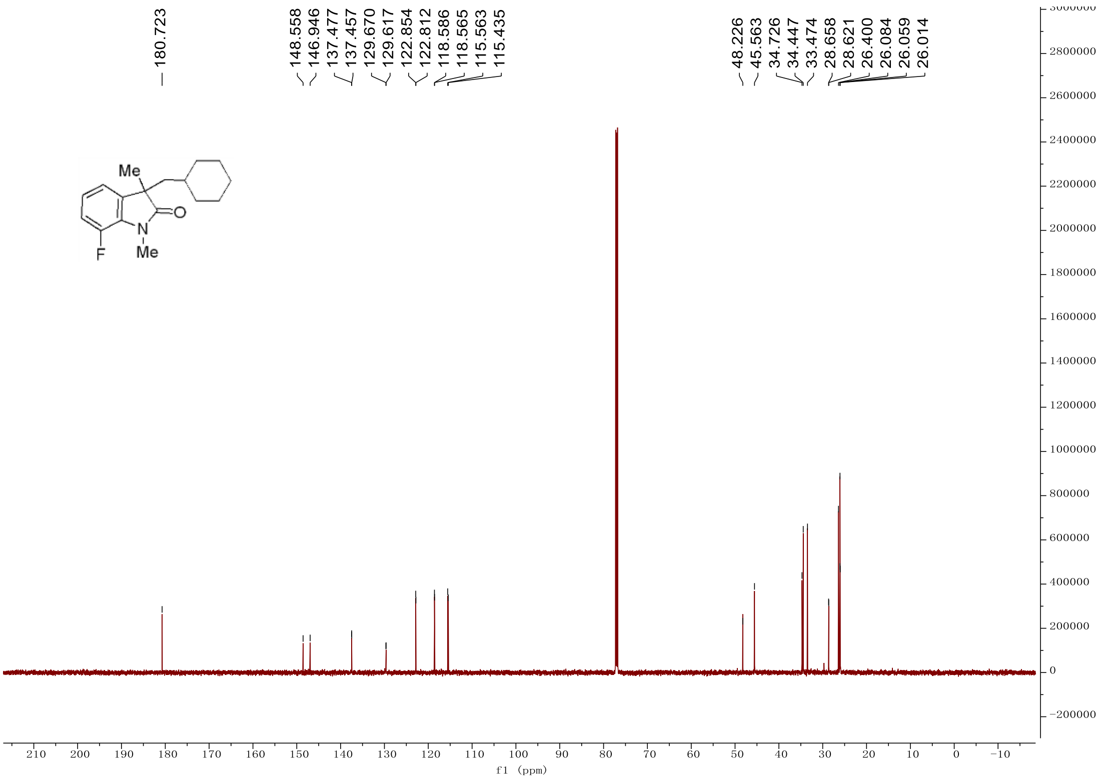

3ja ^{19}F NMR (564 MHz, Chloroform-*d*)

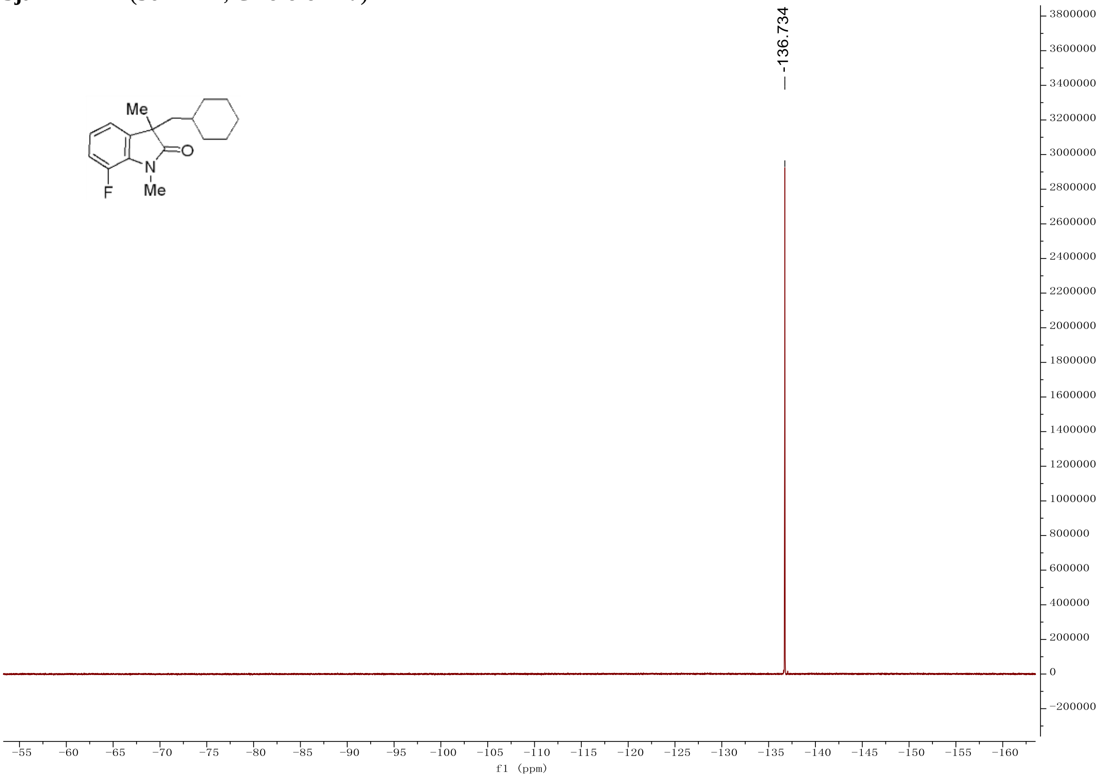

3ka ^{1}H NMR (600 MHz, Chloroform-*d*)

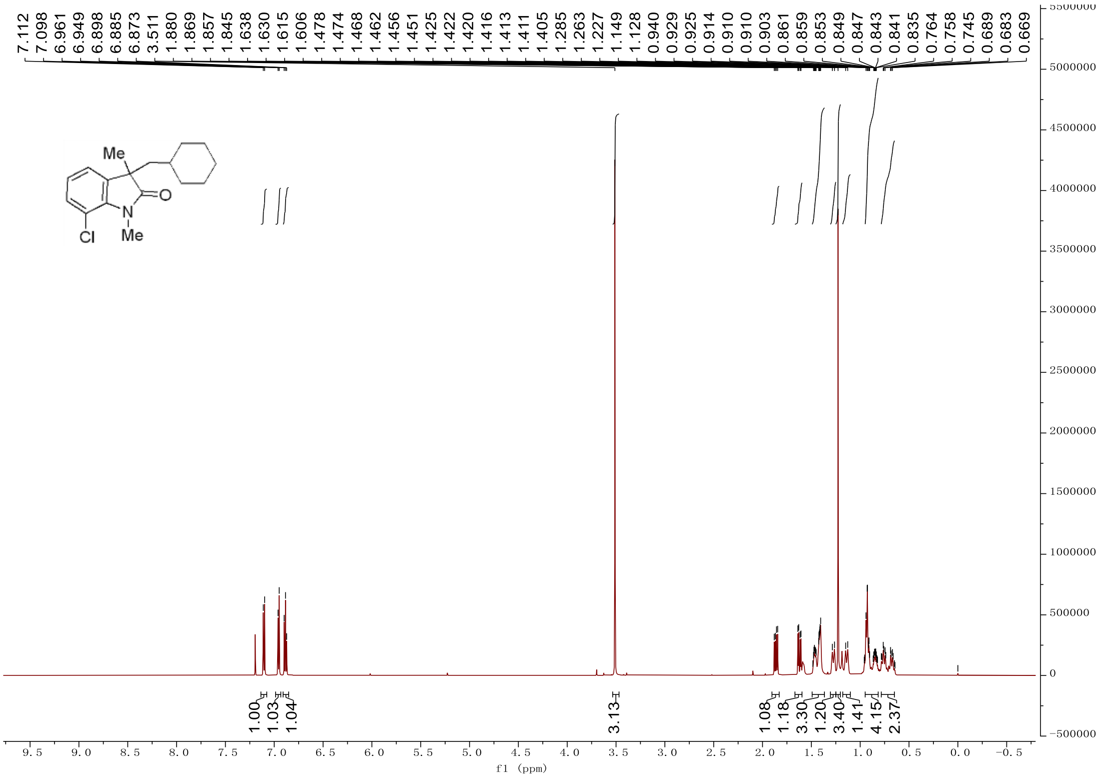

3ka ^{13}C NMR (151 MHz, Chloroform-*d*)

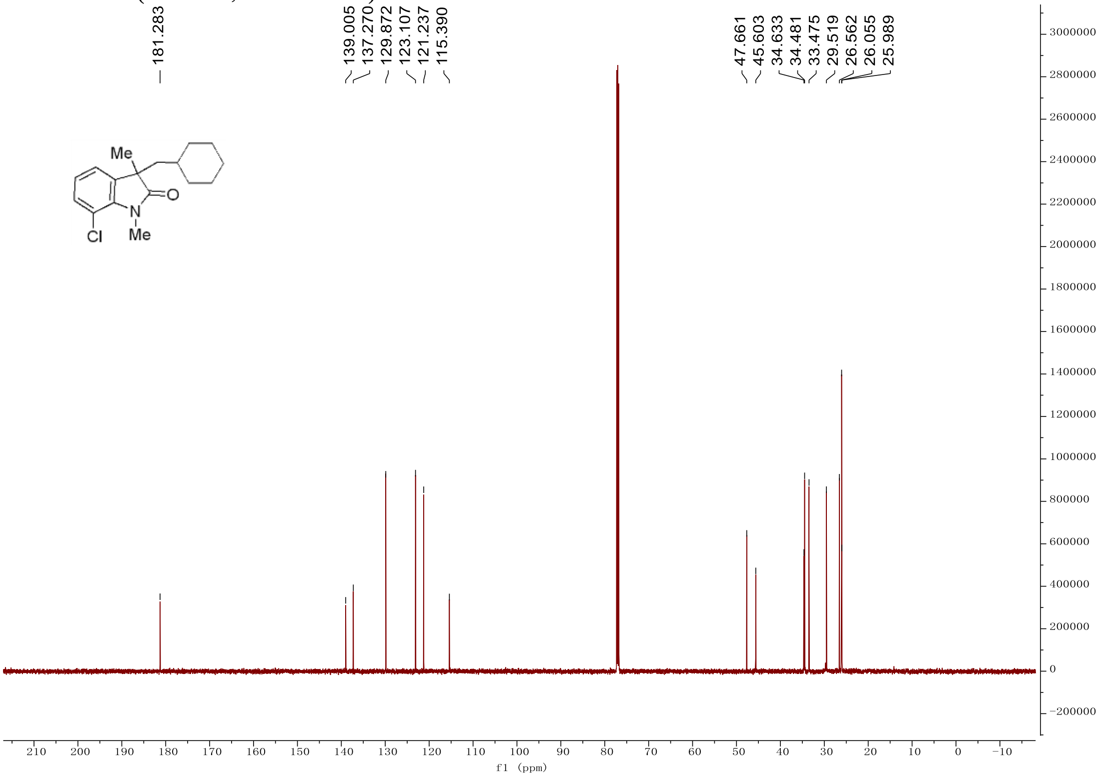

3la ^{1}H NMR (600 MHz, Chloroform-*d*)

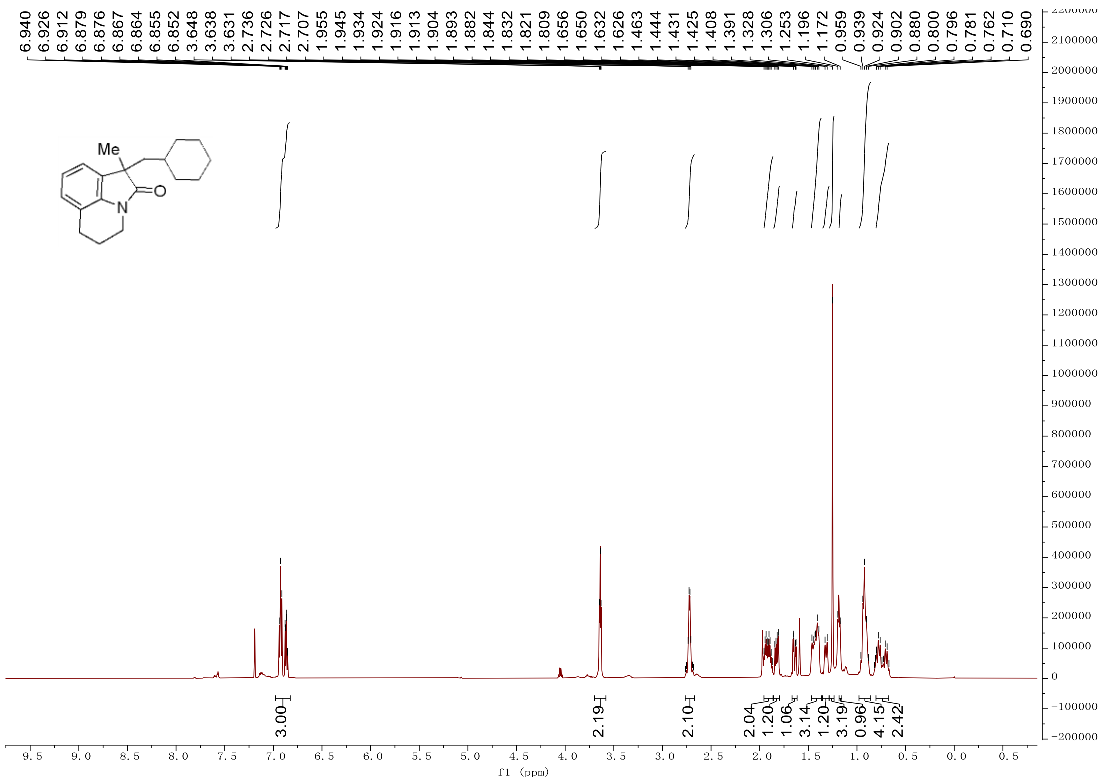

3la ^{13}C NMR (151 MHz, Chloroform-*d*)

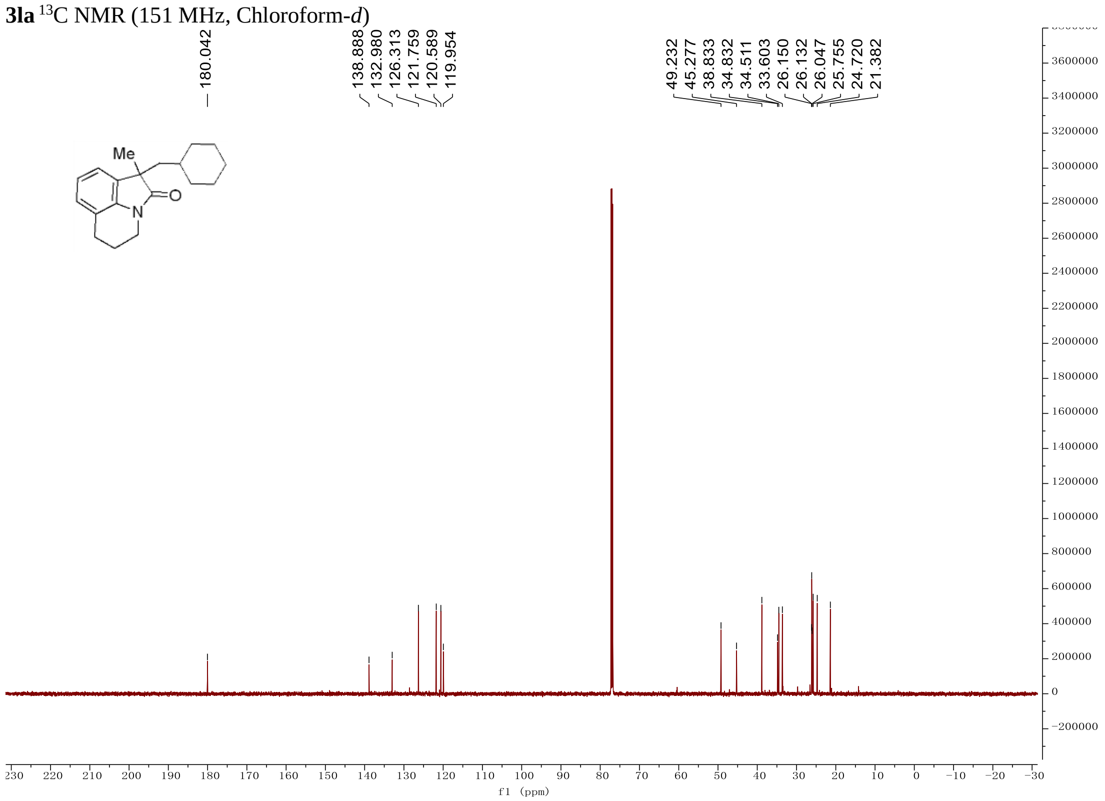

3ma ^{1}H NMR (600 MHz, Chloroform-*d*)

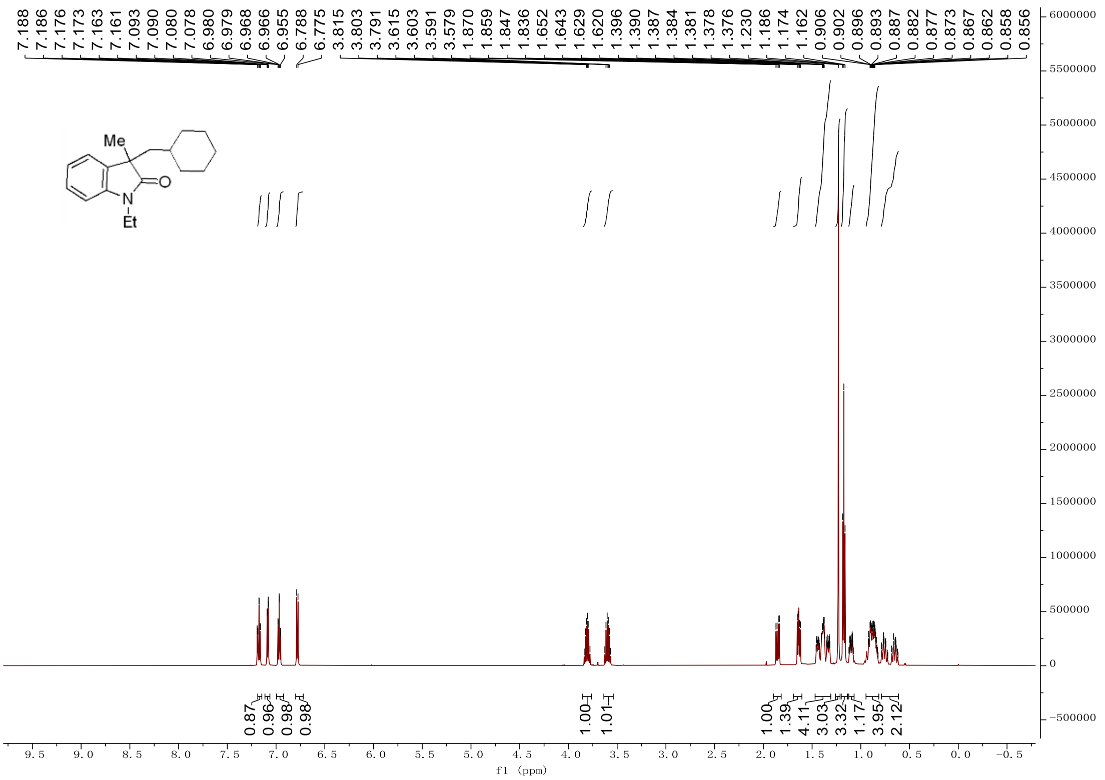

3ma ^{13}C NMR (151 MHz, Chloroform-*d*)

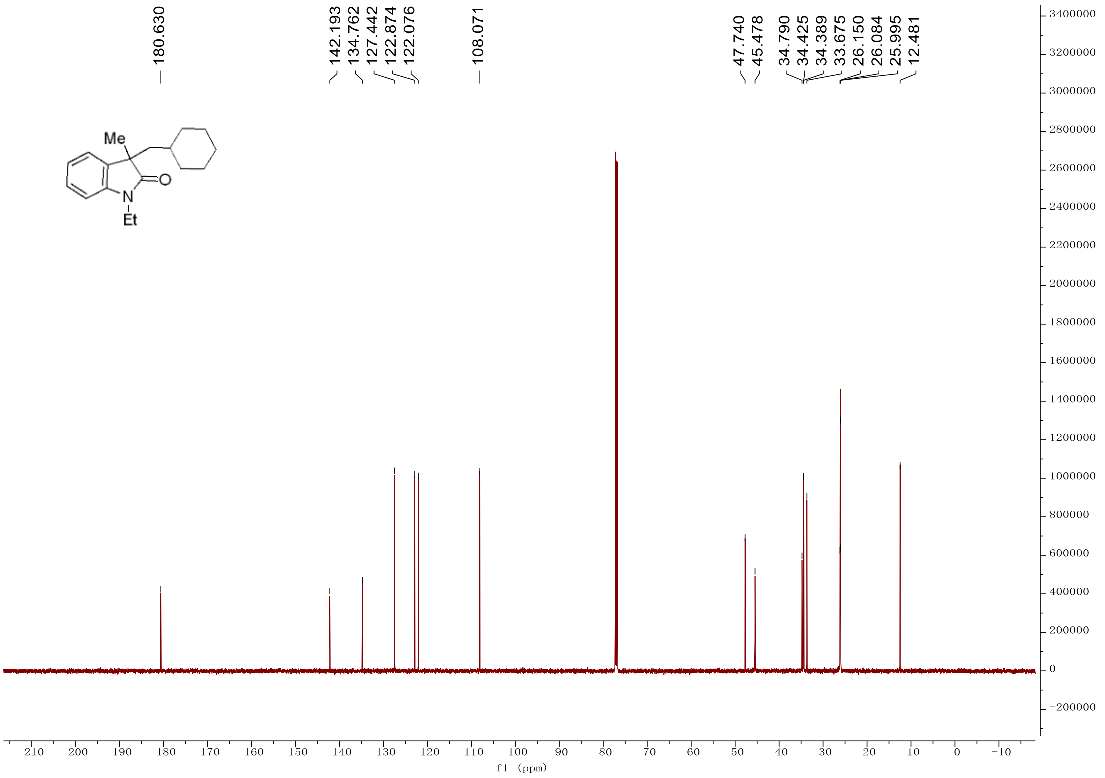

3na ^{1}H NMR (600 MHz, Chloroform-*d*)

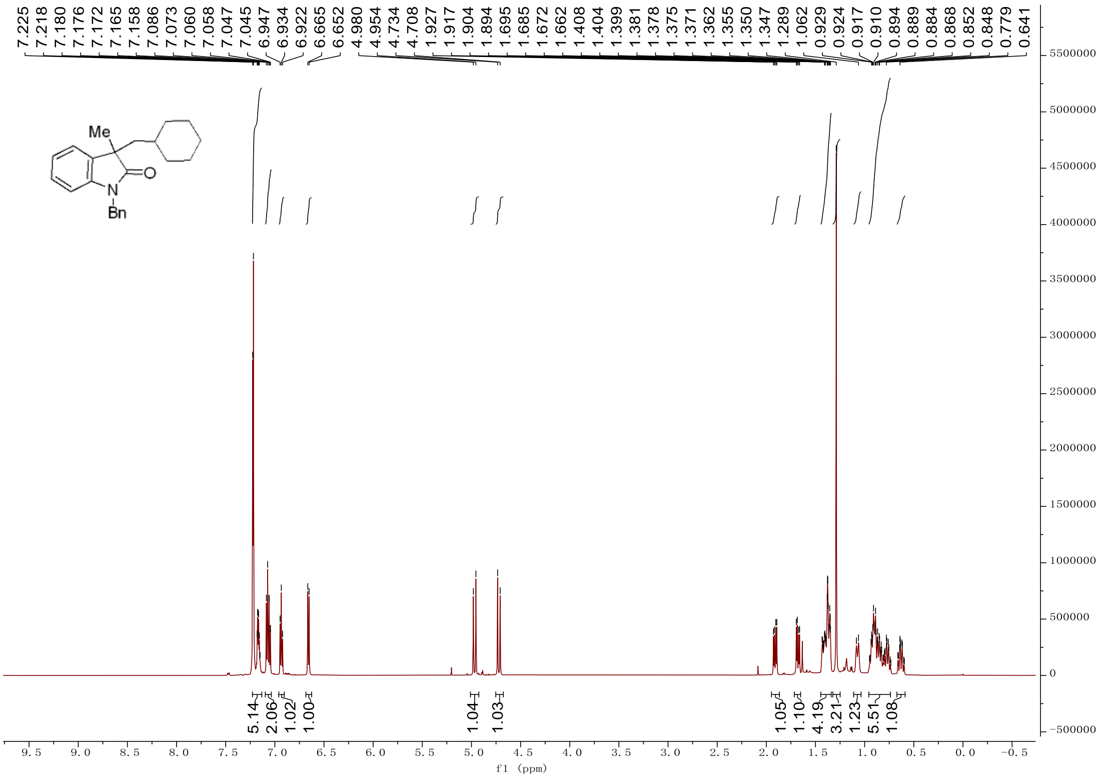

3na ^{13}C NMR (151 MHz, Chloroform-*d*)

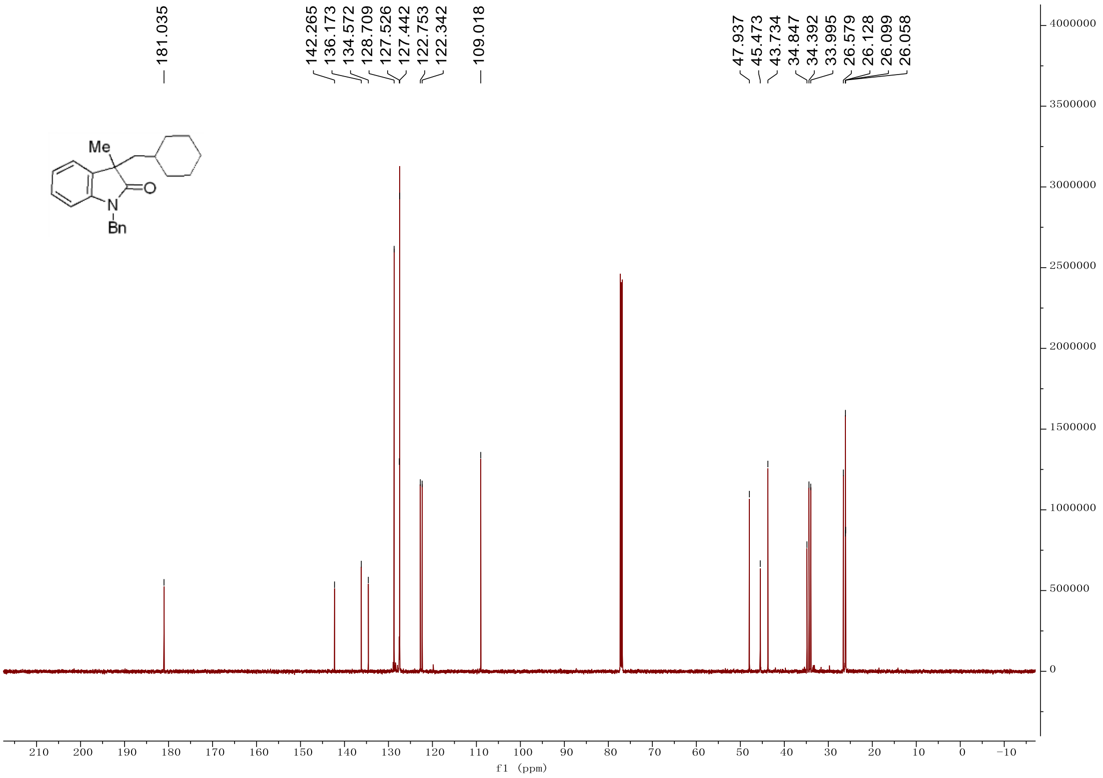

3oa ^{1}H NMR (600 MHz, Chloroform-*d*)

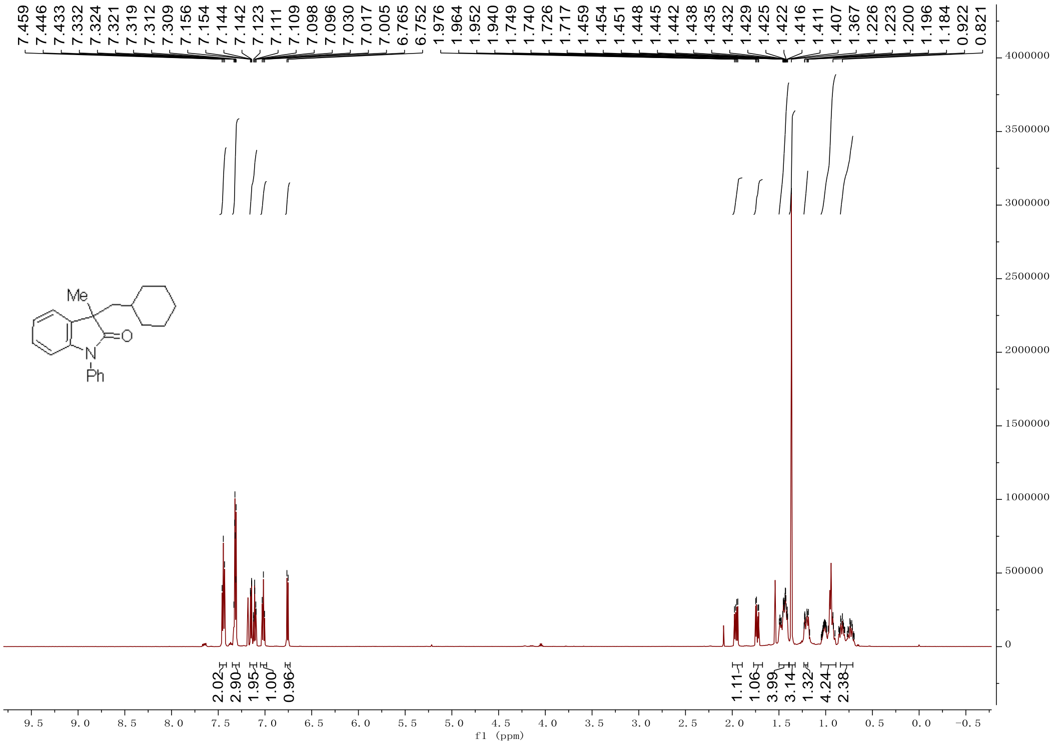

3oa ^{13}C NMR (151 MHz, Chloroform-*d*)

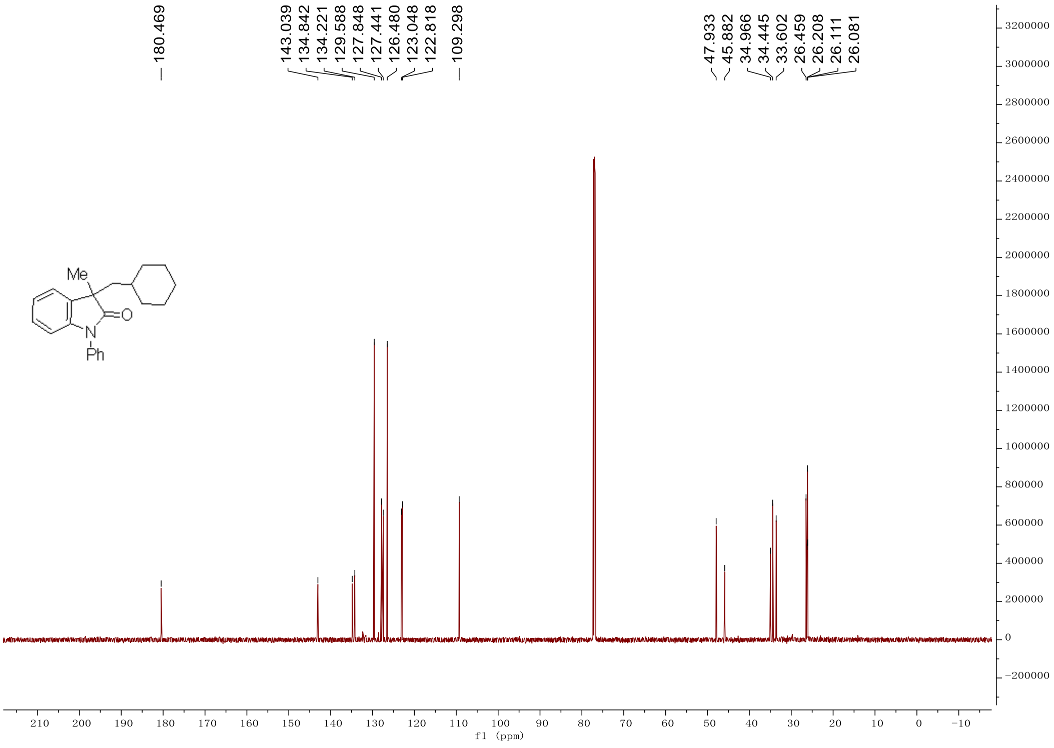

3pa ^{1}H NMR (600 MHz, Chloroform-*d*)

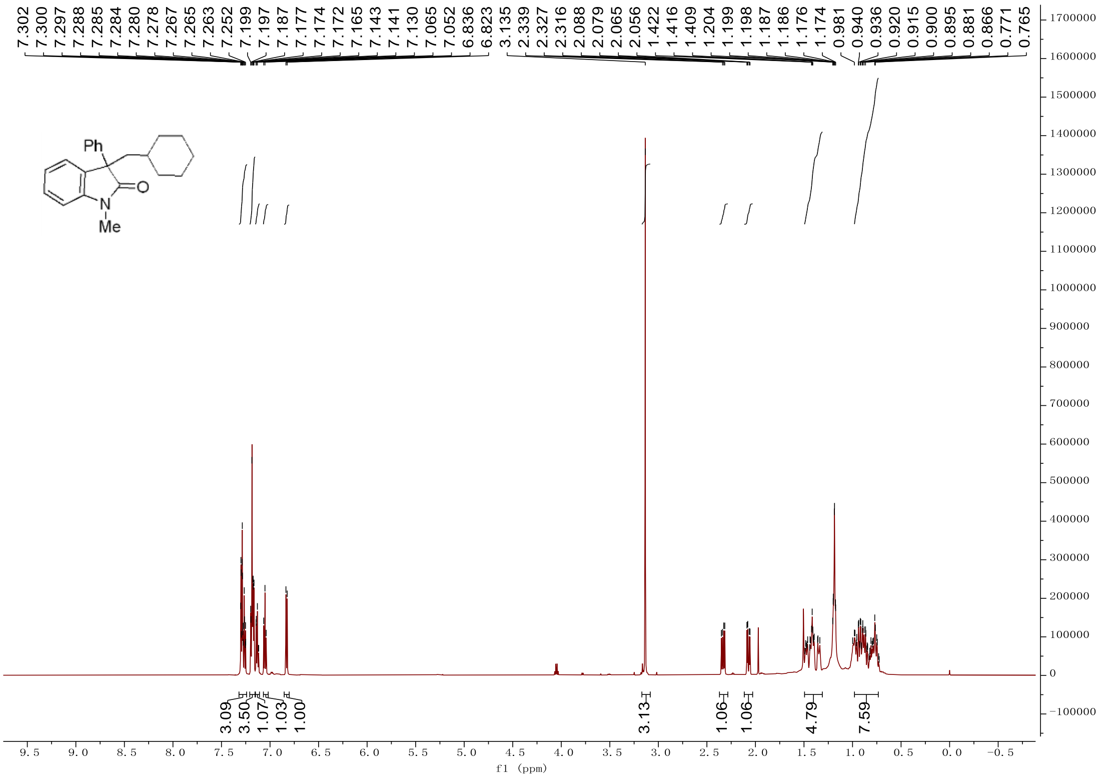

3pa ^{13}C NMR (151 MHz, Chloroform-*d*)

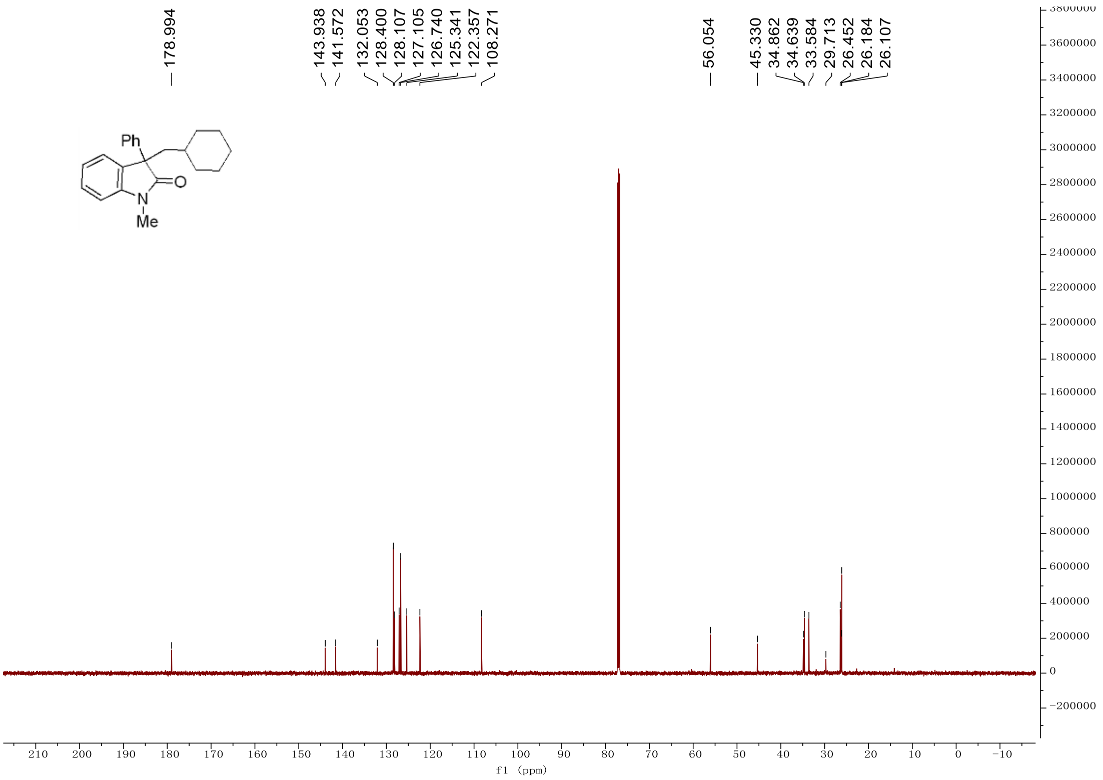

3qa ^{1}H NMR (600 MHz, Chloroform-*d*)

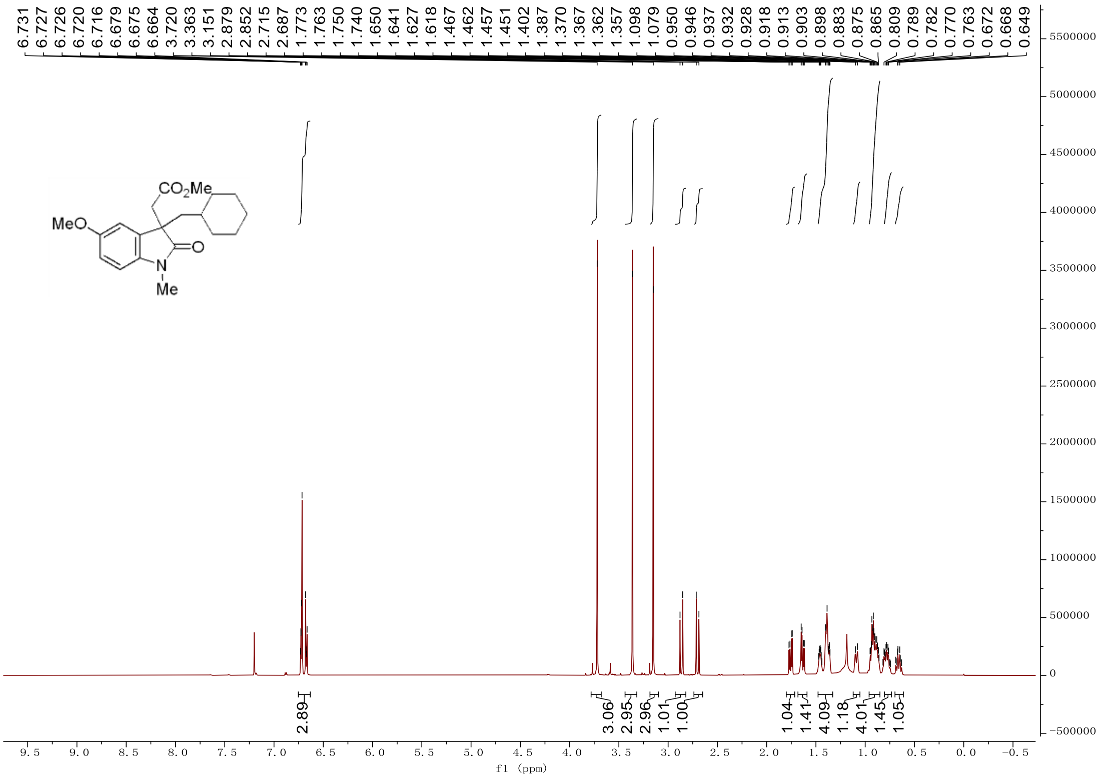

3qa ^{13}C NMR (151 MHz, Chloroform-*d*)

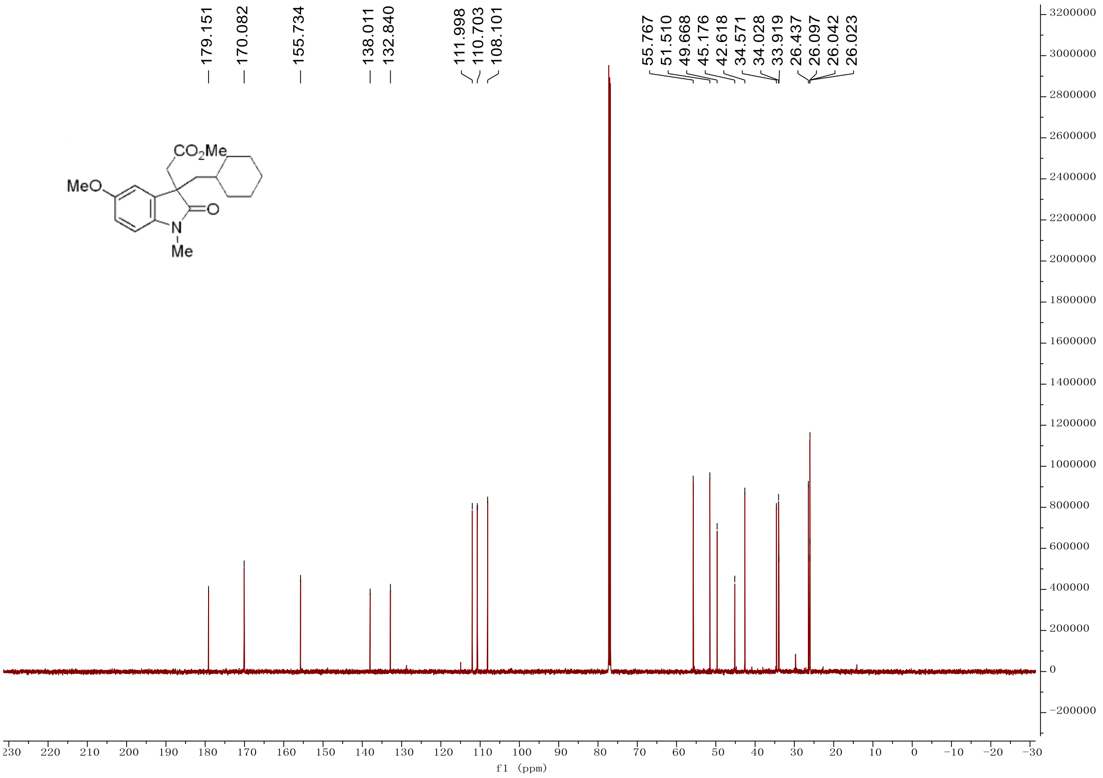

3ra ^{1}H NMR (600 MHz, Chloroform-*d*)

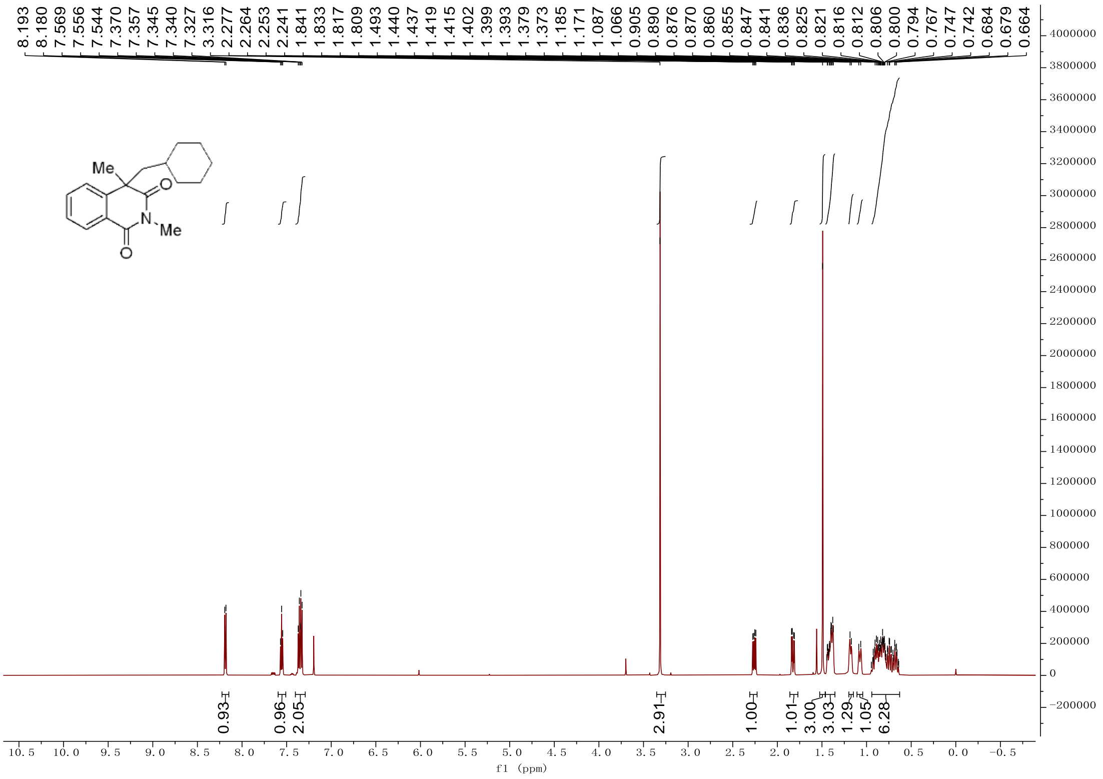

3ra ^{13}C NMR (151 MHz, Chloroform-*d*)

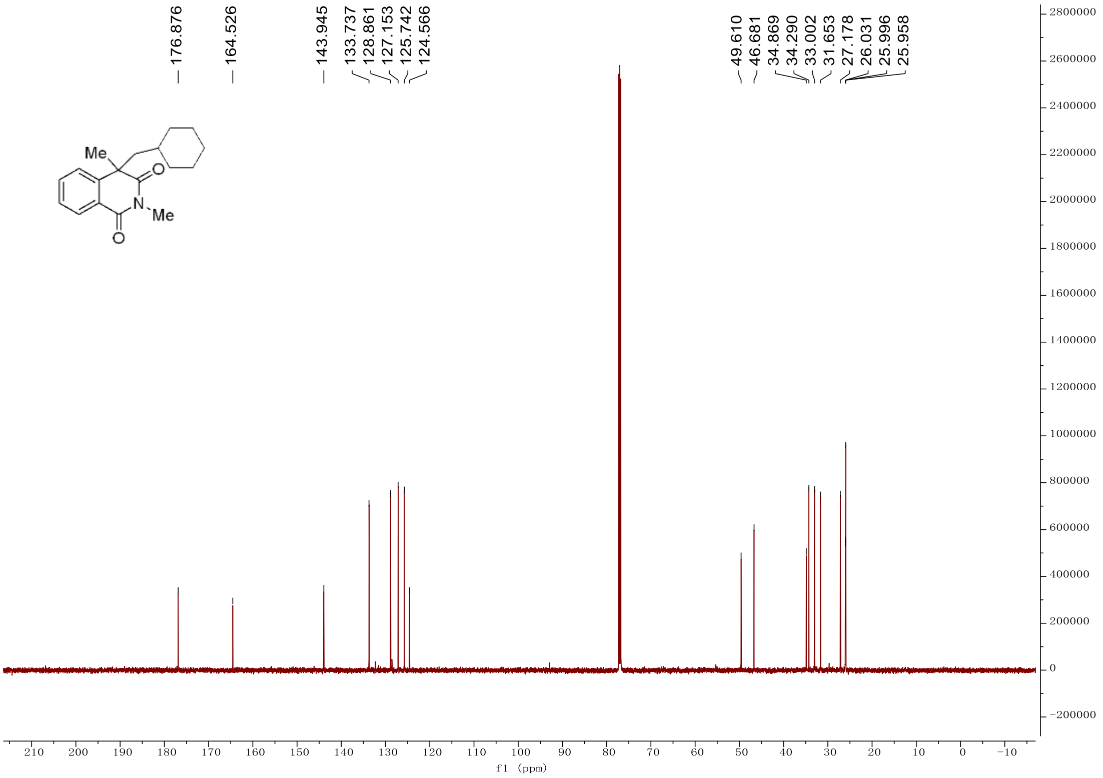

3ab ^{1}H NMR (600 MHz, Chloroform-*d*)

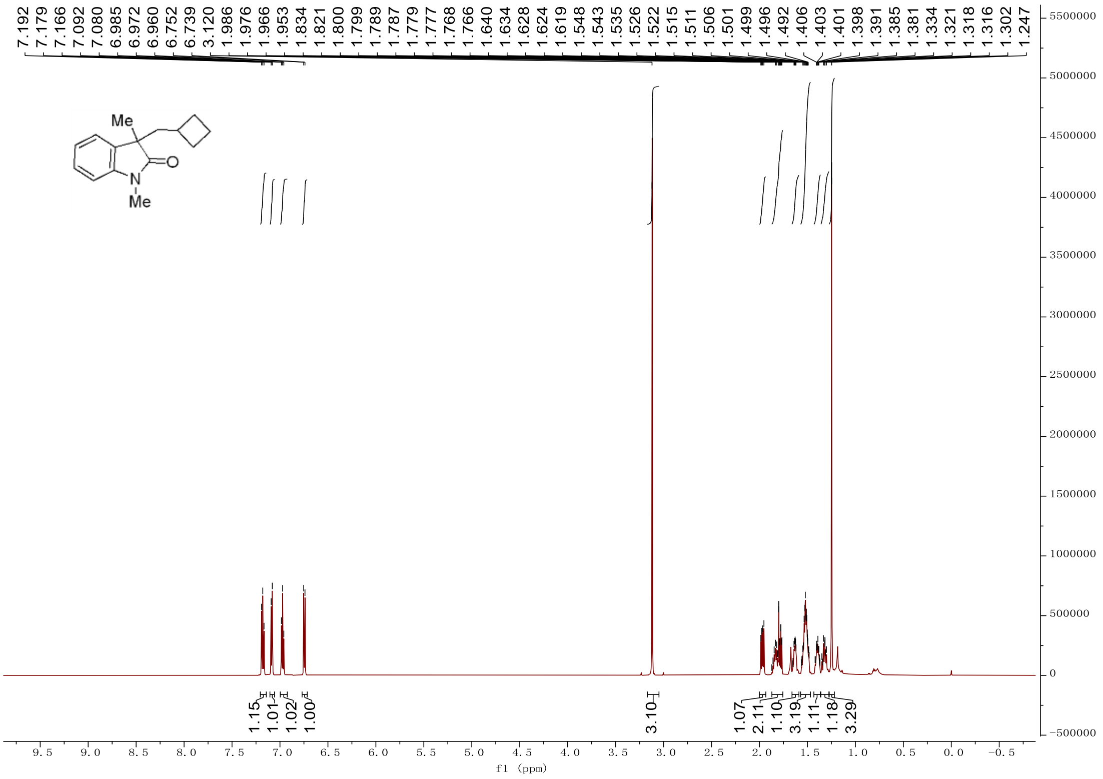

3ab ^{13}C NMR (151 MHz, Chloroform-*d*)

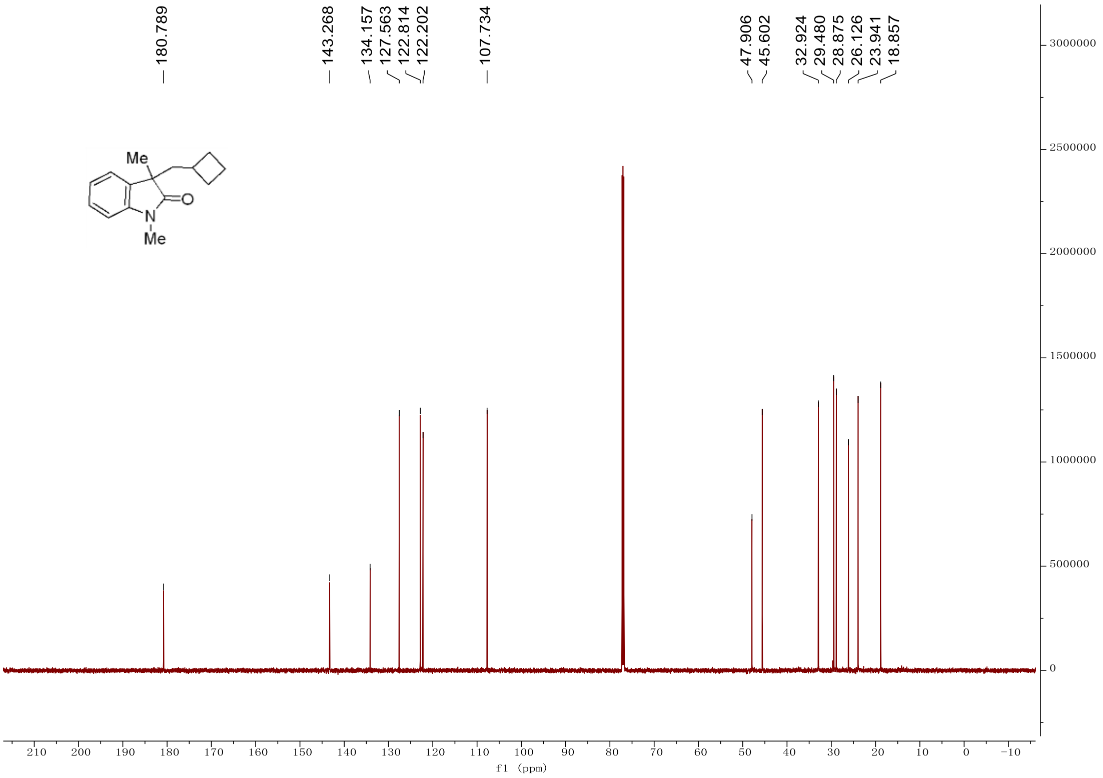

3ac ^{1}H NMR (600 MHz, Chloroform-*d*)

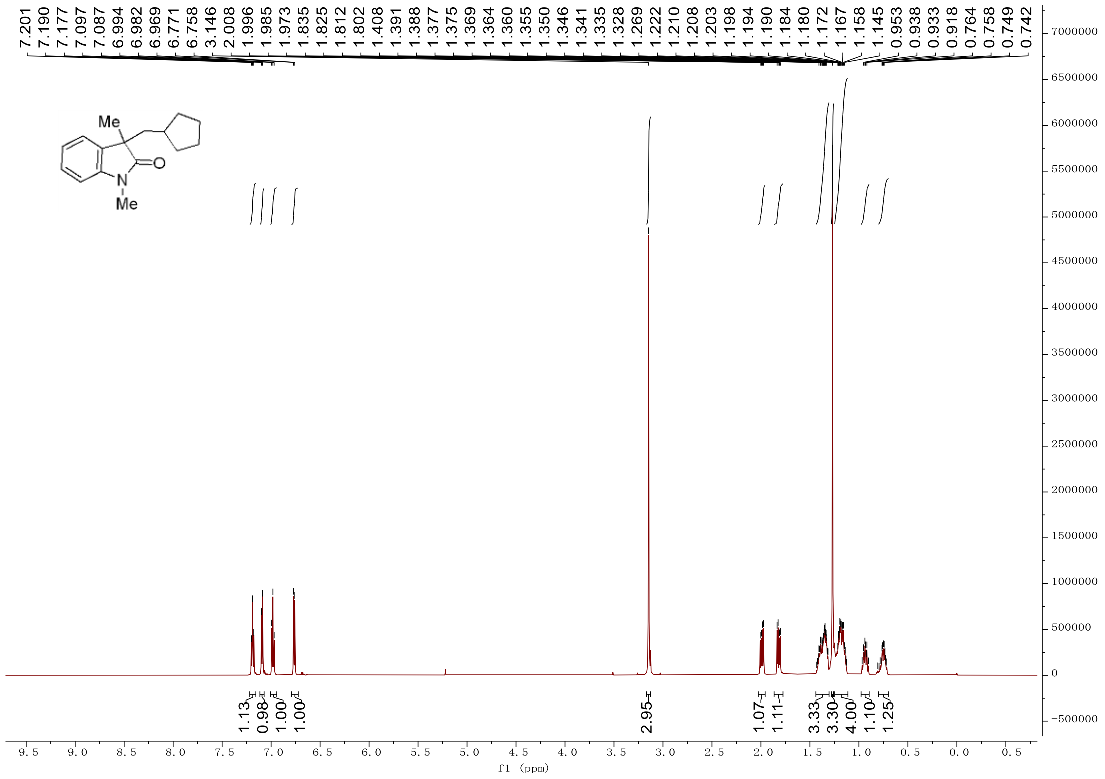

3ac ^{13}C NMR (151 MHz, Chloroform-*d*)

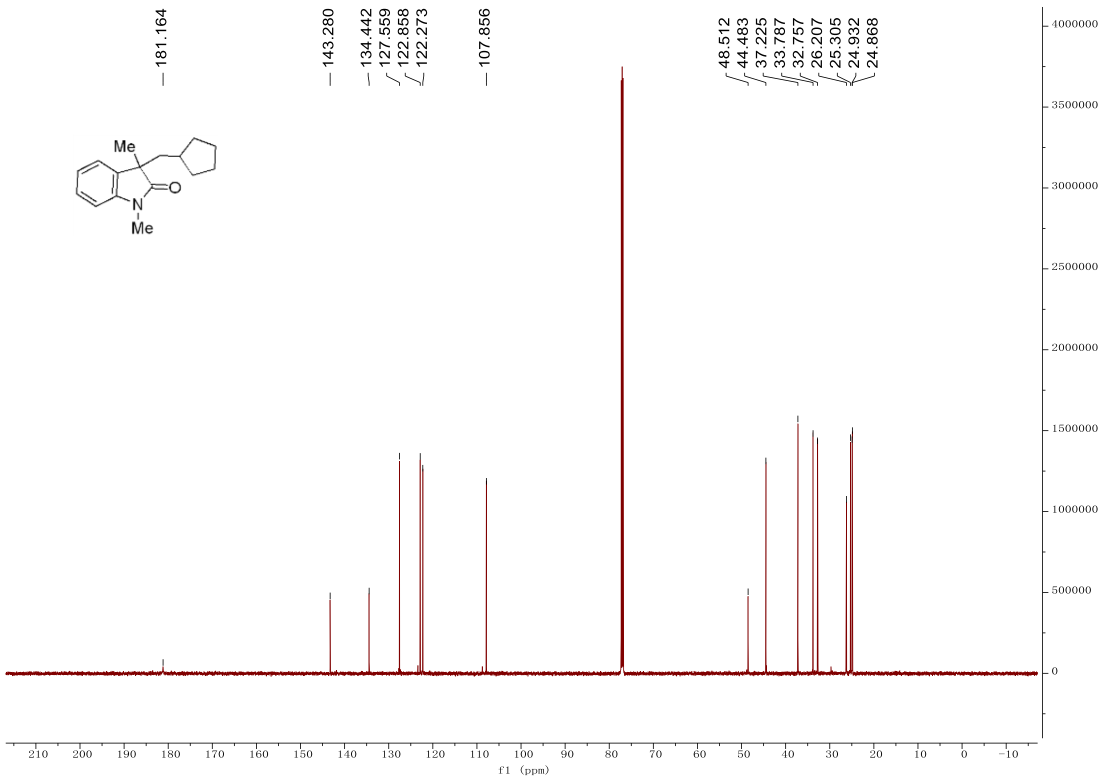

3ad ^{1}H NMR (600 MHz, Chloroform-*d*)

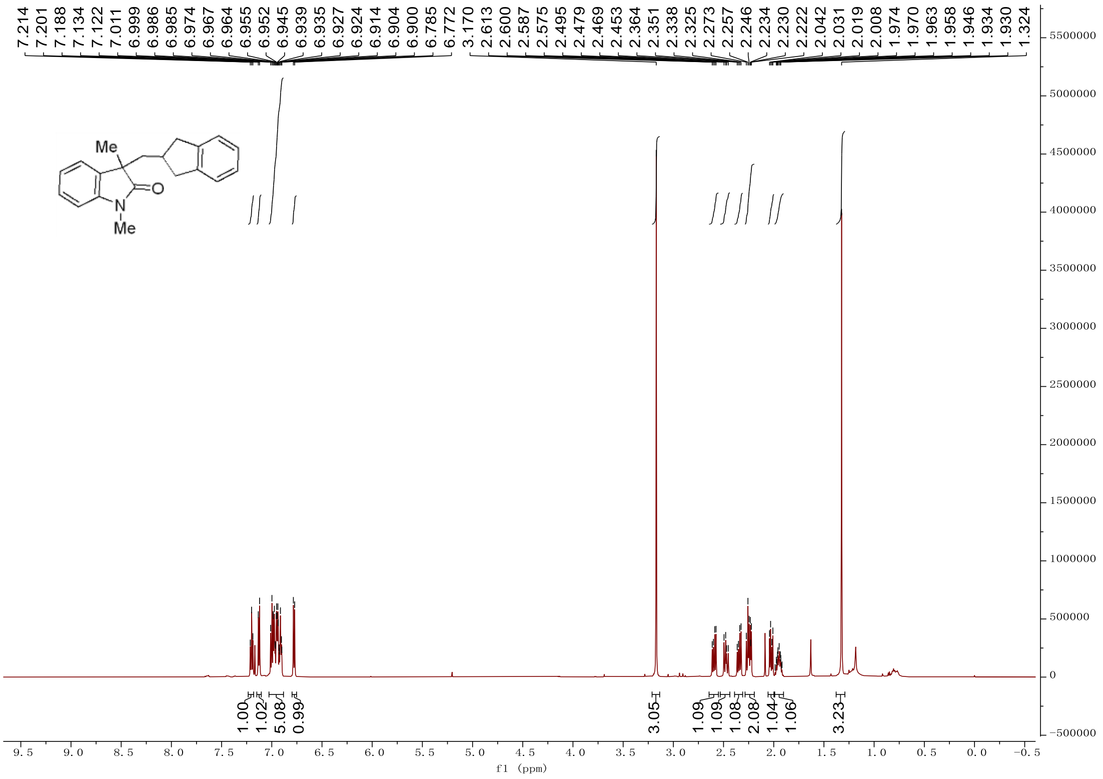

3ad ^{13}C NMR (151 MHz, Chloroform-*d*)

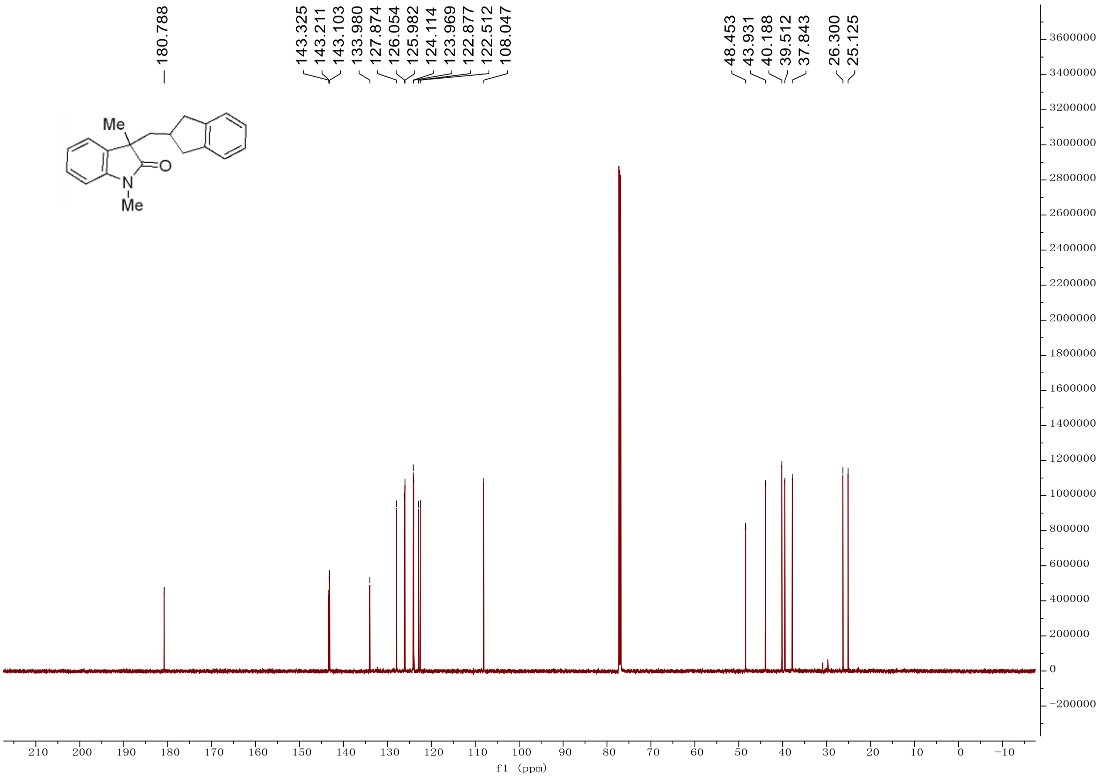

3ae ^{1}H NMR (600 MHz, Chloroform-*d*)

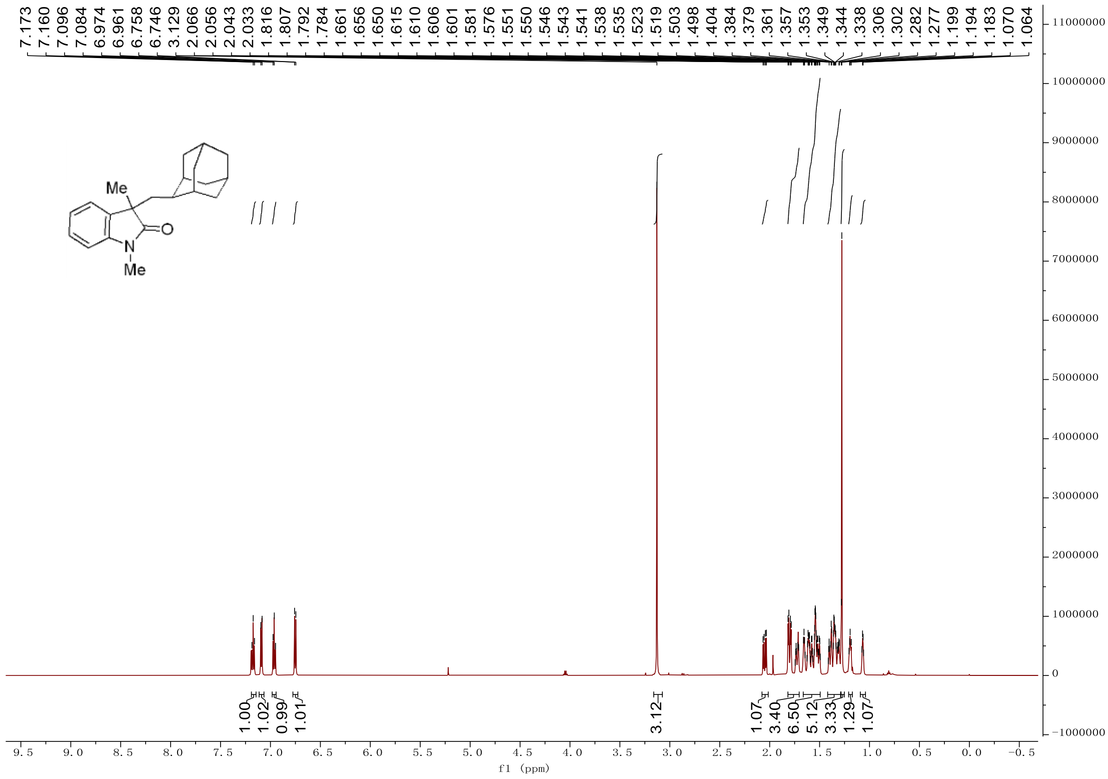

3ae ^{13}C NMR (151 MHz, Chloroform-*d*)

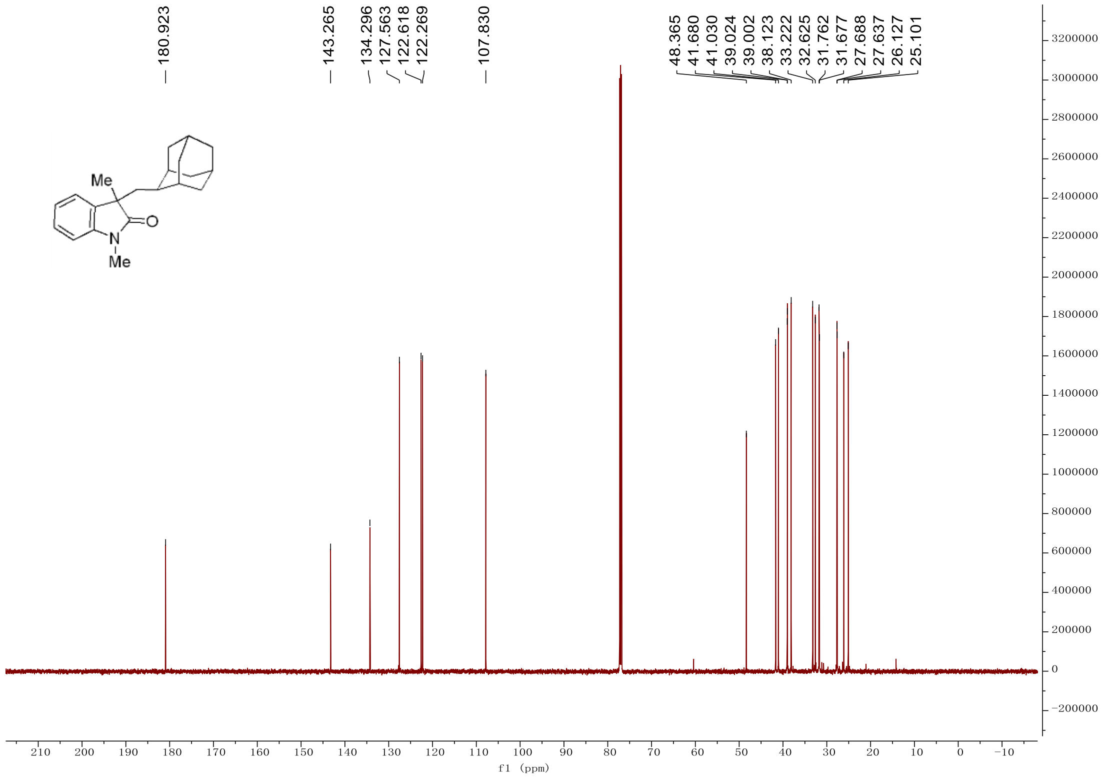

3af ^{1}H NMR (600 MHz, Chloroform-*d*)

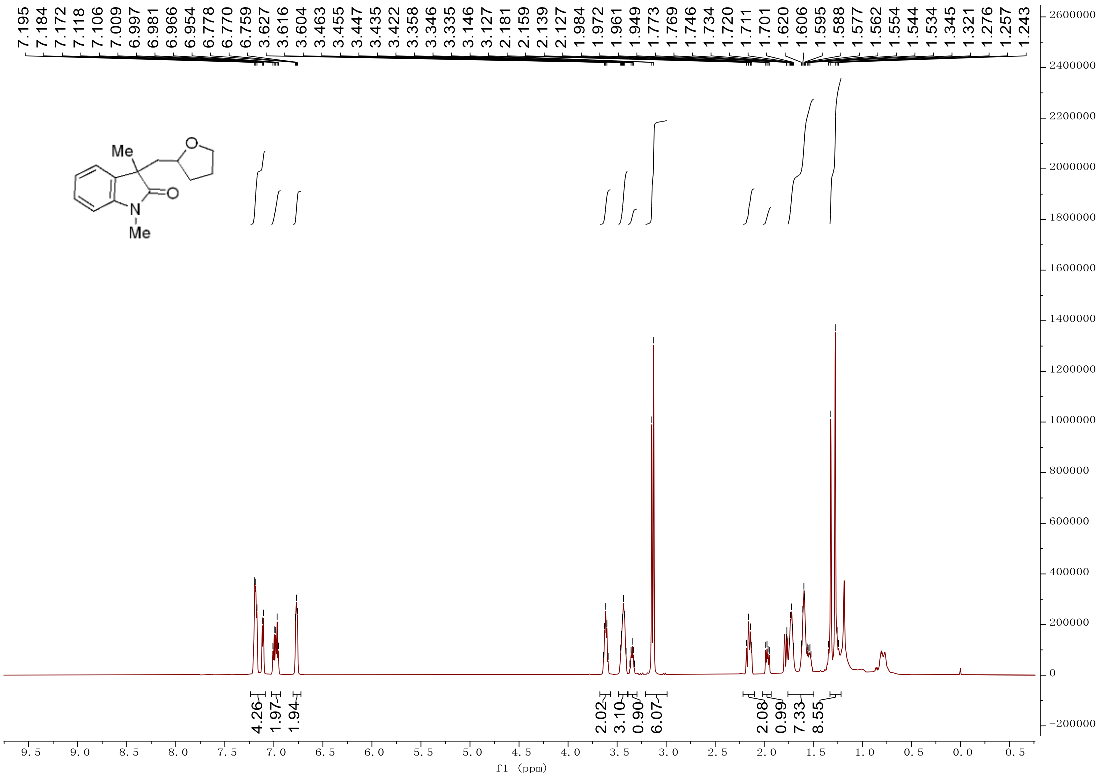

3af ^{13}C NMR (151 MHz, Chloroform-*d*)

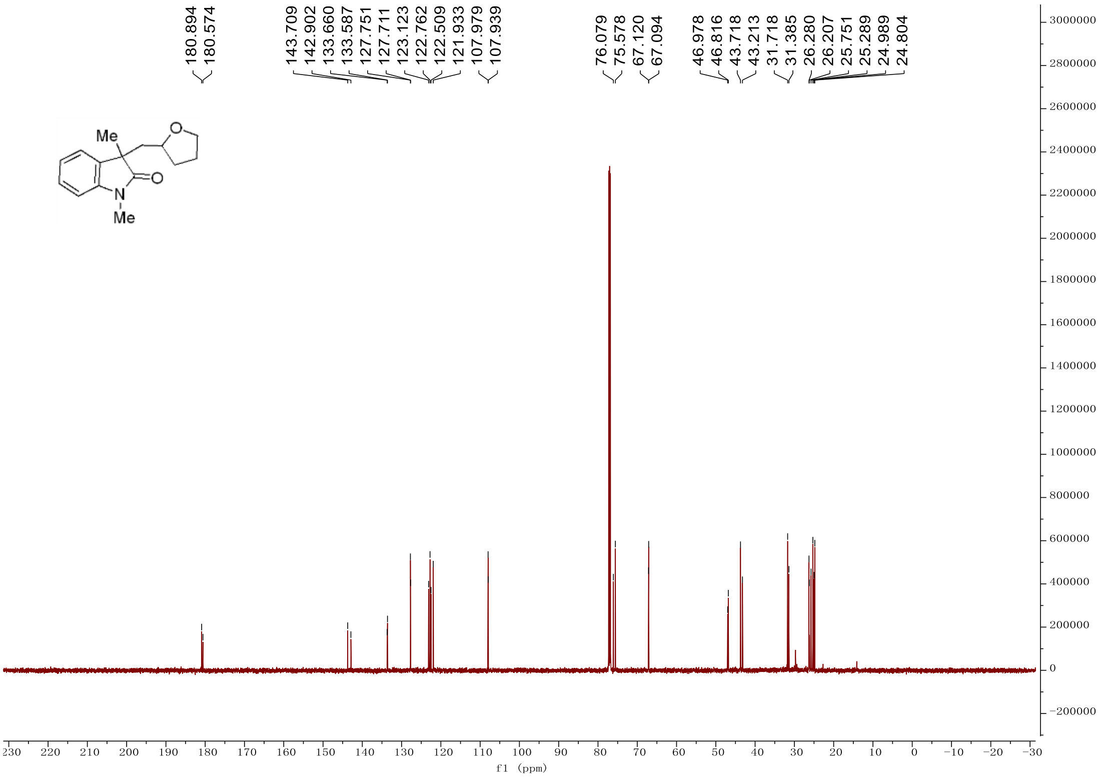

3ag ^{1}H NMR 600 MHz, Chloroform-*d*)

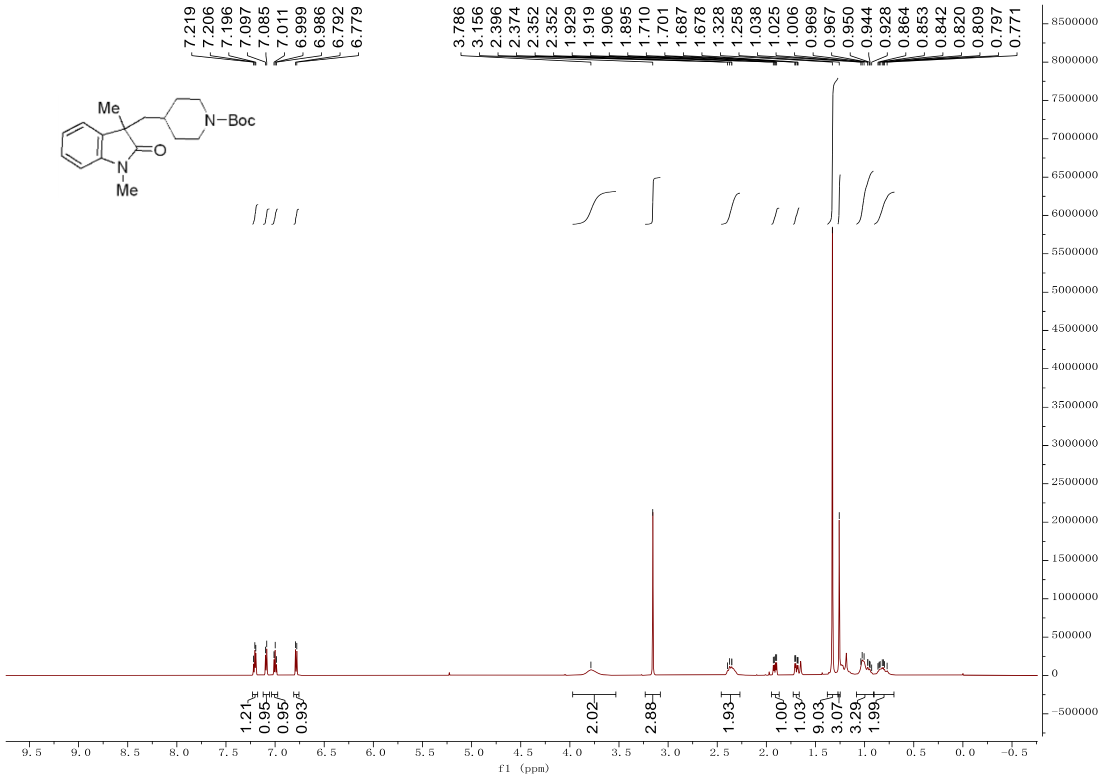

3ag ^{13}C NMR (151 MHz, Chloroform-*d*)

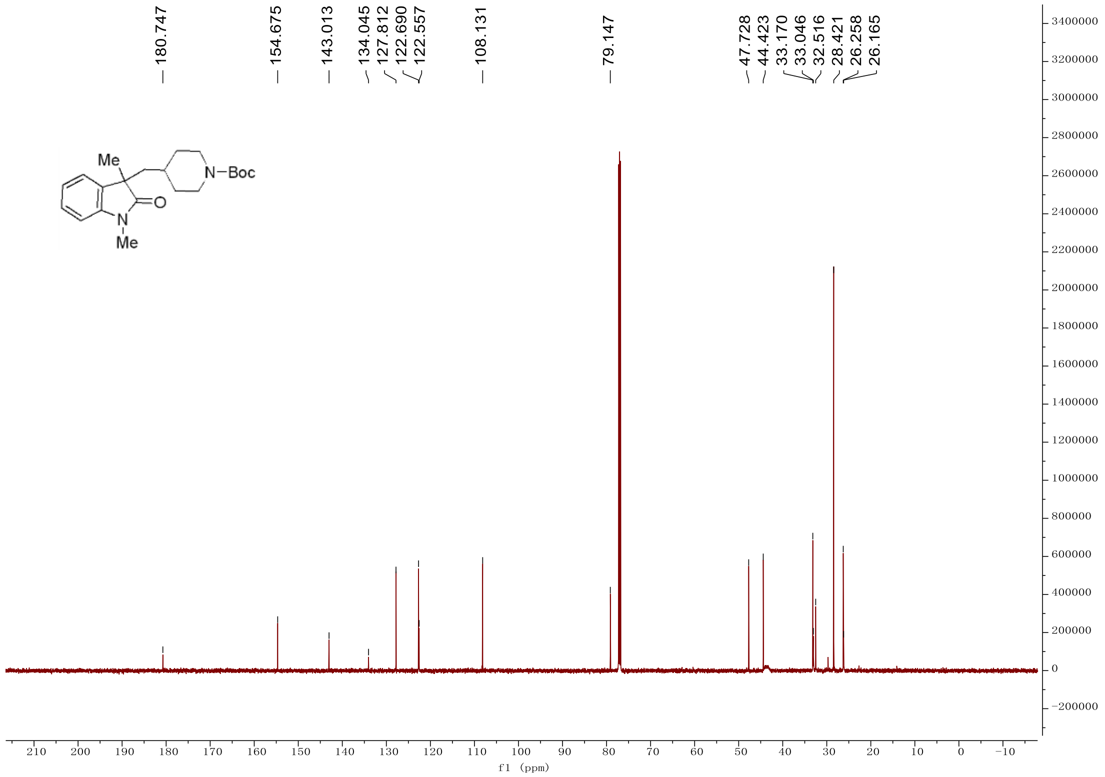

3ah ^{1}H NMR (600 MHz, Chloroform-*d*)

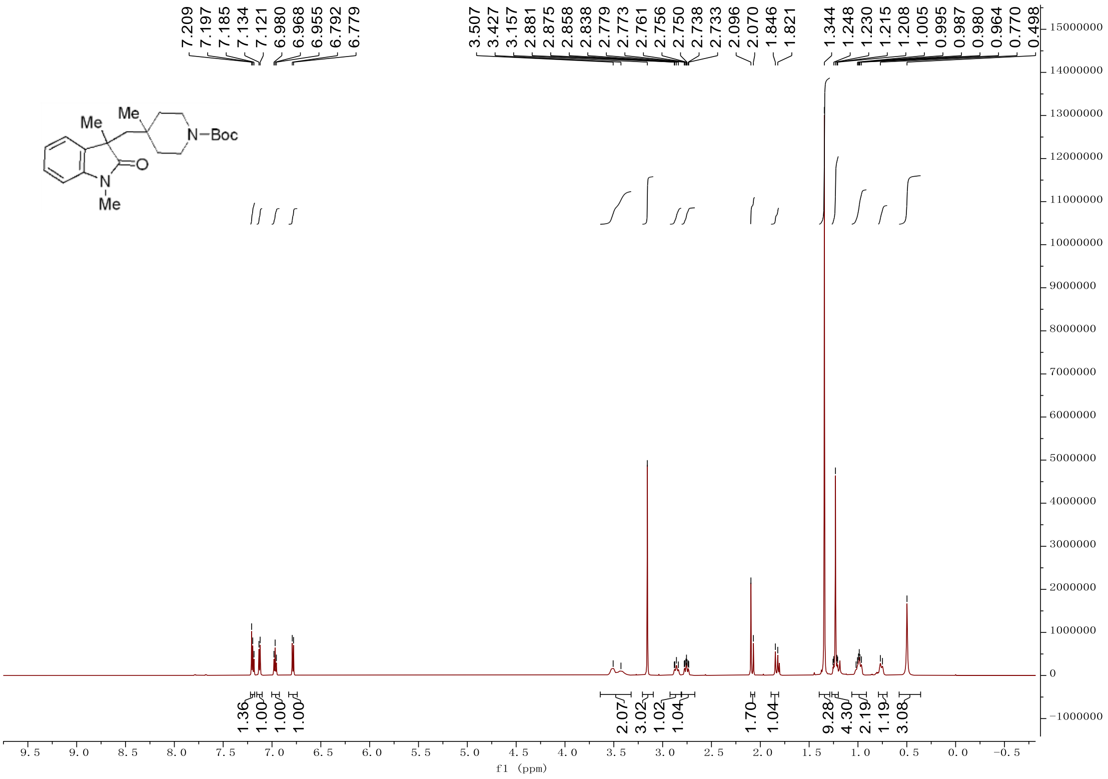

3ah ^{13}C NMR (151 MHz, Chloroform-*d*)

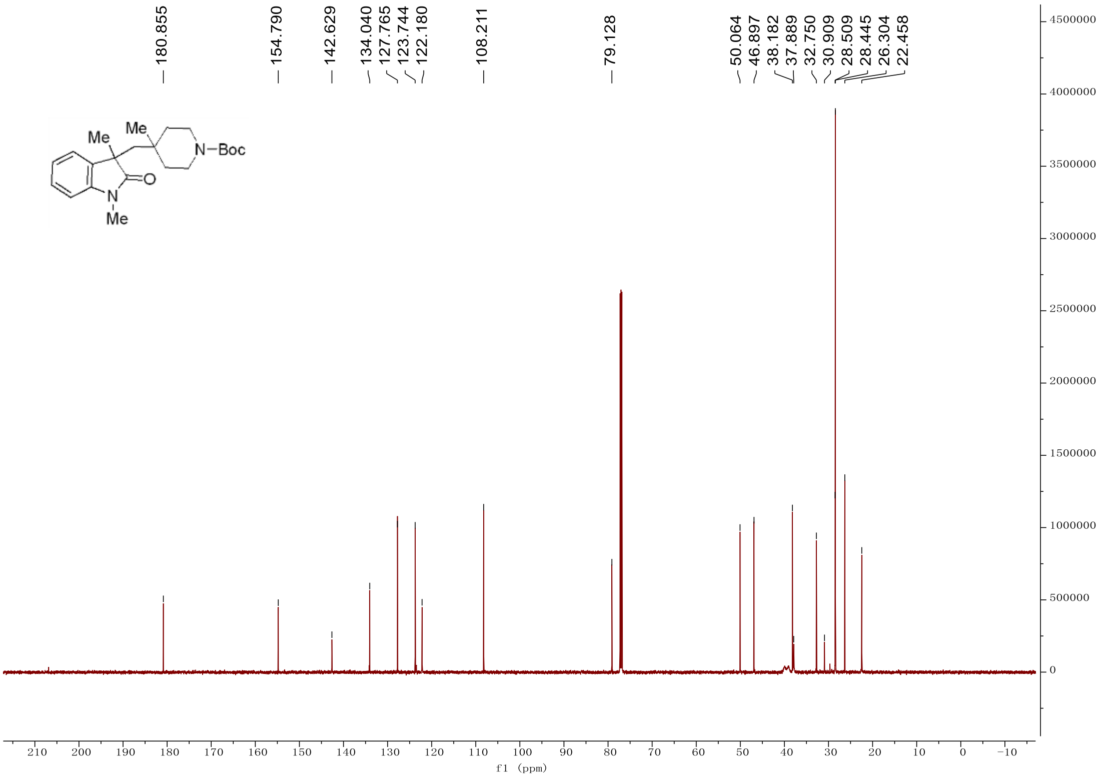

3ai ^{1}H NMR (600 MHz, Chloroform-*d*)

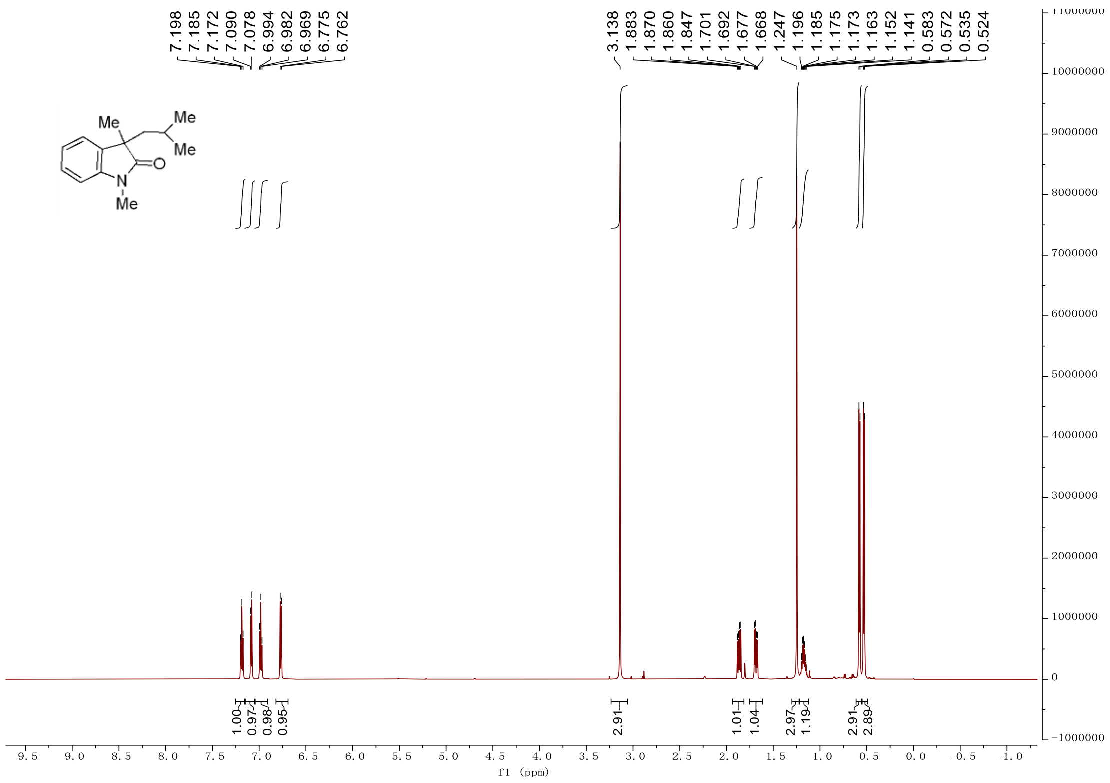

3ai ^{13}C NMR (151 MHz, Chloroform-*d*)

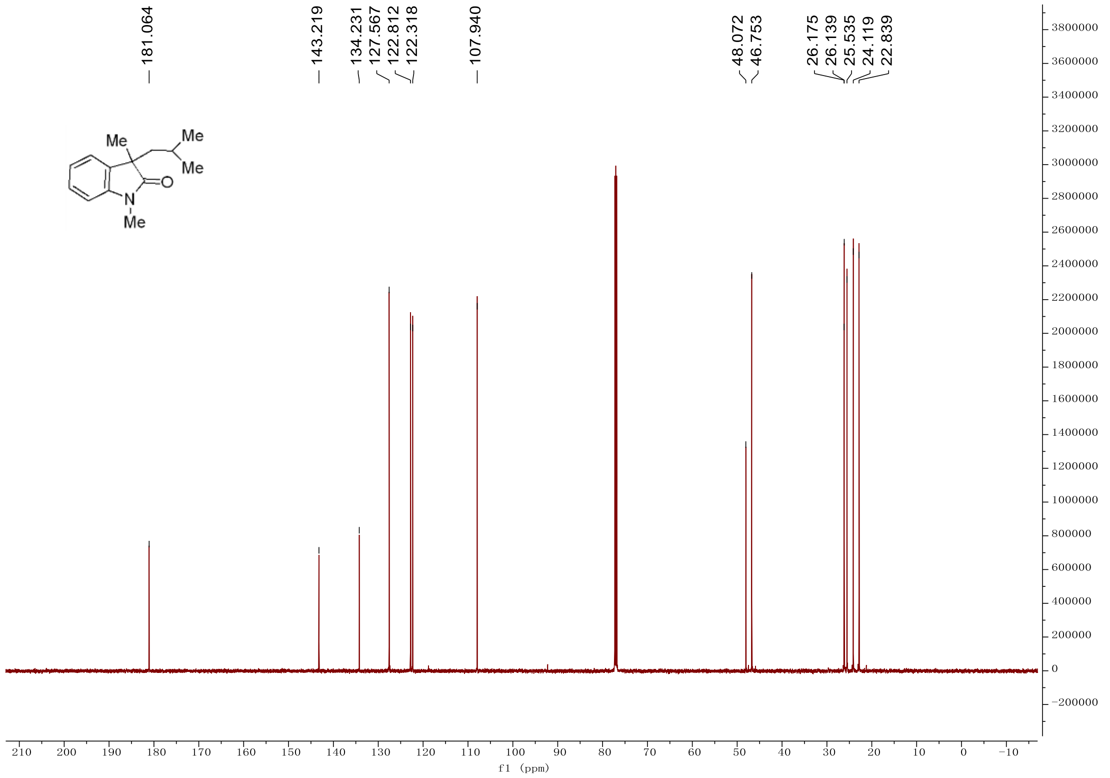

3aj ^{1}H NMR (600 MHz, Chloroform-*d*)

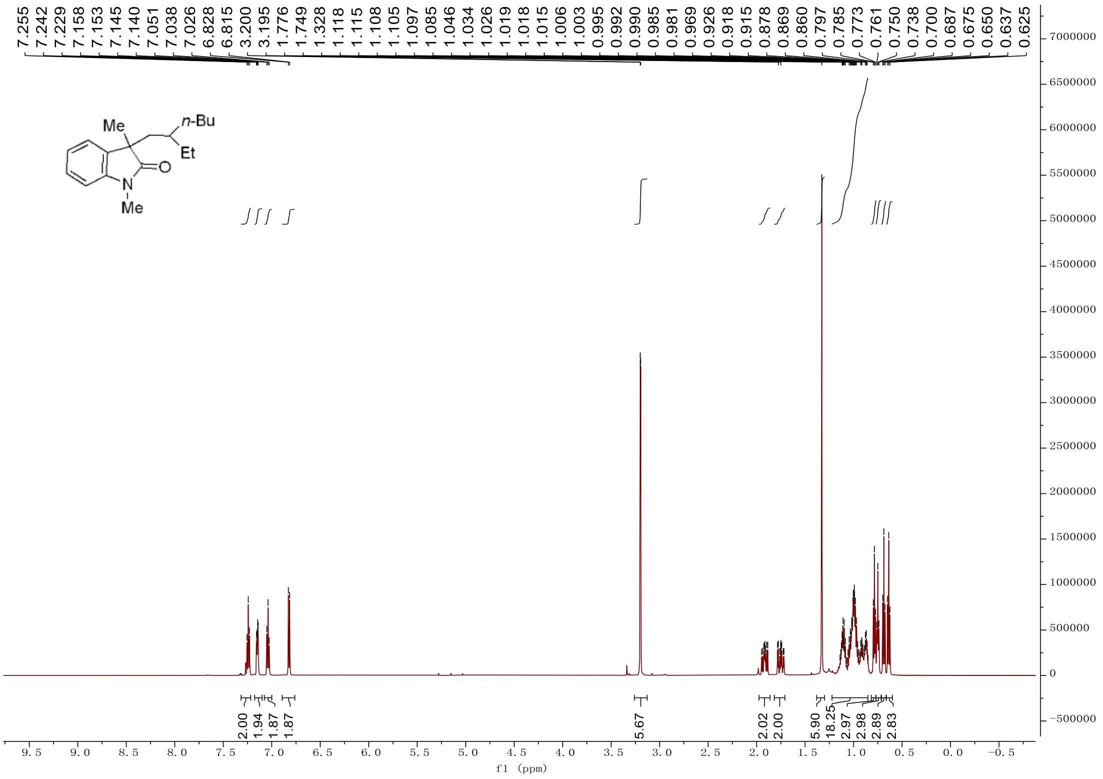

3aj ^{13}C NMR (151 MHz, Chloroform-*d*)

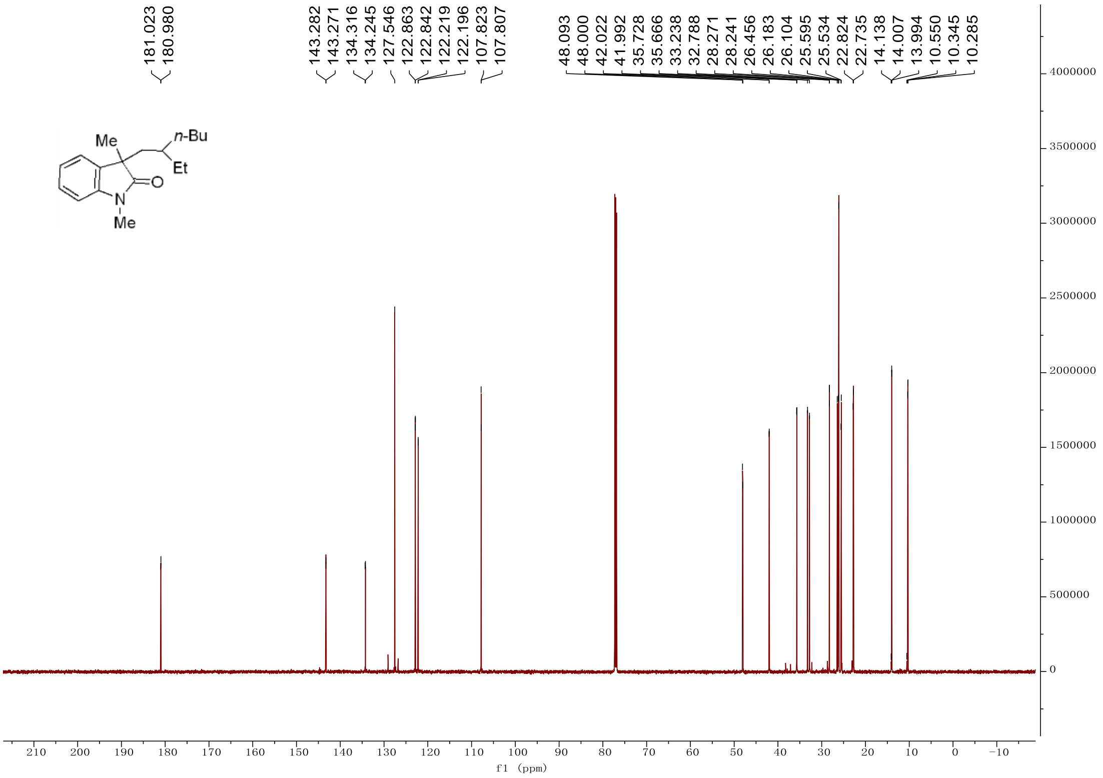

3ak ^{1}H NMR (600 MHz, Chloroform-d)

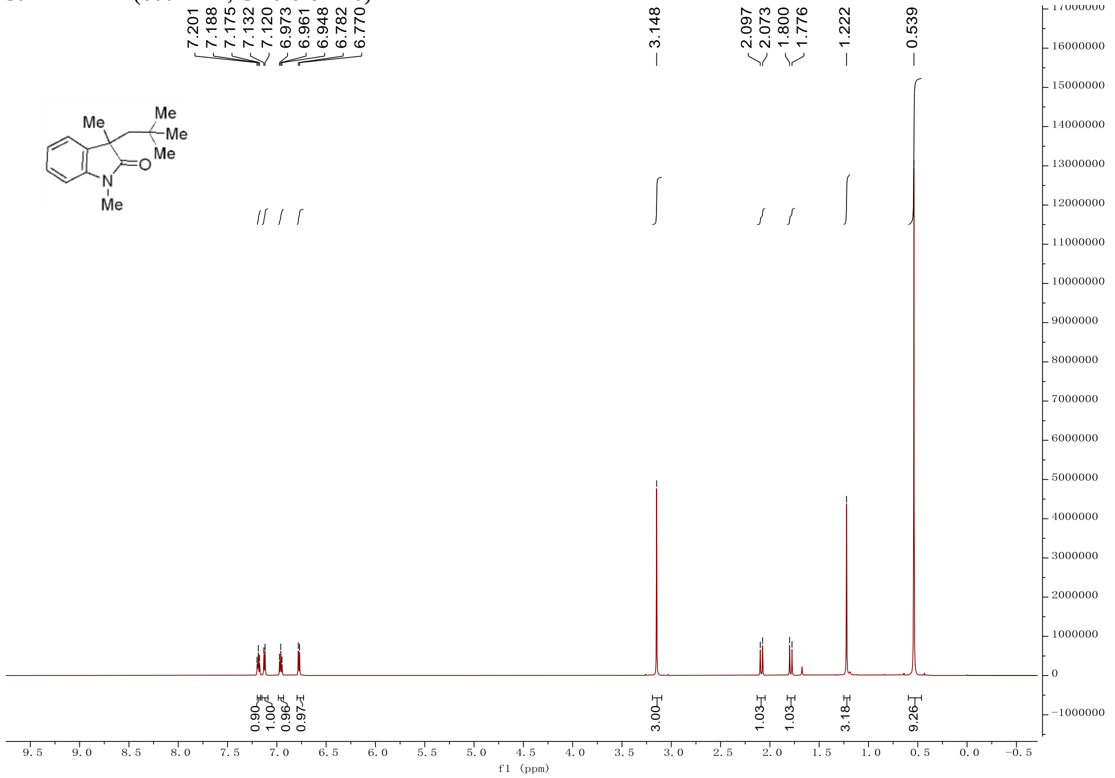

3ak ^{13}C NMR (151 MHz, Chloroform-d)

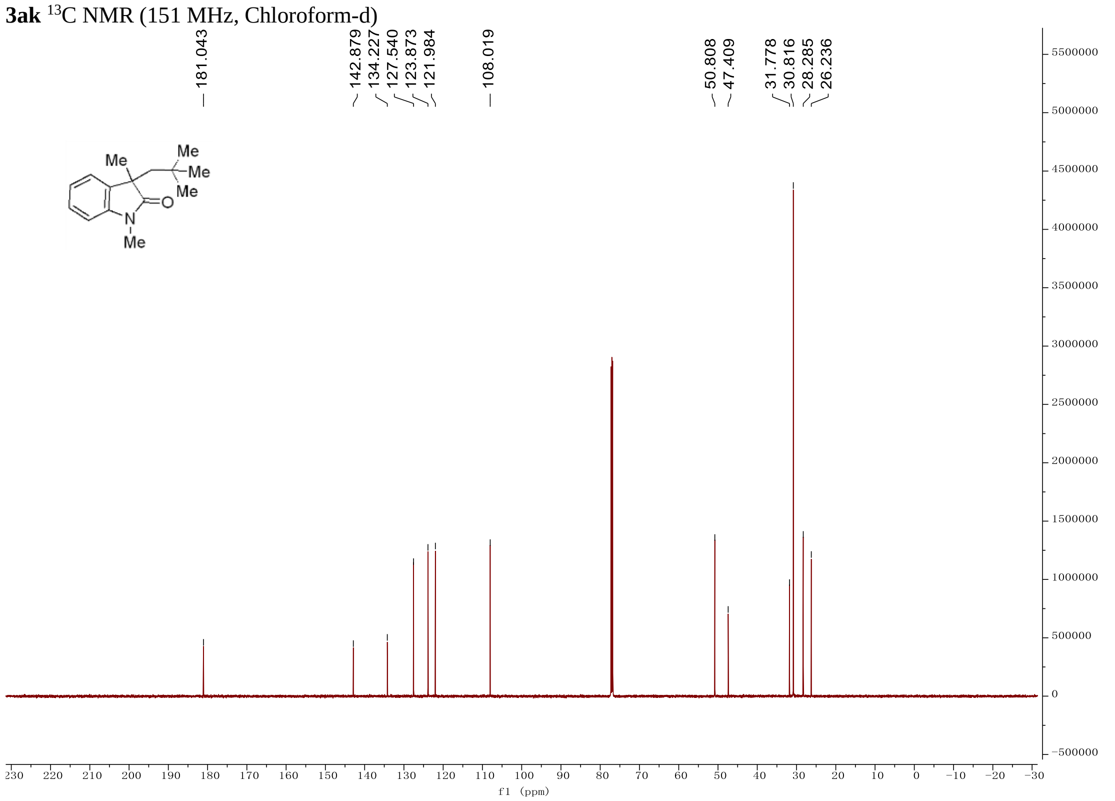

3al ^{1}H NMR (600 MHz, Chloroform-*d*)

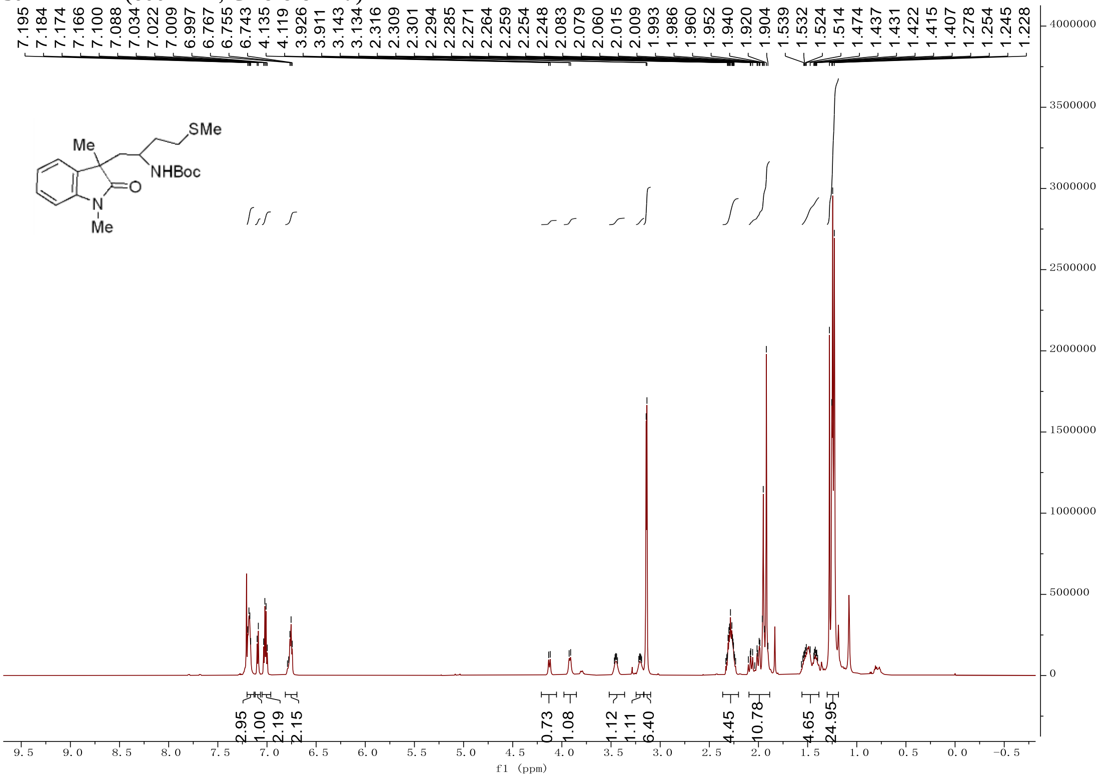

3al ^{13}C NMR (151 MHz, Chloroform-*d*)

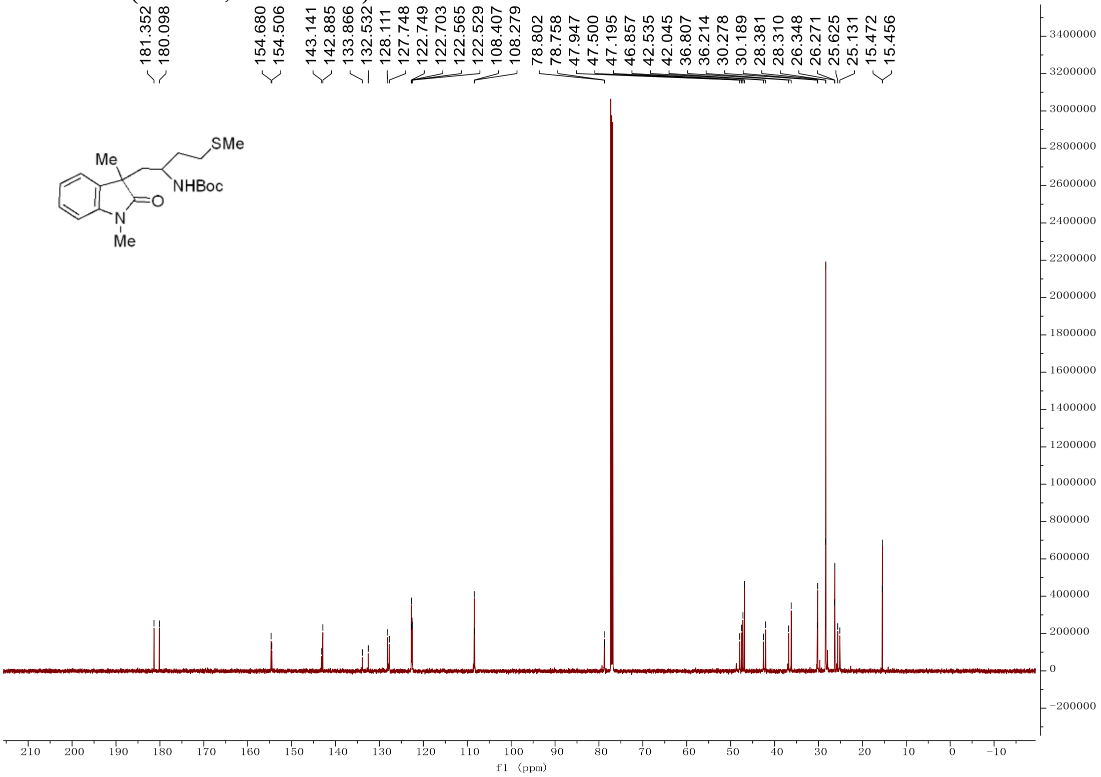

3am ^{1}H NMR (600 MHz, Chloroform-*d*)

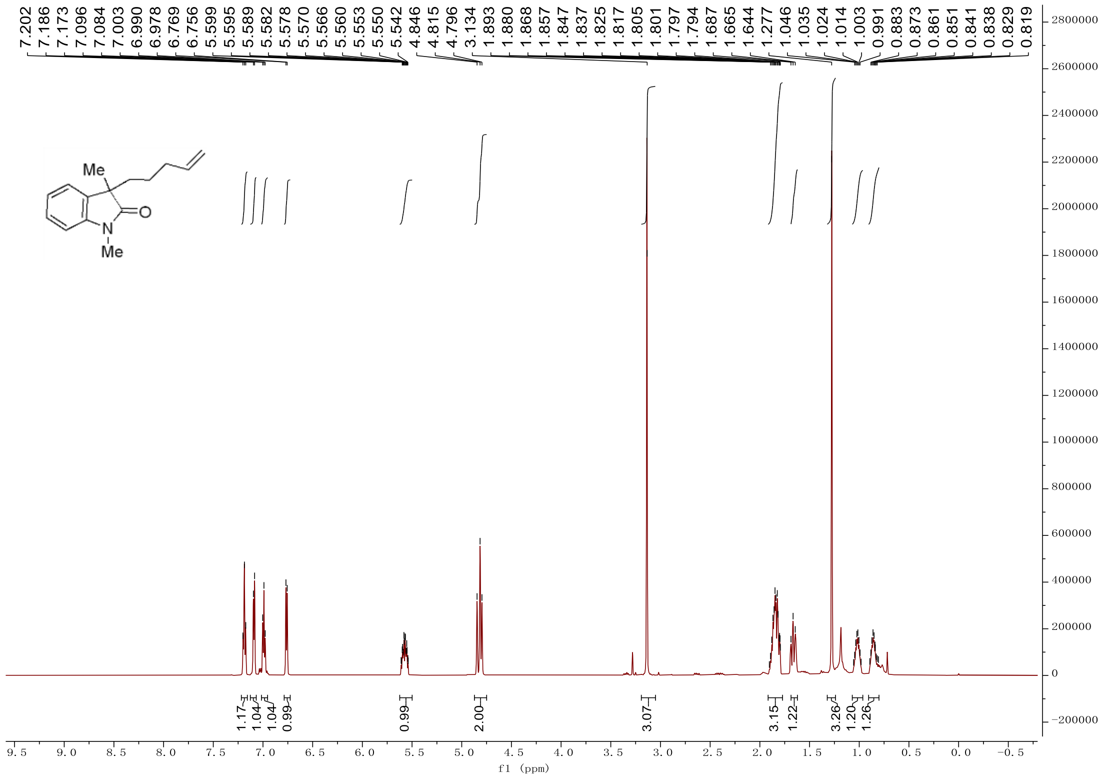

3am ^{13}C NMR (151 MHz, Chloroform-*d*)

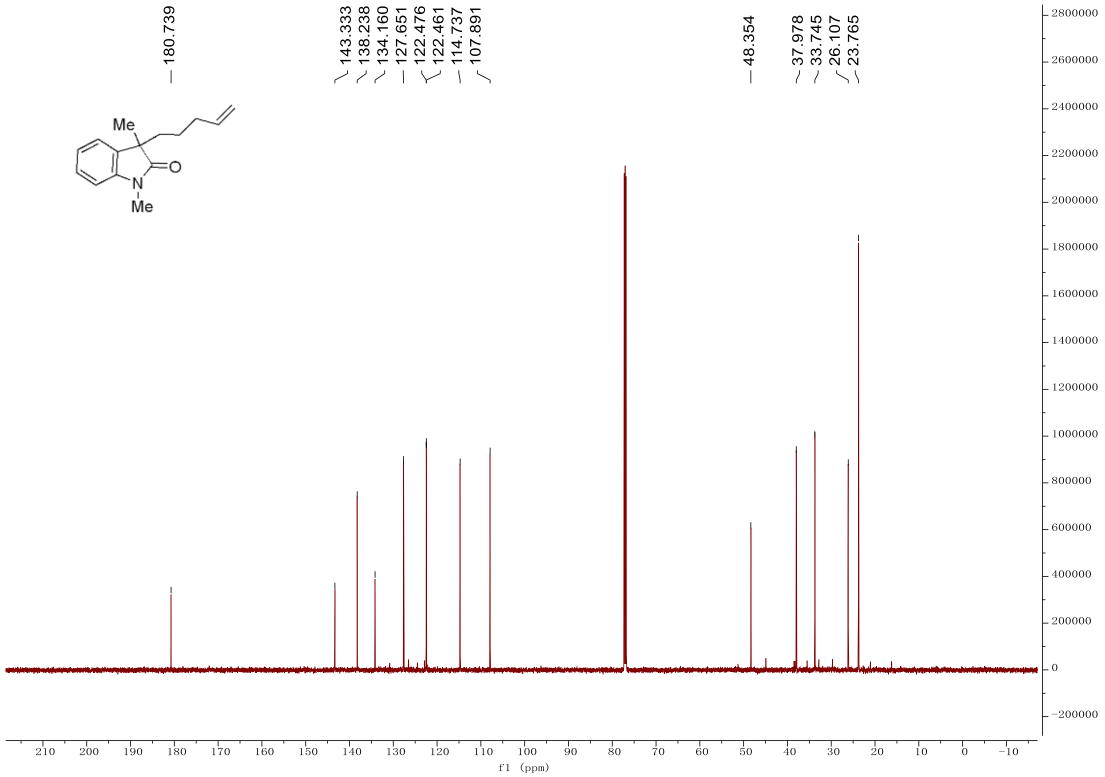

3an ^{1}H NMR 600 MHz, Chloroform-*d*)

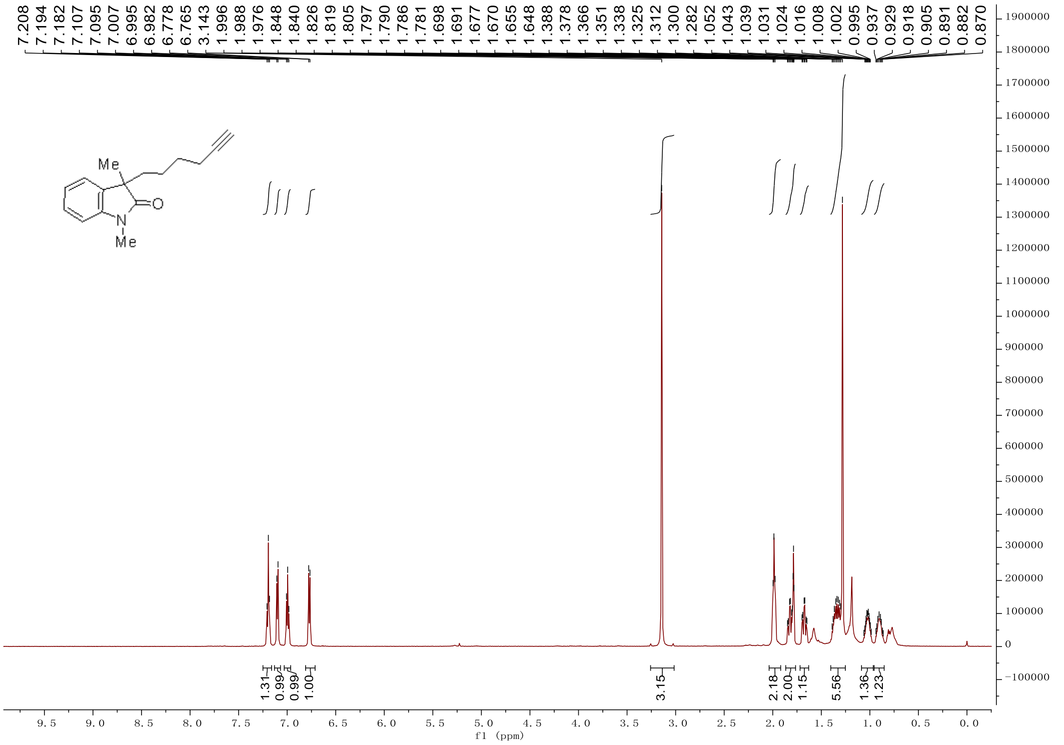

3an ^{13}C NMR (151 MHz, Chloroform-*d*)

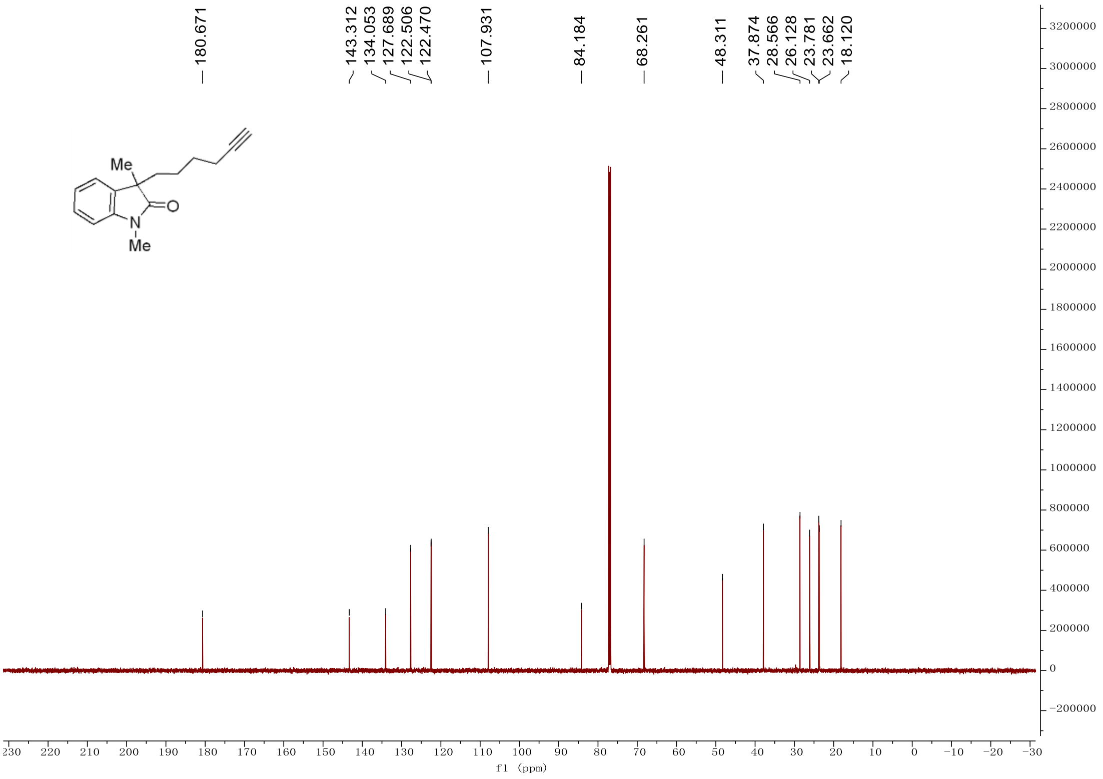

3ao ^{1}H NMR (600 MHz, Chloroform-*d*)

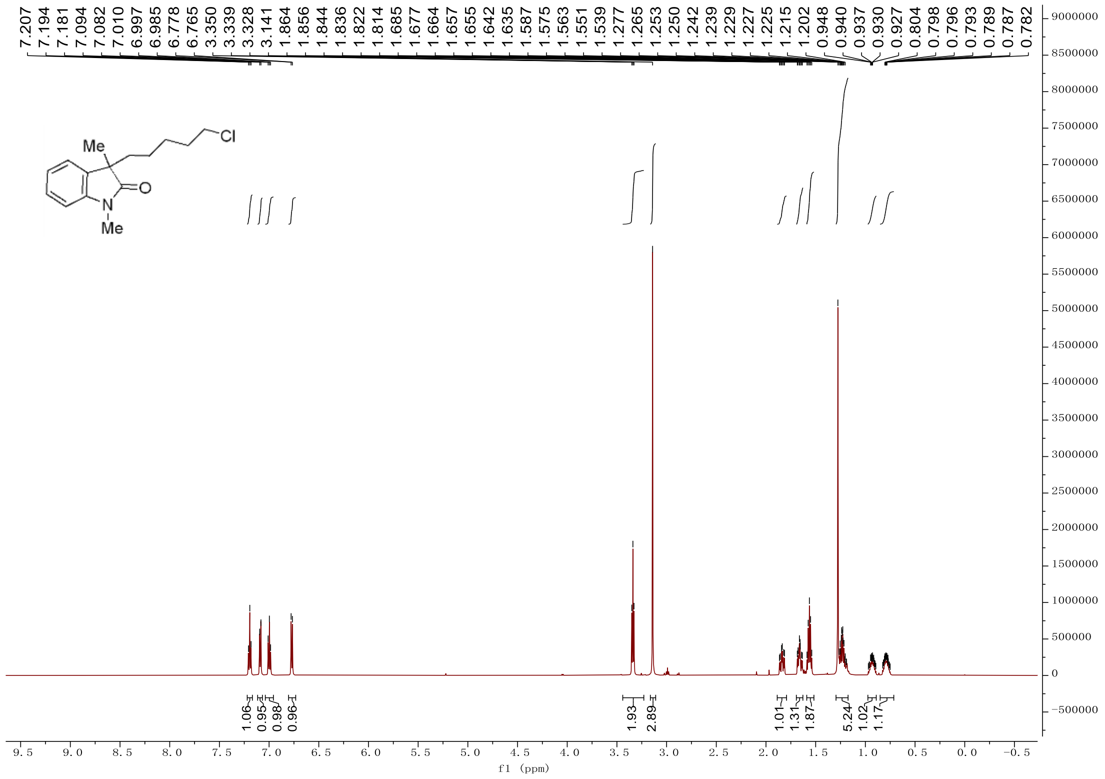

3ao ^{13}C NMR (151 MHz, Chloroform-*d*)

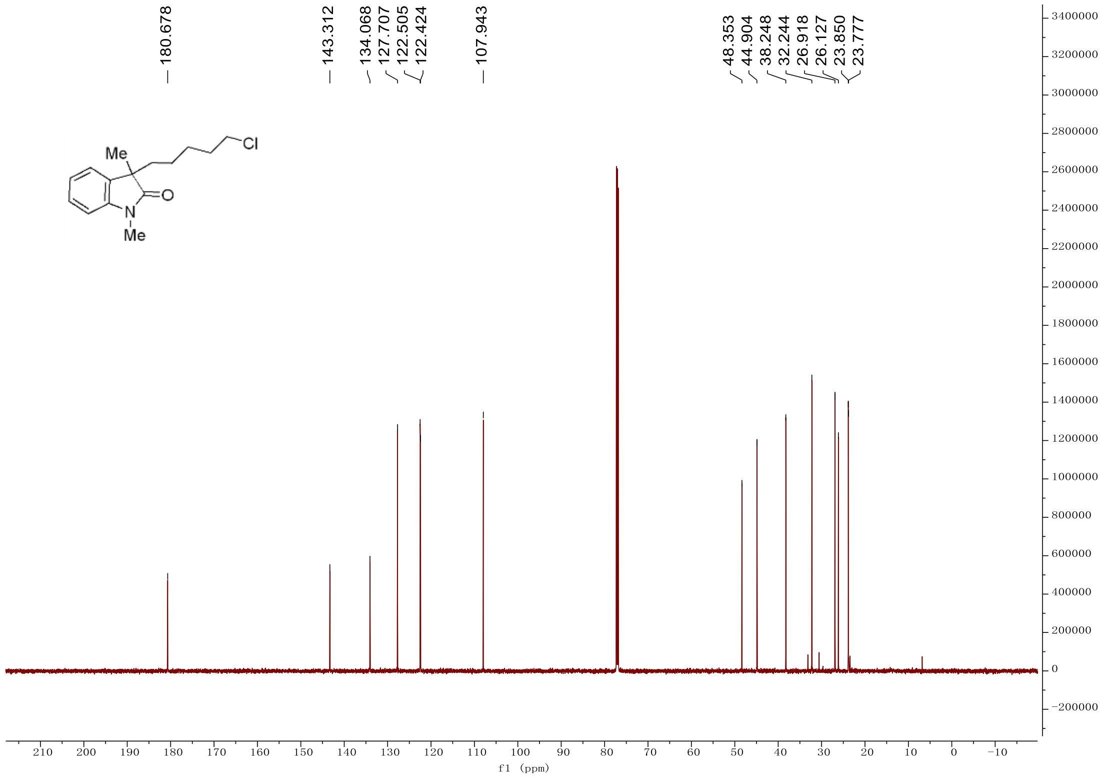

3ap ^{1}H NMR (600 MHz, Chloroform-*d*)

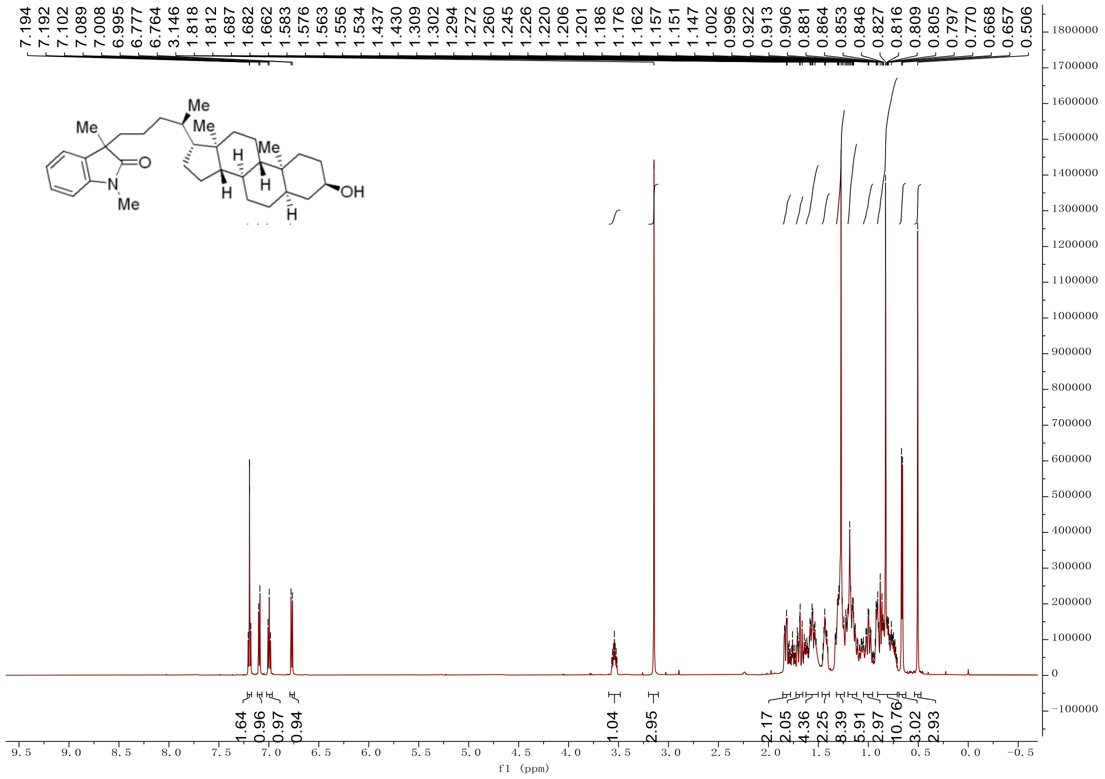

3ap ^{13}C NMR (151 MHz, Chloroform-*d*)

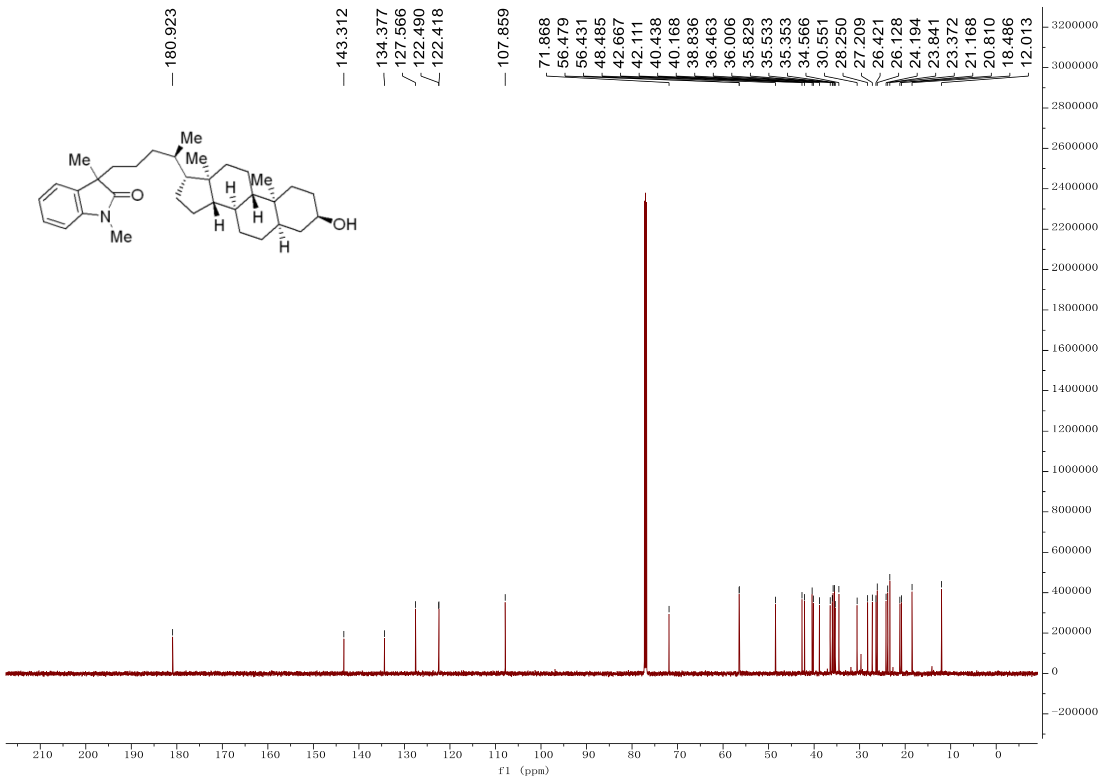

Supplement: File 1 — Experimental section and characterization of synthesized compounds. [file Beilstein_J_Org_Chem-19-57-s001.pdf]
